# Acid-Catalyzed Dehydrocoupling of Phosphines

**DOI:** 10.1021/acs.inorgchem.6c00218

**Published:** 2026-05-05

**Authors:** Zakary T. Ekstrom, Alexander M. Stone, Guobi Li, Hannah D. Hassoun, Anthony D. Kornokovich, Emalyn Delgado Rosario, Arturo Espinosa Ferao, Matthias Zeller, Arnold L. Rheingold, John D. Protasiewicz

**Affiliations:** † Department of Chemistry, 2546 Case Western Reserve University, 2080 Adelbert Road, Cleveland, Ohio 44106, United States; ‡ Departamento de Química Orgánica, Facultad de Química, Campus de Espinardo, 16751 Universidad de Murcia, 30100 Murcia, Spain; § Department of Chemistry, 8522 Purdue University, West Lafayette, Indiana 47907-2084, United States; ∥ Department of Chemistry and Biochemistry, 8784 University of California, San Diego, La Jolla, California 92093, United States

## Abstract

A new strategy for transition-metal-free, Bro̷nsted acid-catalyzed dehydrocoupling of P–H bonds in organophosphorus compounds is described. Using *para*-benzoquinones as hydrogen acceptors, this method enables room-temperature coupling of primary and secondary phosphines to form P–P-bonded products. *Ortho*-phosphinophenol (**PP**) and *ortho*-diphosphinobenzene (**DPB**) are efficiently converted to the heterocyclic dimers [C_6_H_4_P­(O)]_2_ (**DBODP**) and [C_6_H_4_P­(PH)]_2_ (**DBTP**), respectively, in the presence of catalytic quantities of strong Bro̷nsted acids (or AlCl_3_) and 2,5-di-*tert*-butyl-1,4-benzoquinone (**tBuBQ)**. **DBODP** and its ditungsten pentacarbonyl adduct were structurally characterized. The scope of the process extends to representative primary and secondary phosphines, and combined experimental/computational analyses support a mechanism in which acid activation of the quinone promotes hydride abstraction from phosphorus to generate a phosphinophosphenium intermediate that undergoes P–P bond formation and closes the catalytic cycle. This mild, acceptor-mediated platform complements established transition-metal and main-group approaches to P–P bond construction.

## Graphical abstract

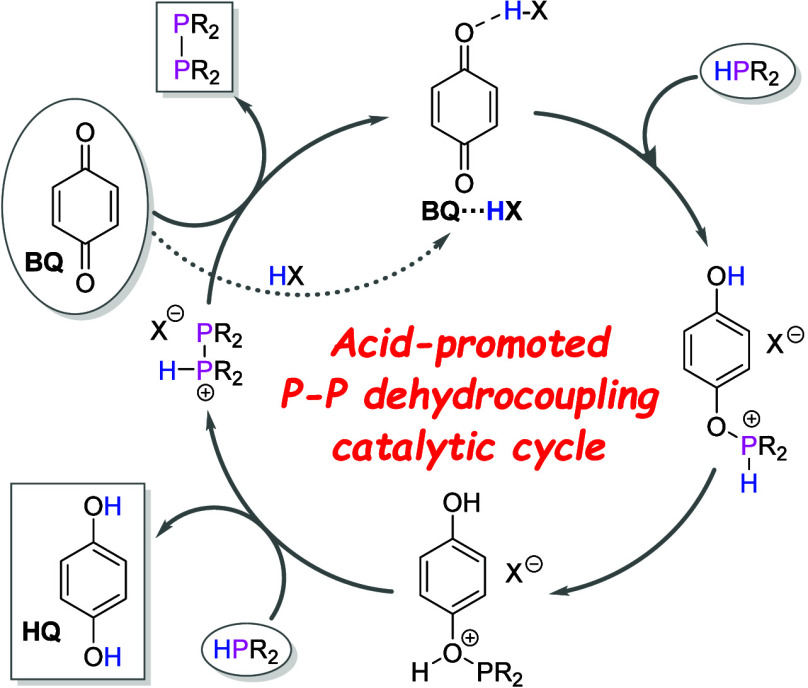

## Introduction

Dehydrocoupling reactions that form new bonds between main-group elements (E) by net loss of hydrogen offer a versatile entry to element–element bonded molecules and materials, including discrete dimers, cyclic oligomers, and polymers (Scheme 1).^1–20^ These transformations are commonly classified as acceptorless (H_2_ evolution) or acceptor A-enabled (hydrogen transfer to an oxidant or unsaturated acceptor), with both manifolds enabled by select catalysts.^12–19^

**Scheme 1:**
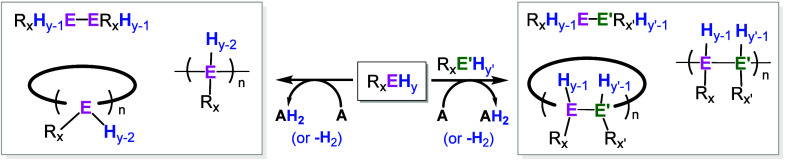
General Overview of Reported Homo- (Left) and Hetero- (Right) Dehydrocoupling Reactions

Phosphines are particularly attractive substrates because P–H bonds are typically weaker and more polarizable than C–H bonds, and phosphorus exhibits a strong propensity for catenation. Early transition-metal catalysis demonstrated that *ortho*-diphosphinobenzene (**DPB**) can undergo successive P–H dehydrocoupling to furnish benzotriphosphole-based oligomers and even a P_16_ macrocycle ([**DBTP**]_4_, **DBTP** = [C_6_H_4_P­(PH)]_2_).^20,21^ Cross-dehydrocoupling (heterodehydrocoupling) of phosphines with silanes under mild conditions further established the synthetic reach of P–H activation chemistry.^22^

More recently, nontransition-metal strategies—frustrated Lewis pairs, carbenoids, and alkali-metal bases—have expanded the dehydrocoupling toolkit, often requiring elevated temperatures and/or specialized acceptors.^23–29^ In parallel, our group has investigated functionalized benzoxaphospholes derived from *ortho*-phosphinophenol (**PP**), including conjugated P=C-containing fluorophores.^30–36^ During these studies, a serendipitous acid-promoted transformation revealed a previously underexplored opportunity: Bro̷nsted acid activation of a simple, commercially available *para*-benzoquinone can enable rapid, room-temperature, metal-free P–P bond formation.

Although phosphines are known to add to quinones (1,4- and 1,6-addition) to generate P–C and P–O products,^37,38^ instances where benzoquinones act as clean hydrogen acceptors in phosphine dehydrocoupling are scarce.^38–40^ Here we show that strong Bro̷nsted acids (or AlCl_3_) catalyze hydrogen transfer from primary and secondary phosphines to *para*-benzoquinones, delivering selective P–P coupling under mild conditions. We further delineate how substrate basicity, quinone identity, and ring-strain energetics shape reactivity, emphasizing the role of ring strain energy (RSE) in the relative thermodynamics of forming fused five-membered P-heterocycles.

## Results and Discussion

Our interest in P–H dehydrocoupling emerged during attempts to extend the synthesis of 2-substi-tuted-1,3-benzoxaphospholes (R-**BOP**) from **PP**.^30–36^ In an effort to access a 2-pyridyl analogue, a stepwise protocol—acylation of **PP** followed by addition of *p*-toluenesulfonic acid (*p*-TsOH)—unexpectedly yielded minor amounts of a crystalline product displaying a single ^31^P­{^1^H} resonance at δ 130.1 ppm. Single-crystal X-ray diffraction identified this compound as the benzoxadiphosphole dehydro-dimer [C_6_H_4_P­(O)]_2_ (**DBODP**), i.e., the formal dehydrocoupling product of **PP** (Scheme 2, Figure 1).

**Scheme 2:**
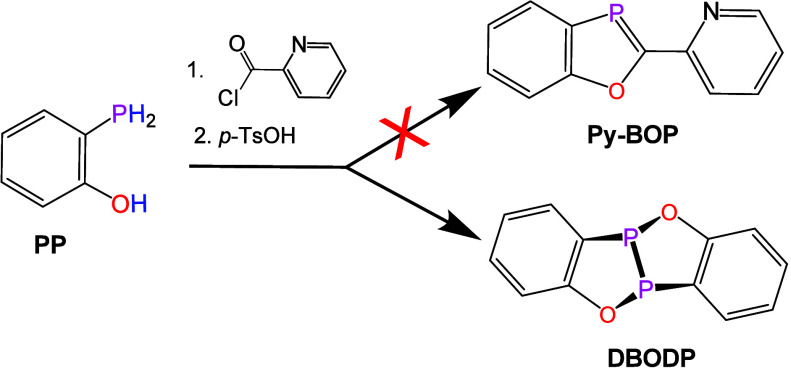
Serendipitous Formation of **DBODP** from **PP** under Acid-Promoted Conditions

**Figure 1:**
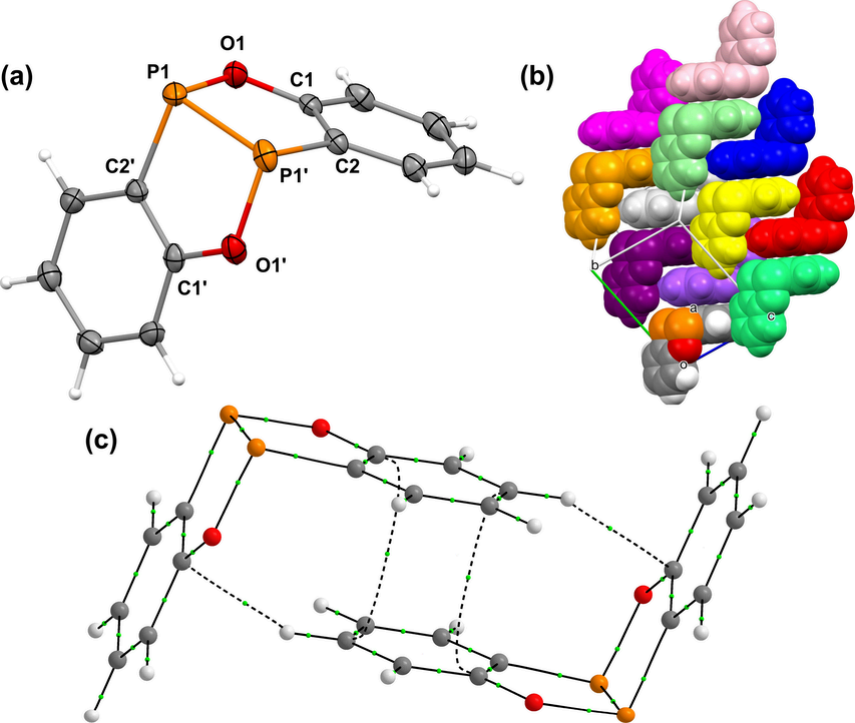
Solid-state molecular structure (a) and packing (b) of **DBODP**. (c) Visualization of noncovalent interactions (dashed lines for bond paths and small green spheres for bond critical points) between a pair of **DBODP** molecules in a gas-phase computed dimer.

Related diphosphorus-bridged heterocycles have been reported,^41–45^ including a sterically substituted benzoxadiphosphole isolated as a byproduct during distillation.^46^ The analogous **DPB**-derived (**DBP**: *ortho*-diphosphinobenzene) dehydrodimer **DBTP** exhibits a comparably folded global structure, although its five-membered rings are more puckered due to the larger size of P relative to O.^47^

Computational analyses support meaningful orthogonal T- and π-stacking interactions in the solid state, consistent with a Tetris-like packing motif (Figure 1c, see also Supporting Information). Related orthogonal noncovalent binding motifs have been described in μ^2^-pyrazolyl pyridylphenyl palladium complexes.^48^ Computations further indicate that **DBODP** is substantially more stable than a stereoisomer generated by inversion at one phosphorus center, with a large inversion barrier of 67.8 kcal/mol consistent with values reported for σ^3^λ^3^ phosphorus compounds.^49^

To probe the donor properties of **DBODP**, we examined its coordination chemistry with tungsten pentacarbonyl fragments. Reaction of **DBODP** with [W­(CO)_5_(MeCN)] afforded mono- and bis-adducts detectable by ^31^P NMR spectroscopy. The mononuclear complex displays ^31^P­{^1^H} resonances at δ 104.8 (^1^ *J*_PP_ = 220 Hz) and 136.4 (^1^ *J*_PP_ = 220, ^1^ *J*_PW_ = 290 Hz) ppm. The bis­(pentacarbonyl)­tungsten adduct [W­(CO)_5_]_2_ **DBODP** shows a single ^31^P­{^1^H} resonance at δ 125.5 ppm (^1^ *J*_PW_ = 207, ^2^ *J*_PW_ = 107 Hz) and was isolated in low yield and structurally characterized by X-ray diffraction (Figure 2). Coordination induces only minor changes to the **DBODP** framework (≤0.01 Å contraction of the P–P bond and a modest decrease by 4.6° in fold angle), broadly consistent with the behavior of related P–P-bonded ligands upon binding W­(CO)_5_ fragments.^50^

**Figure 2:**
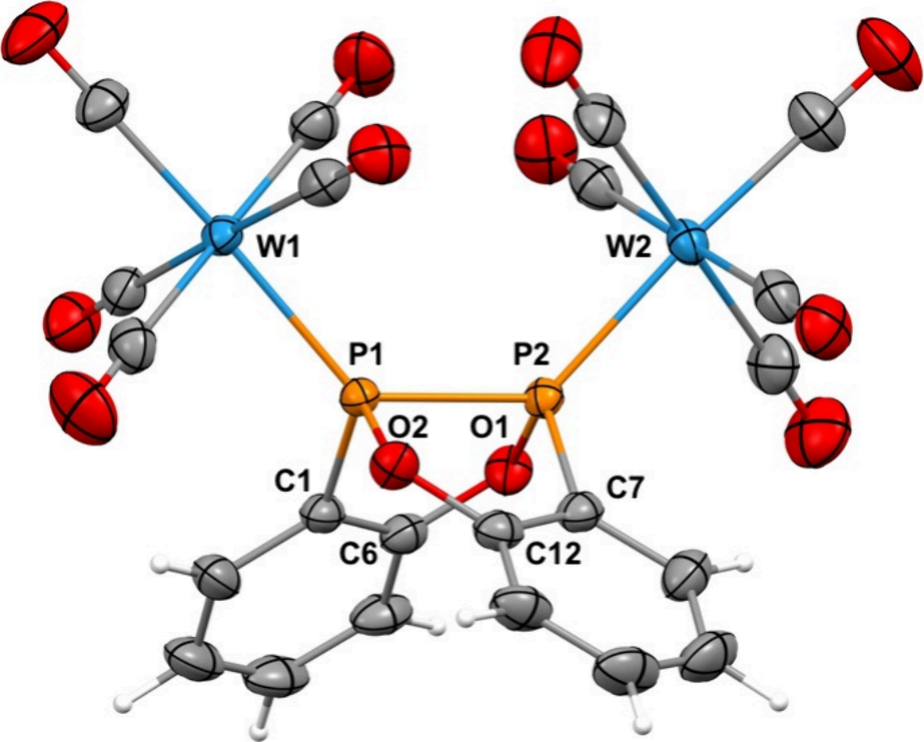
Solid-state molecular structure of [W­(CO)_5_]_2_ **DBODP**.

Previous work on **PP** has shown that low-energy conformations can display short PH···HO contacts consistent with dihydrogen bonding, a feature that has been proposed to correlate with enhanced hydrogen activation or release in main-group systems.^51^ Motivated by this observation, we briefly examined whether analogous noncovalent interactions might be present in related substrates such as *ortho*-diphosphinobenzene (**DPB**) and *ortho*-phosphinoaniline (**PAN**). While only weak and highly conformation-dependent PH···HP or PH···HN contacts were identified in these systems, their analysis provides useful qualitative insight into subtle intramolecular factors that may influence hydrogen transfer processes. A detailed computational analysis of these conformational and noncovalent interaction effects is provided in the Supporting Information.

Because **DBODP** formed under ostensibly metal-free conditions, we investigated whether *para*-benzoquinones could act as practical hydrogen acceptors to enable intentional, catalytic dehydrocoupling. Oxidized guanidino-functionalized aromatics (GFAs) can promote dehydrocoupling of phosphines at elevated temperature,^52–54^ and quinone-mediated H_2_ removal from vicinal P–H units has also been demonstrated.^55^ However, reactions of phosphines with *para*-benzoquinones typically favor addition chemistry at C=O or ring positions rather than clean hydrogen transfer.^37,38^

Initial reactions of **PP** with DDQ, BQ, or duroquinone produced **DBODP** as a major product but were incomplete, and/or unselective, and products proved impossible to separate and purify from coproduct hydroquinones. In contrast, 2,5-di*tert*-butyl-1,4-benzoquinone (tBuBQ) provided improved solubility profiles facilitating separation from the reduced hydroquinone, as well as displaying fewer competing addition pathways, presumably due to steric shielding of the ring positions. Nevertheless, without catalysis, reactions could not be driven to completion either thermally or photochemically, and isolated yields of **DBODP** seemed capped at 35%.

Strikingly, adding catalytic quantities of strong Bro̷nsted acids (e.g., HOTf, HBF_4_·Et_2_O, HCl·Et_2_O) or AlCl_3_ dramatically accelerated hydrogen transfer, yielding **DBODP** as the sole ^31^P NMR detectable organophosphorus product within minutes at room temperature (Scheme 3, Table S1). Weaker acids (e.g., TFA) were less effective, and AcOH was completely ineffective. AlCl_3_ likely operates via in situ generation of HCl upon protonolysis by **PP**, as suggested in related aluminum­(III) catalysis contexts.^41^ Moderate to high yields were also obtained in the analogous Bro̷nsted acid-catalyzed reaction of **BDP** (Table S2). By contrast, **PAN** is unreactive toward **tBuBQ** over a 24-h period even in the presence of 10 mol % triflic acid. In the presence of 10 mol % AlCl_3_, **PAN** is consumed over the same 24-h period and several products are formed, as ascertained by ^31^P NMR spectroscopy. One of the major species displays a singlet at δ 45.8 ppm, which we have tentatively assigned as the proposed dehydrocoupled dimer based on DFT predicted chemical shift for minimized structure of **DBADP** (see Supporting Information). Unfortunately, we have not yet been able to successfully isolate this material from the reaction mixture to allow for rigorous identification

**Scheme 3:**
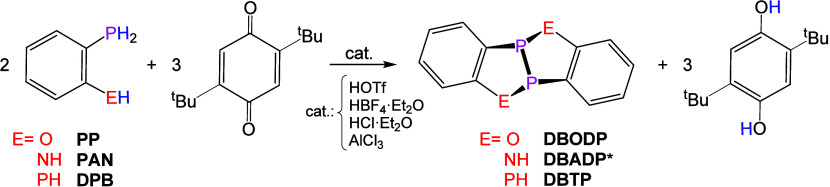
Dehydrocoupling Reactions of **PP**, **PAN,** and **DPB** Using **tBuBQ** in the Presence of Various Catalysts for Potential Formation of Dehydrodimers of the Form [C_6_H_4_P­(EH)]_2_ ^a^

a *Tentatively assigned.

With optimized conditions in hand, we evaluated representative secondary and primary phosphines. Diphenylphosphine (Ph_2_PH) underwent rapid coupling to Ph_2_P-PPh_2_ in the presence of tBuBQ and catalytic HOTf, whereas dicyclohexylphosphine (Cy_2_PH) did not undergo detectable dehydrocoupling under any tested conditions. Primary phosphines (PhPH_2_, CyPH_2_) afforded mixtures of cyclic oligophosphines [RP]_*n*_ (*n* = 4–6), with PhPH_2_ generally reacting faster and more selectively than CyPH_2_ (Table 1). Specific product ring distributions might also be influenced if, after initial phosphorus–phosphorus bond formation dehydrocoupling catalysis, secondary ring interconversions processes can be promoted by acids.^56^ See the Supporting Information for a complete set of results.

**Table 1:** Substrate Scope and Optimized Conditions for the Dehydrocoupling of Phosphines with TBuBQ after 10 Min Reaction Time^a^

	Ph_2_PH	PhPH_2_	CyPH_2_
HOTf	Ph_2_P-PPh_2_ (88%)	[PhP]_4_ (3%)	[CyP]_4_ (29%)
		[PhP]_5_ (93%)	[CyP]_5_ (24%)
		[PhP]_6_ (2%)	
	1%,^b^ 1.5 mol,^c^	1%,^b^ 1 mol,^c^	1%,^b^ 3 mol,^c^
	{100%}^d^	{99%}^d^	{100%}^d^
HBF_4_·Et_2_O	Ph_2_P-PPh_2_ (62%)	[PhP]_4_ (6%)	[CyP]_4_ (30%)
		[PhP]_5_ (88%)	[CyP]_5_ (50%)
		[PhP]_6_ (1%)	
	1%,^b^ 1.5 mol,^c^	3%,^b^ 1.5 mol,^c^	1%,^b^ 3 mol,^c^
	{69%}^d^	{100%}^d^	{84%}^d^
HCl·Et_2_O	Ph_2_P-PPh_2_ (72%)	[PhP]_4_ (5%)	[CyP]_4_ (75%)
		[PhP]_5_ (94%)	
		[PhP]_6_ (1%)	
	5%,^b^ 1.5 mol,^c^	2%,^b^ 1.5 mol,^c^	5%,^b^ 3 mol,^c^
	{90%}^d^	{100%}^d^	{100%}^d^
AlCl_3_	Ph_2_P-PPh_2_ (90%)	[PhP]_4_ (4%)	[CyP]_4_ (100%)
		[PhP]_5_ (94%)	
		[PhP]_6_ (1%)	
	10%,^b^ 1.5 mol,^c^	10%,^b^ 1 mol,^c^	10%,^b^ 3 mol,^c^
	{94%}^d^	{99%}^d^	{100%}^d^

a Main products (parentheses, %) estimated by ^31^P­{^1^H} NMR spectroscopy.

b Catalyst loading.

c Quinone load (per mol of phosphine).

d Phosphine consumption in curly brackets (%).

Excess **tBuBQ** often improved reaction rates and yields for sluggish substrates, consistent with competitive protonation equilibria between phosphines and the quinone acceptor (*vide infra*). Noteworthily, if the amounts of **tBuBQ** and acid catalyst (1–5 mol % HCl) are increased, the reaction of **DPB** becomes unselective and appears promote runaway polymerization of **DPB**, as indicated by generation of very broad ^31^P NMR resonances in the general region reported for [**DBTP**]_4_.^20^

To contextualize reactivity trends, we compared computed thermodynamics for dehydrocoupling of *ortho*-functionalized arylphosphines C_6_H_4_PH_2_(EH) (E = O, NH, PH) to form fused dehydrodimers [C_6_H_4_P­(E)]_2_, **DBODP**, **DBADP** and **DBTP** (Table 2). The computed energetics (gas phase, M062X/def2-TZVP) show that, across the series, formation of the PH-bridged dimer is most favorable, with O and NH analogues progressively less favorable. It is important to note that ring strain energy (RSE) provides a mechanistic and thermodynamic rationale for these differences: homodesmotic reaction^57^ analyses, similar to those reported for P-containing saturated^58–63^ or unsaturated^64,65^ three- or four-membered rings,^66^ indicate that computed (PWPB95-D3/def2-QZVPP//PBEh-3c) RSE increases in the order PH < O < NH, and the presence of two fused five-membered rings amplifies these effects.

**Table 2:** Computed Energies (kcal/mol) for Dehydrocoupling Processes and RSE (kcal/mol) for One of the Central Rings in Final Dehydrodimers

	**C**_**6**_ **H**_**4**_ **PH**_**2**_ **(EH) → [C**_**6**_ **H**_**4**_ **P(E)]**_**2**_ **+ 3H**_**2**_		
**E:**	**O**	**NH**	**PH**
Δ*H*	1.4	7.5	–8.8
Δ*G*	–8.4	–4.0	–17.7
RSE	1.9	8.1	0.2

These findings underscore that ring strain is not merely a structural curiosity but a decisive energetic lever governing selectivity in dehydrocoupling and heterocycle formation, particularly in fused P-heterocycles. The results also correlate with the decreased propensity for **PAN** to undergo clean dehydrocoupling relative to the other *ortho*-phenylphosphines under our conditions.

The role of acid is best interpreted as activation of the hydrogen acceptor rather than direct activation of the phosphine. Herein computed (B3LYP-D3/6-311G++(2d,p)) gas-phase proton affinities (PAs, kcal/mol) for selected phosphines and *para*-benzoquinones decrease in the order Ph_2_P-PPh_2_ (237.2) > Cy_2_PH (232.9) Me_2_P-PMe_2_ (229.9) > Ph_2_PH (225.9) > CyPH_2_ (213.9) ≥ **PP** (213.8) ≥ **DBP** (213.1) ≥ **PAN** (213.0, for P-protonation) > PhPH_2_ > tBuBQ (205.6) > BQ (193.1), in line with the reported measured or calculated PAs values for some of these species.^67–72^ The above PA values show that secondary phosphines are more basic than primary phosphines, and that Cy_2_PH is among the most basic substrates examined.^68–74^ This trend aligns with the observed inhibition of Ph_2_PH dehydrocoupling when Cy_2_PH is present (no Cy_2_PPPh_2_ was formed either), consistent with preferential acid sequestration by Cy_2_PH.

Although computed PAs can favor P-protonation over quinone protonation, solvent and hydrogen-bonding effects can stabilize quinone–acid adducts sufficiently to initiate hydride abstraction. Protonation/hydrogen bonding is well-known to enhance quinone electrophilicity and redox behavior.^75–79^ We therefore propose that the catalytically competent oxidant is a protonated or strongly hydrogen-bonded benzoquinone (BQ···HX or [BQ–H]^+^X^–^) that abstracts hydride from the phosphine to form a phosphinophosphenium (or related diphosphonium) intermediate en route to P–P bond formation.

Computational analysis of a model system supports a pathway (Scheme 4) in which acid-activated quinone (BQ···HOTf) promotes formation of a reactive P-centered cation (**II**) that, after P-to-O proton shift (**I**), couples with a second phosphine, ultimately regenerating the activated quinone. Such intermediates are consistent with known donor-stabilized phosphenium and phosphinophosphenium chemistry.^80–90^

**Scheme 4:**
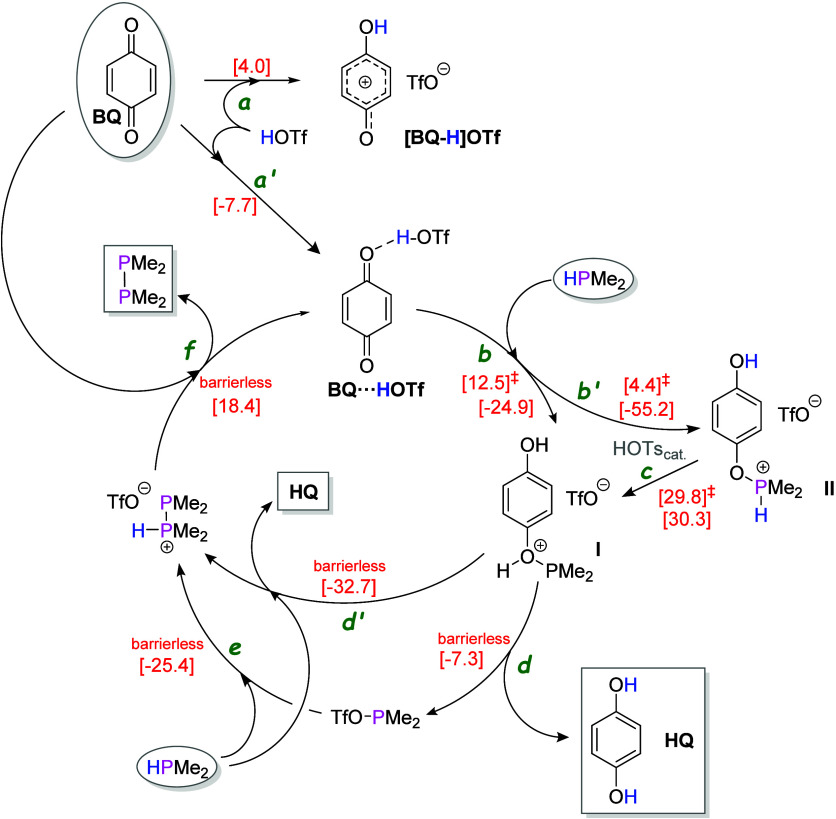
Proposed Model Acid-Catalyzed Hydrogen Transfer/Dehydrocoupling with Para-Benzoquinones^a^

a Computed [CPCM­(CH_2_Cl_2_)/PWPB95-D3/def2-QZVPP//CPCM­(CH_2_Cl_2_)/PBEh-3c] zero-point corrected energies (kcal/mol) for the individual steps, in red square brackets. The symbol ^‡^ is used for designating energy barriers.

Since the C=O bonds of tBuBQ can be activated by acid for hydrogen transfer, one can reasonably expect that N=N of azobenzenes and C=N bonds of quinodiimides could be utilized in these reactions. Indeed, azobenzene was found to be an equally effective hydrogen acceptor as **tBuBQ** in reactions with **PP**. Interestingly, replacing tBuBQ with 2,3,5,6-tetramethyl-*N*,*N*′**-diphenylcyclohexa-2,5-diene-1,4-diimine (*N*,*N′*-diphenyl-duroquinone-1,4-diimine, DQI) as the hydrogen acceptor allowed the reaction of **PP** to proceed at room temperature to selectively afford **DBODP** in around ten minutes *without any added acid*. The corresponding reaction of **DPB** with DQI does not proceed without acid catalyst (but does so in the presence of 1 mol % triflic acid), despite having significantly higher driving force (*vide supra*). It thus appears that the combination of **PP** having the acidic phenol functionality (while **DPB** is not acidic) allows **PP** to act as its own catalyst to initiate the reaction by proton transfer to the basic DQI (calculated PA DQI: 242.5 kcal/mol).^91^ This finding thus lends further credence to a proposed mechanism involving initial activation of the hydrogen acceptor. A radical pathway analogous to acid-catalyzed reduction of tBuBQ by 1,4-cyclohexadienes has been discussed in related contexts.^92^ However, the rapid and selective conversion of **PP** to **DBODP** in the presence of a sterically hindered phenolic radical scavenger argues against a dominant radical chain process under these conditions.^93–95^

## Conclusions

We report a mild, transition-metal-free strategy for P–P bond formation via Bro̷nsted acid-catalyzed hydrogen transfer from phosphines to para-benzoquinone acceptors. Under optimized conditions using tBuBQ, **PP** and **DPB** are rapidly converted at room temperature to the fused heterocyclic dehydrodimers **DBODP** and **DBTP**, respectively, and the method extends to representative primary and secondary phosphines. Structural characterization of **DBODP** and its [W­(CO)_5_]_2_ adduct establishes key geometric features of this benzoxadiphosphole platform. Combined experimental/computational analyses support a mechanism in which acid activation of the quinone promotes hydride abstraction and generates a reactive P-centered cation that enables P–P bond formation and catalyst turnover. Thermochemical analyses further highlight ring strain energy as a governing factor in the relative stability of fused dehydrodimers. This acceptor-mediated, metal-free dehydrocoupling approach complements existing transition-metal and main-group protocols and provides a practical entry to P–P-bonded architectures under exceptionally mild conditions. It also expands on recent work that shows that even olefins can act as hydrogen acceptors with generation of SiE bonds (E=O, N, or S) from silanes.^96^

## Experimental Section

### General Considerations

All reactions were performed under an atmosphere of nitrogen using standard Schlenk line techniques or in a MBraun glovebox. Unless otherwise stated, all chemicals were purchased from commercial sources and used without further purification. *ortho*-Phosphinophenol (**PP**),^97^ *ortho*-phosphinoaniline (**PAN**),^98^ and *N*,*N′*-diphenyl-duraquinone-diimine (**DQI**)^99,100^ were prepared as described previously. DCM was purified using an MBraun SPS-5. NMR spectra were collected using a Bruker AVANCE III 500 spectrometer. Chemical shifts were internally referenced to residual solvent signals (^1^H, ^13^C) or externally 85% H_3_PO_4_ (^31^P). Elemental Analysis was performed by Robertson Microlit Laboratories (Ledgewood, NJ). No uncommon hazards are noted.

#### Synthesis of DBODP by Dehydrocoupling of PP with tBuBQ

A dry 50 mL Schlenk flask was charged with 2-phosphinophenol (**PP**) (0.341 g, 2.70 mmol) and anhydrous DCM (15 mL) under nitrogen. A solution of 2,5-di-*tert*-butyl-1,4-benzoquinone (**tBuBQ**) (0.914 g, 4.06 mmol) in anhydrous DCM (10 mL) was then added via syringe. The flask was fitted with a septum and static nitrogen and left to stir overnight at room temperature (25–30 °C). The next morning a white precipitate in a yellow solution was observed and an aliquot was taken to be analyzed via ^31^P NMR spectroscopy. The corresponding spectra showed that the reaction was nearly complete. The reaction was then filtered via gravity filtration to separate the white solid and the filtrate was collected. The filtrate was then concentrated leaving a yellow solid. The solid was then suspended in a 1:1 mixture of Et_2_O:Hexanes and cooled to −78 °C at which a white precipitate was observed. The reaction was filtered to collect the solid. The solids were washed with additional ether (2 × 5 mL) and dried under high vacuum. Yield of **DBODP**: 0.232 g (35%) colorless crystalline solid. ^1^H NMR (500 MHz, CDCl_3_) δ = 7.94 (m, *J* = 7.6, 3.1, 1.7, 0.6 Hz, 2H), 7.35 (m, *J* = 8.9, 7.2, 1.6 Hz, 2H), 7.07 (m, *J* = 7.3, 1.1 Hz, 2H), 7.03 (d, *J* = 7.8 Hz, 2H). ^31^P {^1^H} NMR (202 MHz, CDCl_3_) δ = 130.1. ^13^C {^1^H} NMR (126 MHz, CDCl_3_) δ = 163.0 (t, *J* = 6.4 Hz), 134.5 (t, *J* = 16.3 Hz), 134.0, 127.0 (d, *J* = 21.8 Hz), 122.5 (t, *J* = 3.6 Hz), 116.3. Anal. Calcd for C_12_H_8_O_2_P_2_: C, 58.56; H, 3.28. Found: C, 58.27; H, 3.31.

#### Synthesis of DBODP by Dehydrocoupling of PP with tBuBQ in the Presence of AlCl_3_

A dry 50 mL Schlenk flask was charged with **PP** (1.31 g, 10.4 mmol) and anhydrous DCM (15 mL). A solution of tBuBQ (3.43 g, 15.6 mmol) in DCM (10 mL) was then added via syringe. The reaction mixture was transferred to a glovebag filled with N_2_ where a catalytic amount of AlCl_3_ (5 mol %) was added. The reaction mixture was left to stir for 10 min where a white precipitate was observed in a yellow solution. The flask was removed from the glovebag and the reaction was then filtered via gravity filtration to separate the white solid and the filtrate was collected. The filtrate was then concentrated leaving a yellow solid. The solid was then suspended in a 1:1 mixture of Et_2_O:Hexanes and cooled to −78 °C at which a white precipitate was observed. The reaction was filtered to collect the solid. The solids were washed with additional ether (2 × 5 mL) and dried under high vacuum. Yield of **DBODP**: 0.996 g (78%) white crystalline solid.

#### Synthesis of [W­(CO)_5_]_2_DBODP

A 25 mL Schlenk flask was charged with **DBODP** (0.200 g, 0.811 mmol) and acetonitrile pentacarbonyltungsten (0.608 g, 1.67 mmol). Dry THF (15 mL) was then added, and a gold solution was observed. The reaction was then stirred for 17 h at room temperature. Volatiles were then removed under vacuum to give a yellow-green solid. Purification via recrystallization with THF and *n*-pentane afforded yellow-green crystals. Isolated yield: 0.524 g, 72.1%. ^1^H NMR (500 MHz, CD_2_Cl_2_) δ = 7.87–7.81 (m, 2H), 7.48 (t, *J* = 7.7 Hz, 2H), 7.27 (t, *J* = 7.5 Hz, 2H), 7.10 (d, *J* = 8.3 Hz, 2H). ^31^P {^1^H} NMR (202 MHz, CD_2_Cl_2_) δ = 125.5 (^1^ *J*_PW_ = 207 Hz, ^2^ *J*_PW_ = 107 Hz). ^13^C­{^1^H} NMR (126 MHz, CD_2_Cl_2_) δ = 197.6 (t, *J* = 18.3 Hz, *trans*-CO), 194.7 (t, *J*_PC_ = 3.3 Hz, *J*_CW_ = 125.5 Hz *cis*-CO), 159.2, 135.9, 132.5 (t, *J* = 9.8 Hz), 127.7 (t, *J* = 13.8 Hz), 124.7 (t, *J* = 4.5 Hz), 117.0 (t, *J* = 4.0 Hz). IR (ν_CO_, cm^–1^): 2075 (m), 1901 (vs). Anal. Calcd for C_22_H_8_O_12_P_2_W_2_: C, 29.56; H, 0.90. Found: C, 29.79; H, 0.83.

#### General Procedure for Reactions of C_6_H_4_PH_2_(EH) (E=O or PH) with tBuBQ in the Presence of Various Lewis/Bro̷nsted Acid Catalysts

To a solution of with C_6_H_4_PH_2_(EH) and anhydrous DCM was added a solution of **tBuBQ** in DCM via syringe. The reaction mixture was transferred to a glovebag where the catalyst (1–10 mol %) was then added. The reaction mixtures were left to stir (see Section 1.4, Tables S1 and S2, in the Supporting Information for full details) to produce a mixture of a white precipitate and a light-yellow solution. Analyses of reaction mixtures via ^31^P­{^1^H} NMR spectroscopy afforded estimates of the yields of dehydrodimers **DBODP** and **DBTP**. Analysis of the white precipitate by NMR spectroscopy revealed no other organophosphorus compounds, and showed the presence of the hydrogenated byproduct of **tBuBQ**, 2,5-di-*tert*-butyl-1,4-hydroquinone.

#### General Procedure for Reactions of Primary and Secondary Phosphines with tBuBQ in the Presence of Various Lewis/Bro̷nsted Acid Catalysts

In a glovebox, the primary or secondary phosphines and anhydrous DCM were added to a pressure tube with Teflon screw-top cap. A solution of **tBuBQ** in anhydrous DCM was then added. The reaction mixture was transferred to a glovebag where an acid catalyst was added. The reaction mixture was then left to stir for 10 min or longer (see Section 1.5, Tables S6–S10, in the Supporting Information for full details) before an aliquot was taken for ^31^P­{^1^H} NMR spectroscopy.

### Computational Details

DFT calculations related to **PP** dehydro oligomers (**DBODP**, **DBODP’**), ring strain energy evaluation and energetics for the P–P dehydro coupling catalytic cycle were performed with the ORCA program.^101^ All geometry optimizations were run in redundant internal coordinates in the gas phase (unless otherwise stated), with tight convergence criteria. Harmonic frequency calculations verified the nature of ground states or TS having all real (positive) frequencies or only one imaginary (negative) frequency, respectively. For the study of **PP** dehydro oligomers, optimizations were performed employing the B3LYP^102,103^ functional together with the RIJCOSX algorithm,^104^ the Ahlrichs segmented def2-TZVP basis set^105^ and the 2010 Grimme’s semiempirical atom-pairwise London dispersion correction (DFT-D4).^106^ From these optimized geometries, all reported energies were corrected for the zero-point vibrational term and obtained by means of single-point (SP) calculations using the more extensive and polarized def2-QZVPP^107^ basis set and the recently developed near-linear scaling domain-based local pair natural orbital (DLPNO) method^108^ to achieve coupled cluster theory with single, double, and perturbative triple excitations (CCSD­(T)).^109^ Solvent (CHCl_3_) effects were taken into account with the CPCM solvation model.^110,111^ In case of RSE studies and the mechanism of the P–P dehydrocoupling catalytic cycle, optimizations were carried out using Grimme’s PBEh-3c^112^ composite functional, final energies being evaluated with the double-hybrid-meta-GGA functional PWPB95 ^113,114^ with Grimme’s D3 semiempirical atom-pairwise correction,^115,116^ which makes use of the Becke-Johnson rationale damping,^117–119^ and the def2-QZVPP basis set. RSEs are obtained by averaged (zero point-corrected) energy evaluation of appropriate homodesmotic reactions,^120^ corresponding to all possible endocyclic A-B bond cleavage reactions using HA-BH reagents. Given the focus on an overall RSE tendency in the comparison of [C_6_H_4_P­(E)]_2_ dehydrodimers (**DBTP**, **DBODP**, and **DBADP**, for E=PH, O and NH, respectively), the homodesmotic P-E bond cleavage alone was evaluated. It is important to note that P–Ar and E-Ar bond cleavage homodesmotic reactions would result in products featuring long-range interactions introducing uncompensated factors on both sides of the homodesmotic reaction, which would distort the obtained strain energy value. In this regard, it is reasonable to assume the unstrained character of the second five-membered ring remaining in the resulting homodesmotic P-E bond cleavage product (**HD**_**P‑Ecleav**_). Overall energetics for dehydrocoupling reactions (Table 1) and conformational analysis for **DPB** and **PAN** were conducted with the Gaussian^121^ software package using the Minnesota M062X functional^122^ and the def2-TZVP basis set. Proton affinities (PA) were estimated at the B3LYP-D3/6-311++G­(2d,p) level, using the expression PA = −Δ*E*_ZPE_ + ^5^/_2_(RT). Computed NMR values were obtained with the gauge-independent atomic orbital (GIAO) method,^123,124^ using the PBE1PBE^125^ functional, the 6-311G++(2d,2p) basis set and the IEFPCM^126^ solvation (CHCl_3_) model. AIM analysis was performed with Multiwfn^127^ software using the B3LYP-D3/6-311++G­(2d,p) electron density. These electron densities were also employed in the computation of noncovalent interactions with the NCIplot^128–130^ program.

## Supplementary Material



## Notes

Experimental procedures, computational details, and characterization data for all reactions and products, including ^1^H, ^13^C­{^1^H}, and ^31^P NMR spectra (PDF)

## ⊥

Deceased March 3rd, 2024.
