# Supplementary material for: Acid-Catalyzed Dehydrocoupling of Phosphines
Source: Inorg Chem. 2026 May 5;65(19):10571–80. doi: 10.1021/acs.inorgchem.6c00218 (PMC13188061; doi:10.1021/acs.inorgchem.6c00218)
Supplement: Supplementary file 1 [file ic6c00218_si_001.pdf]

Electronic Supporting Information

For

Acid-Catalyzed Dehydrocoupling of Phosphines

Zakary T. Ekstrom,[a] # Alexander M. Stone,[a] # Guobi Li,[a] Hannah D. Hassoun,[a] Anthony D. Kornokovich,[a] Emalyn Delgado Rosario,[a] Arturo Espinosa Ferao,*[b] Matthias Zeller,[c] Arnold L. Rheingold,[d] and John D. Protasiewicz*[a]

[a] Z. T. Ekstrom, A. M. Stone, G. Li, H. D. Hassoun, A. D. Kornokovich, E. Delgado Rosario, J. D. Protasiewicz*
Department of Chemistry
Case Western Reserve University
2080 Adelbert Road, Cleveland, Ohio 44106, United States
E-mail: protasiewicz@case.edu

[b] A. Espinosa Ferao*
Departamento de Química Orgánica, Facultad de Química
Campus de Espinardo, Universidad de Murcia
30100 Murcia, Spain
E-mail: artuesp@um.es

[c] M. Zeller
Department of Chemistry
Purdue University
West Lafayette, Indiana 47907-2084, United States

[d] A. L. Rheingold†
Department of Chemistry and Biochemistry
University of California, San Diego
La Jolla, California 92093, United States

†Deceased March 3rd, 2024

# These authors contributed equally

Contents

**1 Experimental**	S3
1.1 General Experimental Details	S3
1.2 Initial reactions of **PP**	S3
1.3 Preparation of $[W(CO)_5]_2$**DBODP**	S8
1.4 Survey of reactions of $C_6H_4PH_2(EH)$ (E = O, NH, or PH)	S10
1.5 Reactions of secondary and primary phosphines	S17
1.6 Alternative hydrogen acceptors	S30
**2. X-Ray Diffraction Analyses**	S35
**3. Computational Details**	S42
3.1 General Details	S42
3.2 Ring Strain Energy Evaluation	S42
3.3 Analysis of Crystal Packing Forces in **DBODP**	S42
3.4 Inversion at P in **DBODP**	S47
3.5 Consideration of other possible dehydrocoupling products of **PP**	S48
3.6 Conformational Analysis and Dihydrogen Bonding in **DPB** and **PAN**	S51
3.7 Structures and Energies for dehydrocoupling reactions	S61
3.8 Calculated ^{31}P NMR shifts for dehydrocoupled dimers of $C_6H_4PH_2(EH)$	S66
3.9 Structures and energies for proton affinities	S67
3.10 Structures and energies for proposed mechanism	S77
**4. References**	S86

1 Experimental

1.1 General Experimental Details

All reactions were performed under an atmosphere of nitrogen using standard Schlenk line techniques or in a MBraun glove box. Unless otherwise stated, all chemicals were purchased from commercial sources and used without further purification. *ortho*-Phosphinophenol (**PP**)[1], *ortho*-phosphinoaniline (**PAN**)[2], and N,N'-diphenyl-duraquinone-diimine (**DQI**)[3] were prepared as described previously. DCM was purified using an MBraun SPS-5. NMR spectra were collected using a Bruker AVANCE III 500 spectrometer. Chemical shifts were internally referenced to residual solvent signals (^{1}H, ^{13}C) or externally 85% H_3PO_4 (^{31}P). Elemental Analysis was performed by Robertson Microlit Laboratories (Ledgewood, NJ).

1.2 Initial reactions of PP

a. Reaction of *ortho*-phosphinophenol (PP) with 2,5-di-*tert*-butyl-1,4-benzoquinone (tBuBQ), no catalyst, at room temperature.

A dry 50 mL Schlenk flask was charged with 2-phosphinophenol (**PP**) (0.341 g, 2.70 mmol) and anhydrous DCM (15 mL) under nitrogen. A solution of 2,5-di-*tert*-butyl-1,4-benzoquinone (**tBuBQ**) (0.914 g, 4.06 mmol) in anhydrous DCM (10 mL) was then added via syringe. The flask was fitted with a septum and static nitrogen and left to stir overnight at room temperature (25 – 30 °C). The next morning a white precipitate in a yellow solution was observed and an aliquot was taken to be analyzed via ^{31}P NMR spectroscopy. The corresponding spectra showed that the reaction was nearly complete. The reaction was then filtered via gravity filtration to separate the white solid and the filtrate was collected. The filtrate was then concentrated leaving a yellow solid. The solid was then suspended in a 1:1 mixture of Et_2O:hexanes and cooled to -78 °C at which a white precipitate was observed. The reaction was filtered to collect the solid. The solids were washed with additional ether (2 x 5 mL) and dried under high vacuum. Mass: 0.232 g (35%) colorless crystalline solid. ^{1}H NMR (500 MHz, $CDCl_3$) δ = 7.94 (m, *J* = 7.6, 3.1, 1.7, 0.6 Hz, 2H), 7.35 (m, *J* = 8.9, 7.2, 1.6 Hz, 2H), 7.07 (m, *J* = 7.3, 1.1 Hz, 2H), 7.03 (d, *J* = 7.8 Hz, 2H). ^{31}P {^{1}H} NMR (202 MHz, $CDCl_3$) δ = 130.1. ^{13}C {^{1}H} NMR (126 MHz, $CDCl_3$) δ = 163.0 (t, *J* = 6.4 Hz), 134.5 (t, *J* = 16.3 Hz), 134.0, 127.0 (d, *J* = 21.8 Hz), 122.5 (t, *J* = 3.6 Hz), 116.3. Anal. Calcd for $C_{12}H_8O_2P_2$: C, 58.56; H, 3.28. Found: C, 58.27; H, 3.31.

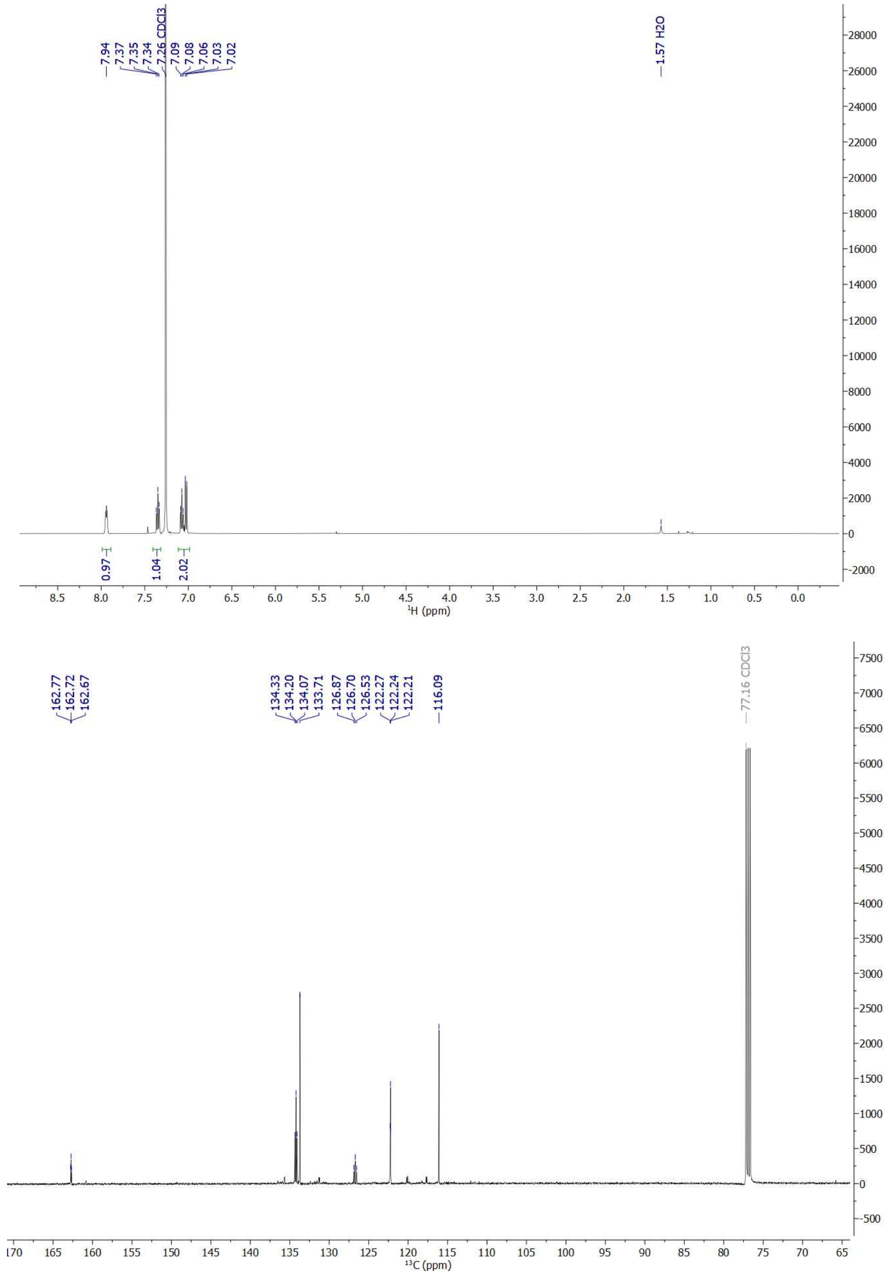

Figure S1: ^1H (top) and $^{13}C\{^1H\}$ (bottom) NMR Spectra of **DBODP** ($CDCl_3$, 202 MHz, 298 K).

b. Reaction of PP with duraquinone (DQ), no catalyst, at room temperature.

A dry 200 mL Schlenk flask was charged with **PP** (2.05 g, 16.2 mmol) under argon. Anhydrous DCM (75 mL) was added before adding a DCM solution (25 mL) of **DQ** (4.03 g, 24.5 mmol). The flask was fitted with a septum and static argon supply and stirred overnight at rt, yielding a beige suspension. The volatiles were removed under reduced pressure before transferring the now dark purple solid to the glovebox. The solid was stirred in fresh DCM (15 mL) before filtering to remove the suspended solids and washing the filter cake with additional DCM (2 x 2 mL). The filtrate was concentrated and stirred in an additional portion of

DCM (15 mL) before filtering and concentrating the filtrate to a dark purple solid. The resulting solid was suspended in ether and stirred before filtering. The collected solids were washed with additional ether (2 x 2 mL) and the solids dried under high vacuum (0.557 g, 13.7%) as an impure mixture of **DBODP** and durohydroquinone.

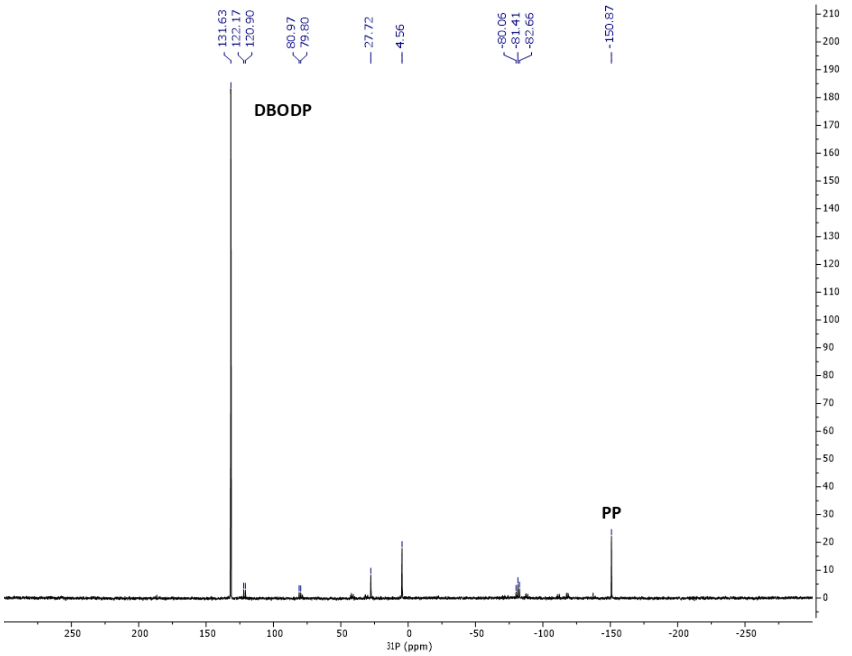

Figure S2: $^{31}P\{^1H\}$ NMR Spectrum of the reaction of **PP** (2 eq) with **DQ** (3 eq) and no catalyst (DCM, 202 MHz, 298 K).

c. Reaction of PP with *para*-benzoquinone (BQ), no catalyst, at room temperature.

A dry 50 mL Schlenk flask was charged with **PP** (0.591 g, 4.69 mmol) under argon. Anhydrous DCM (20 mL) was added before adding a DCM solution (10 mL) of **BQ** (0.760 g, 7.03 mmol). During the addition, the solution became cloudy then turned dark green in color. The reaction was stirred at room temperature for 12 hours (overnight). The resulting suspension faded to a colorless solution with a beige-orange precipitate. $^{31}P\{^1H\}$ NMR showed only partial conversion to **DBODP**.

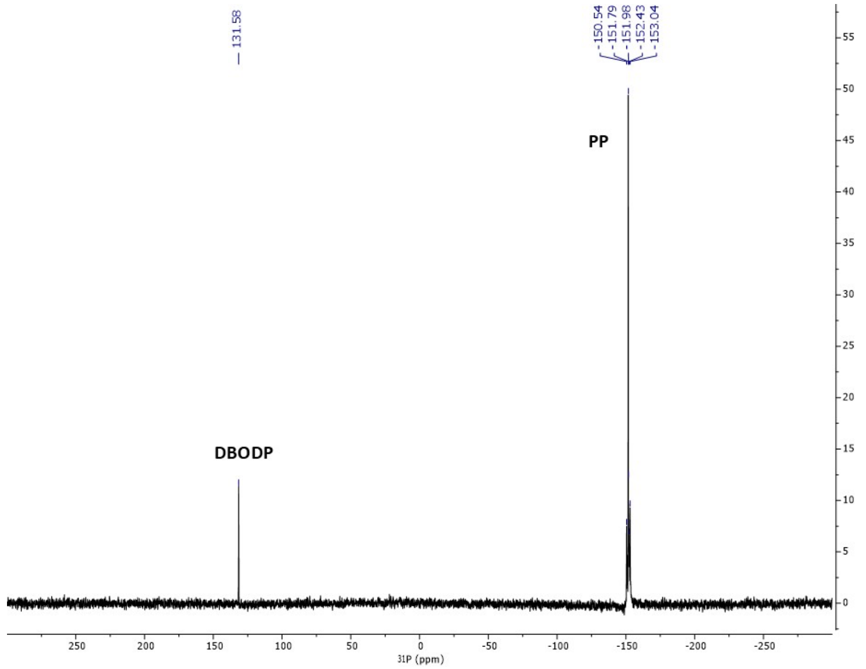

Figure S3: $^{31}P\{^{1}H\}$ NMR Spectrum of the reaction of **PP** (2 eq) with **BQ** (3 eq) and no catalyst (DCM, 202 MHz, 298 K).

d. Reaction of PP with 2,3-dichloro-5,6-dicyano-*p*-benzoquinone (DDQ), no catalyst, at room temperature.

A dry 50 mL Schlenk flask was charged with **PP** (0.061 g, 0.48 mmol) under argon. Anhydrous DCM (10 mL) was added before adding a DCM solution (20 mL) of **DDQ** (0.110 g, 0.73 mmol). The reaction was stirred overnight at room temperature. The $^{31}P\{^{1}H\}$ NMR spectrum showed only partial conversion to **DBODP** with a second unidentified product at δ167.7 ppm.

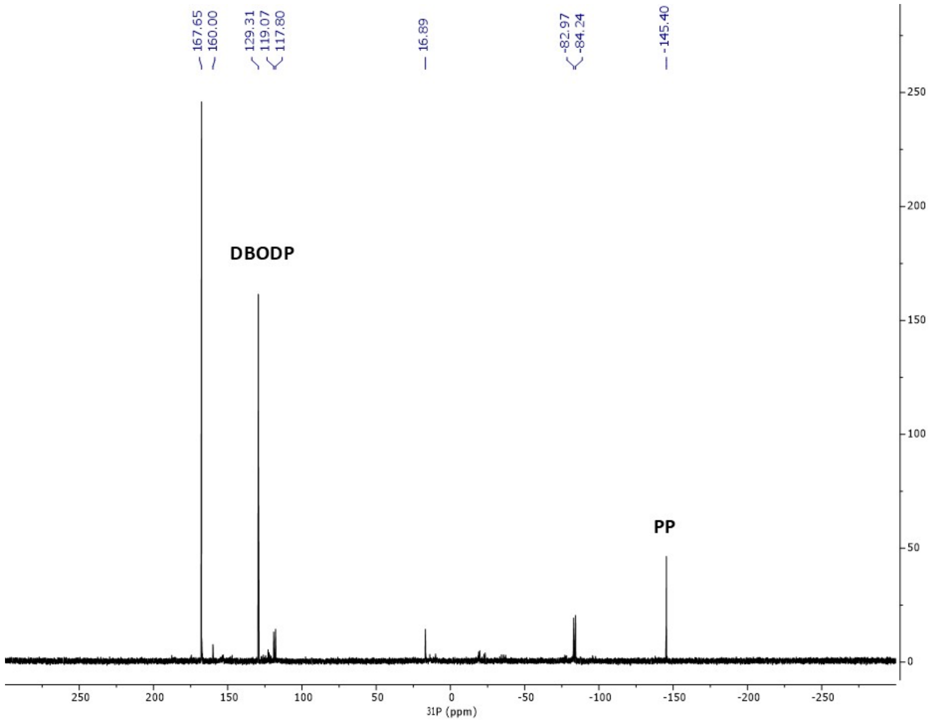

Figure S4: $^{31}P\{^1H\}$ NMR Spectrum of the reaction of **PP** (2 eq) with **DDQ** (3 eq) and no catalyst (DCM, 202 MHz, 298 K).

e. Reaction of PP with tBuBQ, with catalytic amounts of $AlCl_3$, at room temperature.

A dry 50 mL Schlenk flask was charged with **PP** (1.31 g, 10.4 mmol) and anhydrous DCM (15 mL). A solution of **tBuBQ** (3.43 g, 15.6 mmol) in DCM (10 mL) was then added via syringe. The reaction mixture was transferred to a glovebag filled with N_2 where a catalytic amount of $AlCl_3$ (5 mol %) was added. The reaction mixture was left to stir for 10 minutes where a white precipitate was observed in a yellow solution. The flask was removed from the glovebag and the reaction was then filtered via gravity filtration to separate the white solid and the filtrate was collected. The filtrate was then concentrated leaving a yellow solid. The solid was then suspended in a 1:1 mixture of Et_2O:hexanes and cooled to -78 °C at which a white precipitate was observed. The reaction was filtered to collect the solid. The solids were washed with additional ether (2 x 5 mL) and dried under high vacuum. Mass: 0.996 g (78 %) white crystalline solid.

1.3 Preparation of $[W(CO)_5]_2$DBODP .

A 25 mL Schlenk flask was charged with **DBODP** (0.200 g, 0.811 mmol) and acetonitrile pentacarbonyltungsten (0.608 g, 1.67 mmol). Dry THF (15 mL) was then added, and a gold solution was observed. The reaction was then stirred for 17 hours at room temperature. Volatiles were then removed under vacuum to give a yellow-green solid. Purification via recrystallization with THF and *n*-pentane afforded yellow-green crystals. Isolated yield: 0.524 g, 72.1 %. ^{1}H NMR (500 MHz, CD_2Cl_2) δ = 7.87 – 7.81 (m, 2H), 7.48 (t, J = 7.7 Hz, 2H), 7.27 (t, J = 7.5 Hz, 2H), 7.10 (d, J = 8.3 Hz, 2H). ^{31}P {^{1}H} NMR (202 MHz, CD_2Cl_2) δ = 125.5 ($^{1}J_{PW}$ = 207 Hz, $^{2}J_{PW}$ = 107 Hz). ^{13}C{^{1}H} NMR (126 MHz, CD_2Cl_2) δ = 197.6 (t, J = 18.3 Hz, *trans*-CO), 194.7 (t, J_{PC} = 3.3 Hz, J_{CW} = 125.5 Hz *cis*-CO), 159.2, 135.9, 132.5 (t, J = 9.8 Hz), 127.7 (t, J = 13.8 Hz), 124.7 (t, J = 4.5 Hz), 117.0 (t, J = 4.0 Hz). IR (ν_{CO}, cm^{-1}): 2075 (m), 1901 (vs). Anal. Calcd for $C_{22}H_8O_{12}P_2W_2$: C, 29.56; H, 0.90. Found: C, 29.79; H, 0.83.

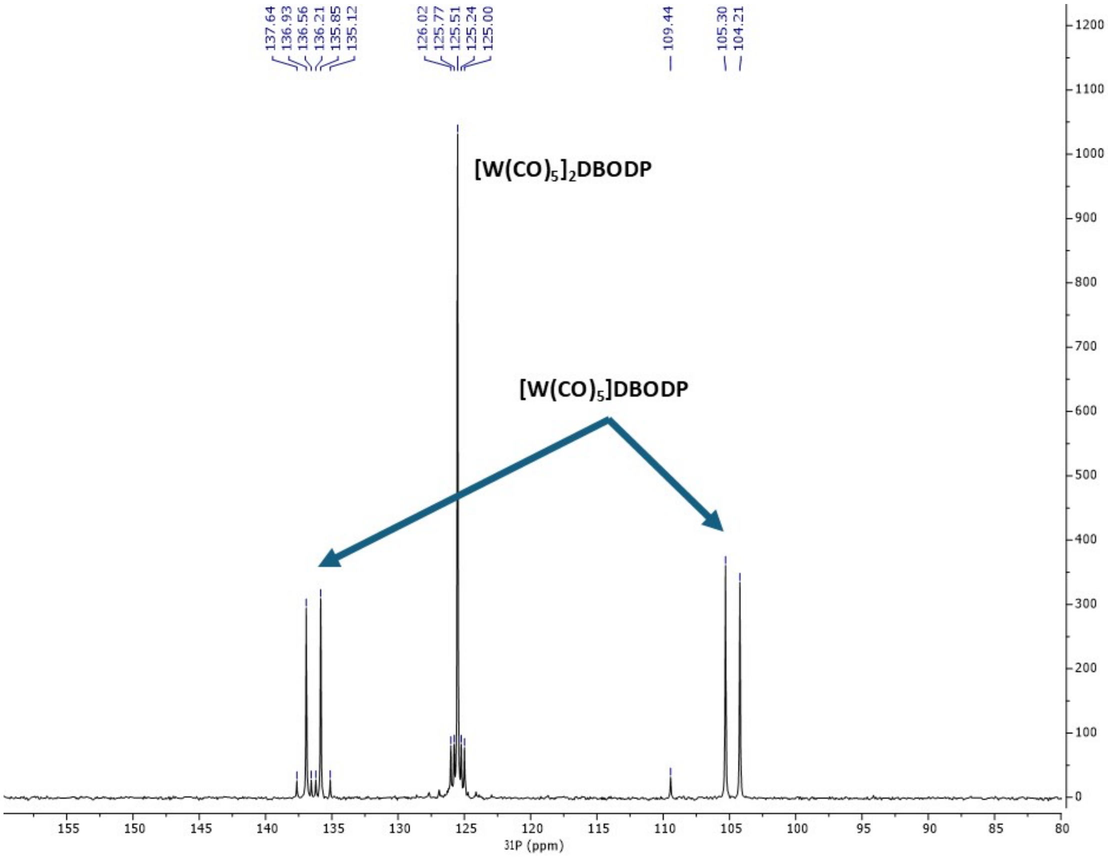

Figure S5: ^{31}P{^{1}H} NMR Spectrum of **$[W(CO)_5]$DBODP** and **$[W(CO)_5]_2$DBODP** ($CDCl_3$, 202 MHz, 298 K) prior to separation and isolation of **$[W(CO)_5]_2$DBODP**.

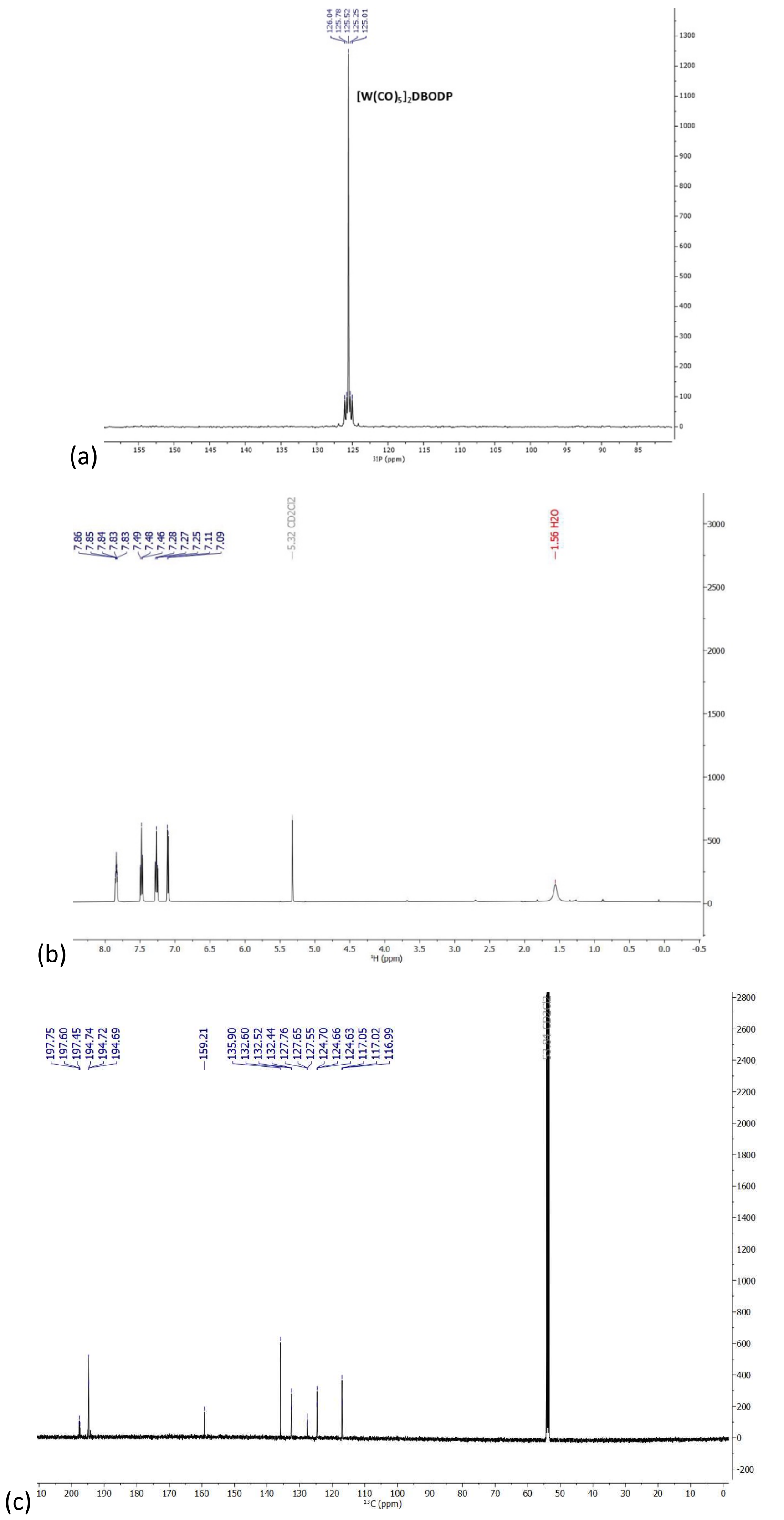

Figure S6: $^{31}P\{^1H\}$ (a), ^1H (b) and $^{13}C\{^1H\}$ (c) NMR Spectra of **$[W(CO)_5]_2$DBODP** ($CDCl_3$, 202 MHz, 298 K).

1.4 Survey of reactions of $C_6H_4PH_2(E)$ (E = O, NH, or PH)

a. *ortho*-phosphinophenol (PP)

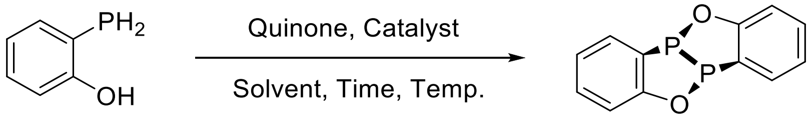

General procedure for Dehydrocoupling of PP with Lewis/Brønsted acid catalysts

A dry 50 mL Schlenk flask was charged with **PP** (10 mmol) and anhydrous DCM (15 mL). A solution of **tBuBQ** (15 mmol) in DCM (10 mL) was then added via syringe. The reaction mixture was transferred to a glovebag where the catalyst (1 mol % Triflic Acid, 1 mol % HBF_4•Et_2O, or 1 mol % HCl•Et_2O) was then added. The reaction mixture was left to stir between 10 – 30 minutes (**Table S1**) where a white precipitate was observed in a light-yellow solution. Analysis via $^{31}P\{^1H\}$ NMR spectroscopy showed quantitative formation of **DBODP**. Further analysis of the white precipitate show no presence of other organophosphorus compounds and confirmed it to be the hydrogenated byproduct of **tBuBQ**, 2,5-di-*tert*-butyl-1,4-hydroquinone.

Table S1: Reaction conditions for **DBODP** formation

Entries	PP: tBuBQ	Catalyst	Catalyst Amount	Conditions	Reaction Time	Conversion of PP	Yield of DBODP*
1	2:3	Triflic Acid	1 mol %	DCM, rt	10 min	100 %	100 %
2	2:3	HBF_4•Et_2O	1 mol %	DCM, rt	30 min	100 %	98 %
3	2:3	HCl (in Et_2O)	1 mol %	DCM, rt	10 min	100 %	100 %
4	2:3	$AlCl_3$	5 mol %	DCM, rt	10 min	100 %	100 %
5	2:3	*p*-TsOH	5 mol %	DCM, rt	10 min	100 %	100 %

***** yield was determined by the integration of $^{31}P\{^1H\}$ spectra.

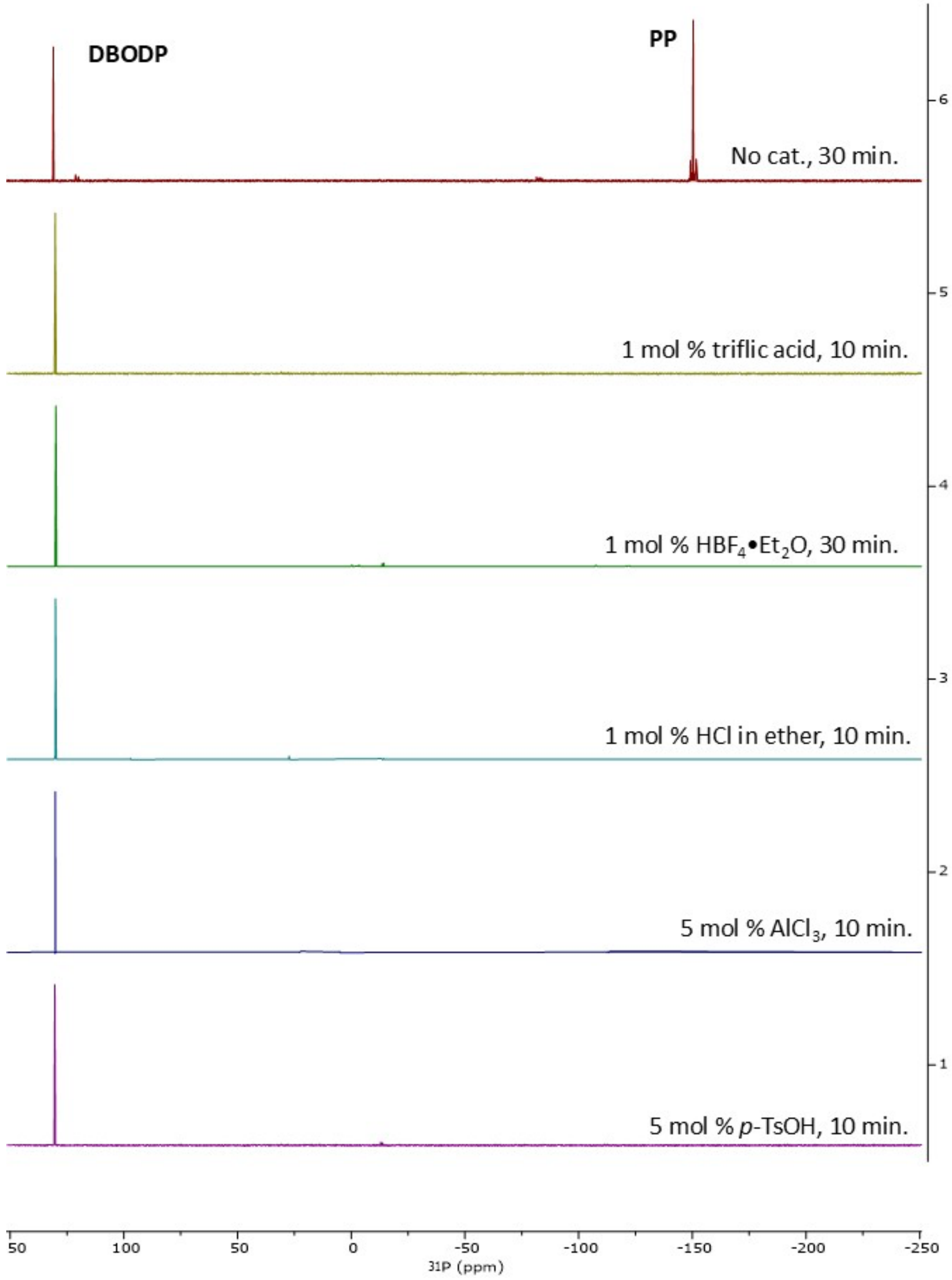

Figure S7: $^{31}P\{^{1}H\}$ NMR Spectra of the reaction of **PP** (2 eq) with **tBuBQ** (3 eq) and various catalysts (DCM, 202 MHz, 298 K).

b. 1,2-diphosphinobenzene (DPB)

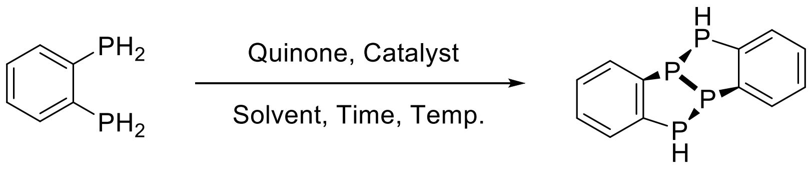

General procedure for Dehydrogenative Coupling of **DPB** with Lewis/Brønsted acid catalysts

A dry 15 mL pressure tube with a Teflon screw cap was charged with **DPB** (0.36 mmol) and anhydrous DCM (4 mL). A solution of **tBuBQ** (0.54 mmol or 1.08 mmol) in anhydrous DCM (4 mL) was added via syringe. The tube was transferred to a glovebag where the catalyst (1 mol % Triflic Acid, 1 mol % HBF_4•Et_2O, 1-5 mol % HCl (in Et_2O), 5 mol % $AlCl_3$, and 10 mol % *p*-TsOH) was then added. The reaction mixture was left to stir between 5 minutes and 24 hours (**Table S2**) where a white precipitate was observed in a light-yellow solution. Analysis of each reaction was done via $^{31}P\{^1H\}$ NMR spectroscopy showing formation of **DBTP**.

Table S2: Reaction conditions for **DBTP** formation

Entries	DPB: tBuBQ	Catalyst	Catalyst Amount	Conditions	Reaction Time	Conversion of DPB	Yield of DBTP*
1	2:3	--	--	DCM, rt	24 hr	0 %	0 %
2	2:3	Triflic Acid	1 mol %	DCM, rt	10 min	97 %	97 %
3	2:6	Triflic Acid	1 mol %	DCM, rt	10 min	100 %	0 %**
4	2:3	HBF_4•Et_2O	1 mol %	DCM, rt	30 min	81 %	81 %
5	2:3	HCl (in Et_2O)	1 mol %	DCM, rt	5 min	89 %	88 %
6	2:3	HCl (in Et_2O)	5 mol %	DCM, rt	10 min	100 %	0 %**
7	2:3	$AlCl_3$	5 mol %	DCM, rt	10 min	92 %	57 %
8	2:3	*p*-TsOH	10 mol %	DCM, rt	10 min	90 %	77 %

* yield was determined by the integration of $^{31}P\{^1H\}$ spectra.

** due to presumed runaway polymerization (**Figures S8-9**)

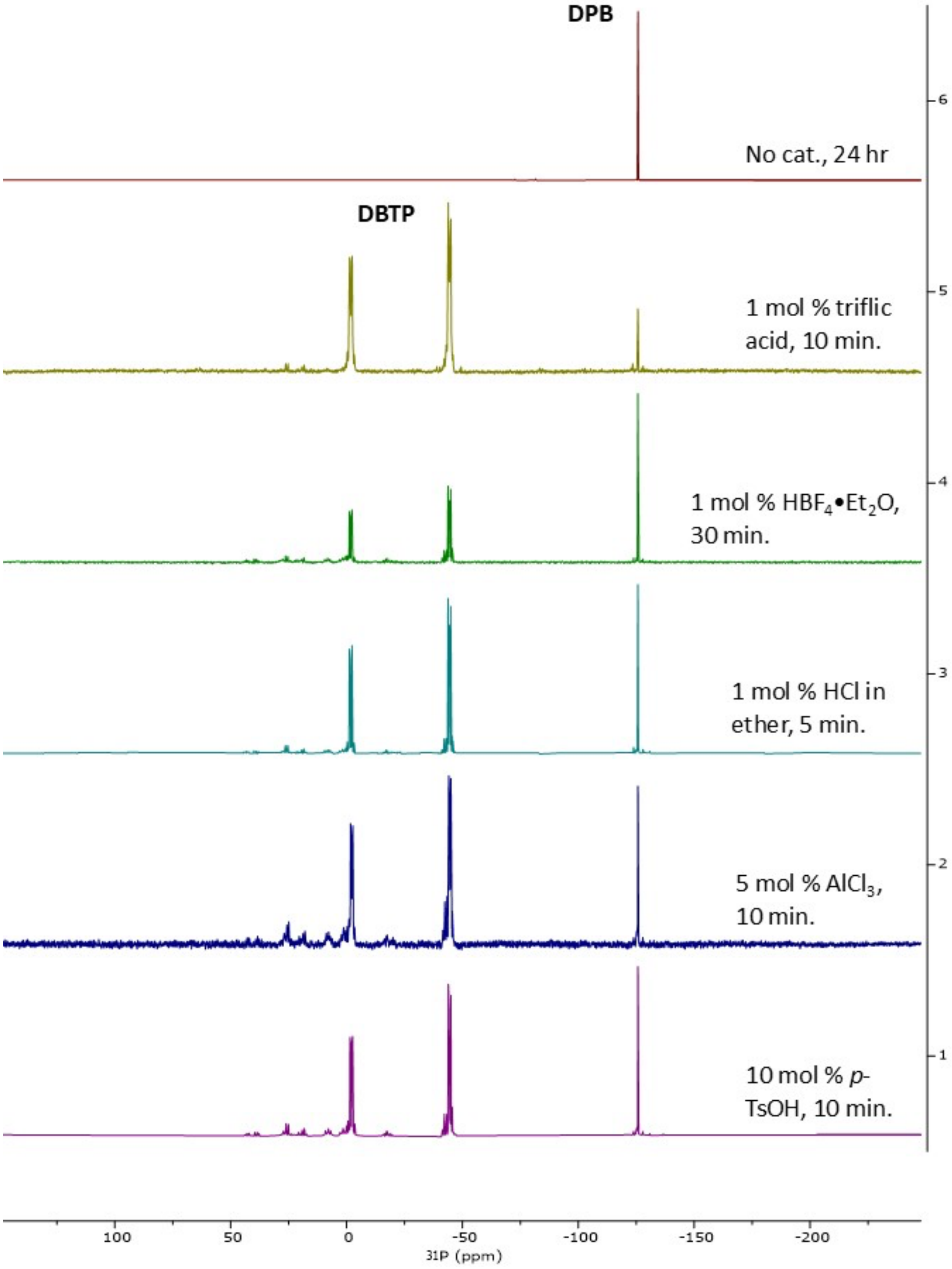

Figure S8: $^{31}P\{^{1}H\}$ NMR Spectra of the reaction of **DPB** (2 eq) with **tBuBQ** (3 eq) and various catalysts (DCM, 202 MHz, 298 K).

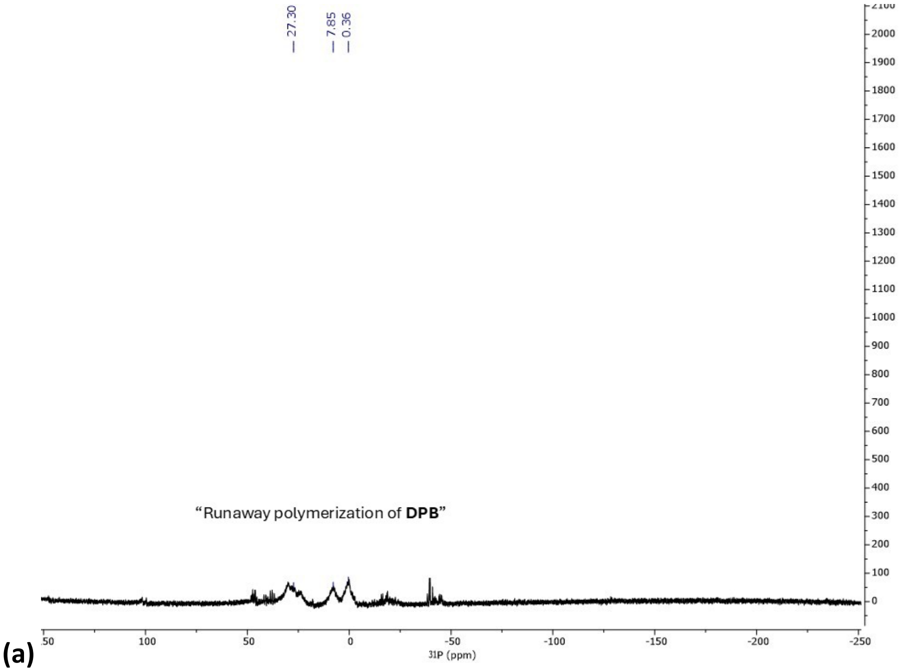

(a)

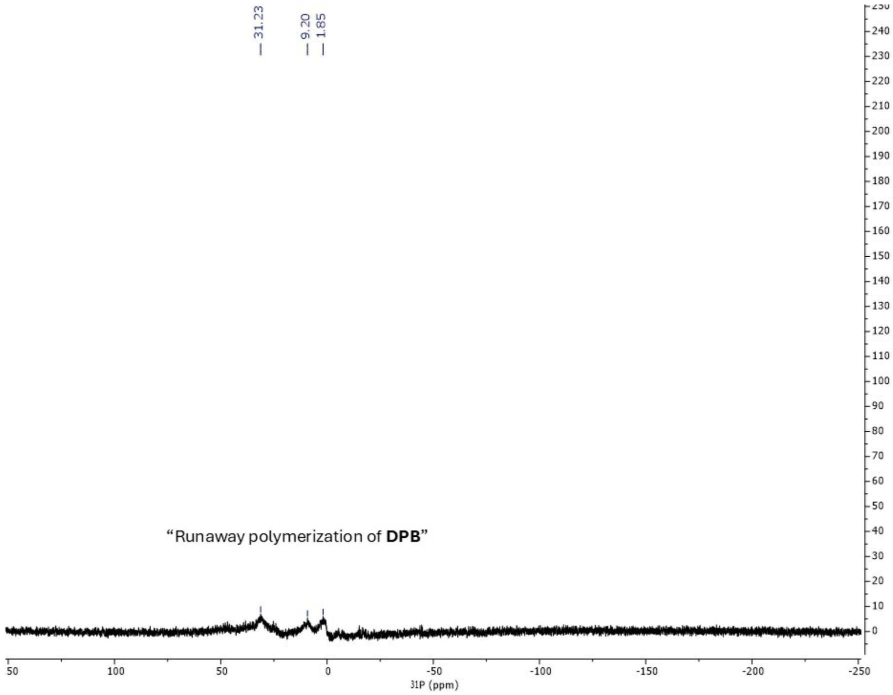

(b)

Figure S9: (a) $^{31}P\{^1H\}$ NMR Spectrum of the reaction of **DPB** (2 eq) with **tBuBQ** (6 eq) and 1 mol % triflic acid (DCM, 202 MHz, 298 K). (b) $^{31}P\{^1H\}$ NMR Spectrum of the reaction of **DPB** using 5 mol % 2M HCl (in Et_2O) and 0.54 mmol **tBuBQ** (DCM, 202 MHz, 298 K)

c. 2-phosphinoaniline (PAN)

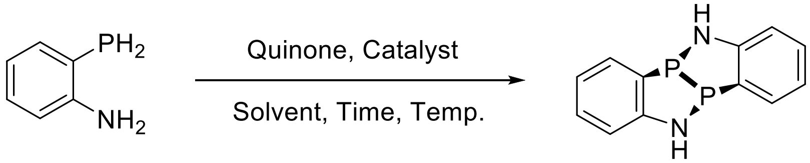

Scheme S5: Attempted preparation of benzazadiphosphole (**DBADP)** from **PAN**

Attempted Dehydrogenative Coupling of **PAN** with Lewis/Brønsted acid catalysts

A dry 50 mL Schlenk flask was charged with **PAN** (1 eq) and anhydrous DCM (10 mL). A solution of **tBuBQ** (1.5 eq) in dry DCM (10 mL) was added via syringe. The flask was transferred to a glovebag where 10 mol % $AlCl_3$ was then added. The reaction mixture was left to stir overnight (18 hours) (**Table S3)** where an aliquot was taken for $^{31}P\{^1H\}$ NMR spectral analysis.

Table S3: Reaction conditions for **DBADP** formation

**Entries**	**PP: tBuBQ**	**Catalyst**	**Catalyst Amount**	**Conditions**	**Reaction Time**	**Conversion of PAN**	**Yield of DBADP**
1	2:3	$AlCl_3$	10 mol %	DCM, rt	18 h	100 %	71 %

***** yield was determined by the integration of $^{31}P\{^1H\}$ spectra.

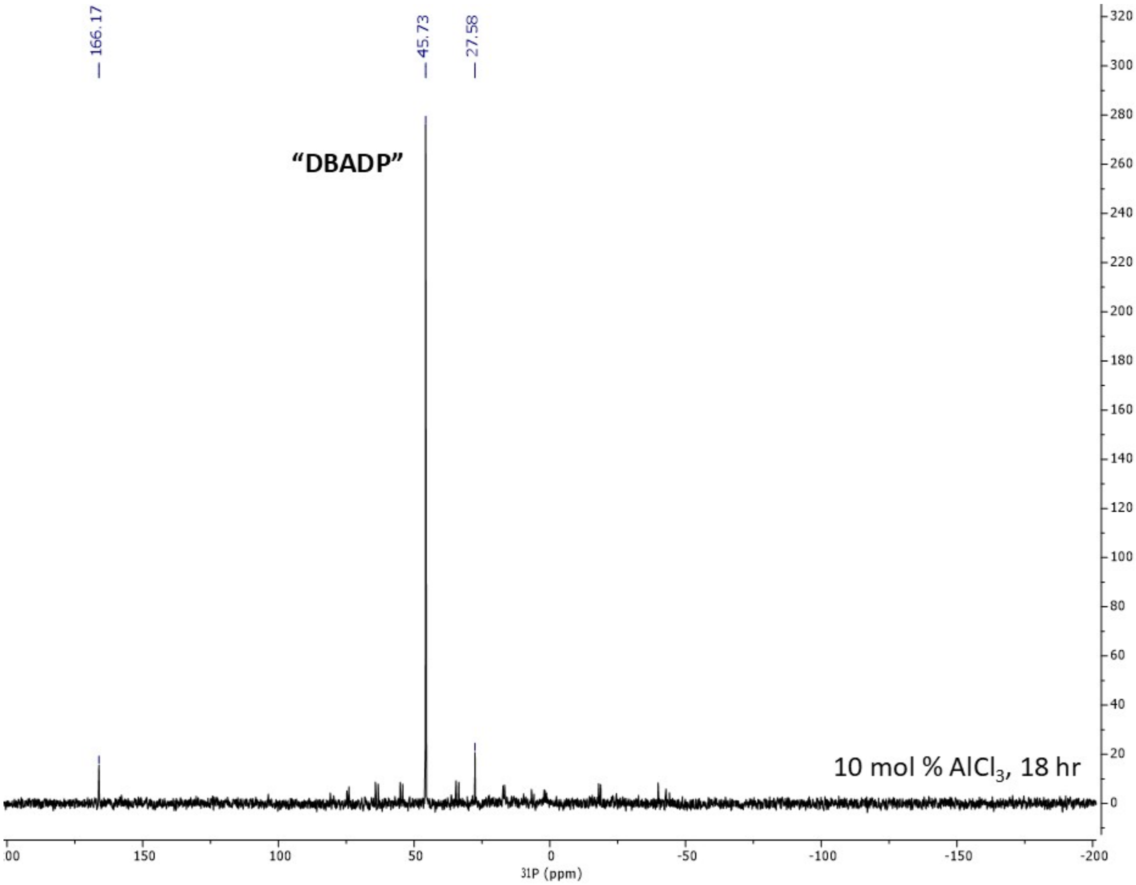

Figure S10: $^{31}P\{^{1}H\}$ NMR Spectra of the reaction of **PAN** (2 eq) with **tBuBQ** (3 eq) and 10 mol % $AlCl_3$ (DCM, 202 MHz, 298 K)

1.5 Reactions of Secondary and Primary Phosphines

a. Diphenyl Phosphine (Ph_2PH)

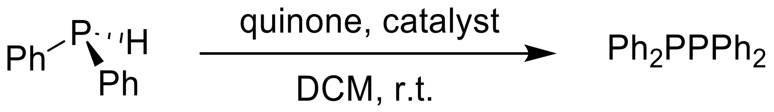

General procedure for Dehydrogenative Coupling of **Ph_2PH** with Lewis/Brønsted acid catalysts

In a glovebox, **Ph_2PH** (0.6 mmol) and anhydrous DCM (4 mL) was added to a 15 mL pressure tube with Teflon screw-top cap. A solution of **tBuBQ** (0.3 mmol or 0.9 mmol) in anhydrous DCM was then added. The reaction mixture was transferred to a glovebag where the corresponding acid catalyst (Triflic Acid, HBF_4•Et_2O, HCl (in Et_2O), or $AlCl_3$) was added. The reaction mixture was then left to stir for a minimum of 10 minutes (**Tables S4 – S5**) before an aliquot was taken for $^{31}P\{^1H\}$ NMR spectroscopy.

Table S4: Reaction conditions for **Ph_2PPPh_2** formation (stoichiometric **tBuBQ**)

Entries	Ph_2PH: tBuBQ	Catalyst	Catalyst Amount	Conditions	Reaction Time	Conversion of Ph_2PH	Yield of Ph_2PPPh_2*
1	2:1	Triflic Acid	1 mol %	DCM, rt	10 min	91 %	81 %
2	2:1	HBF_4•Et_2O	1 mol %	DCM, rt	45 min	35 %	22 %
3	2:1	HCl (in Et_2O)	5 mol %	DCM, rt	30 min	52 %	42 %
4	2:1	$AlCl_3$	10 mol %	DCM, rt	2 h	59 %	58%

* yield was determined by the integration of $^{31}P\{^1H\}$ spectra.

Table S5: Reaction conditions for **Ph_2PPPh_2** formation (excess **tBuBQ**)

Entries	Ph_2PH: tBuBQ	Catalyst	Catalyst Amount	Conditions	Reaction Time	Conversion of Ph_2PH	Yield of Ph_2PPPh_2*
1	2:3	Triflic Acid	1 mol %	DCM, rt	10 min	100 %	88 %
2	2:3	HBF_4•Et_2O	1 mol %	DCM, rt	10 min	69 %	62 %
3	2:3	HCl (in Et_2O)	5 mol %	DCM, rt	30 min	90 %	72 %
4	2:3	$AlCl_3$	10 mol %	DCM, rt	10 min	94 %	90 %

* yield was determined by the integration of $^{31}P\{^1H\}$ spectra.

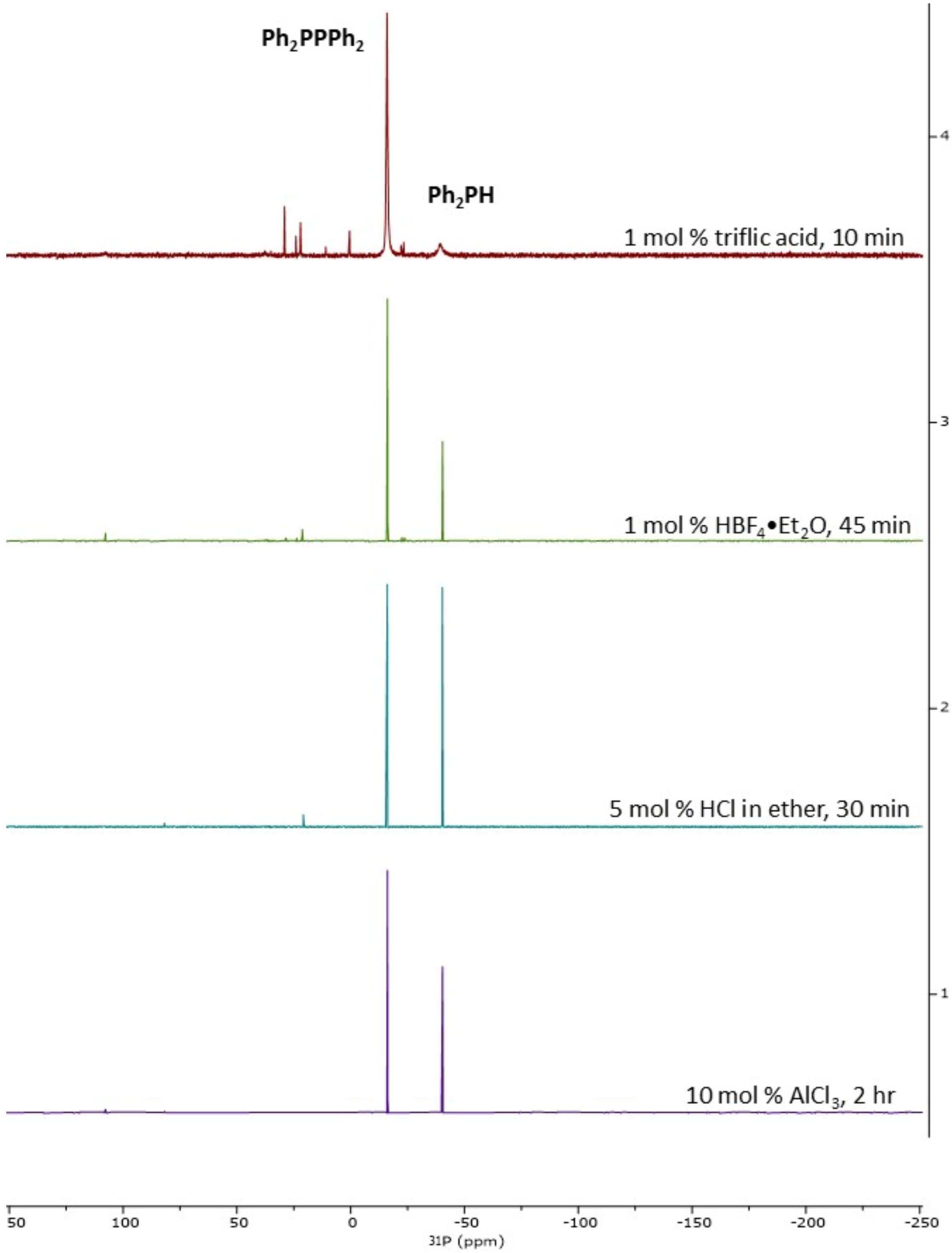

Figure S11: $^{31}P\{^{1}H\}$ NMR Spectra of the reaction of **Ph_2PH** (2 eq) with **tBuBQ** (1 eq) and acid catalysts (DCM, 202 MHz, 298 K).

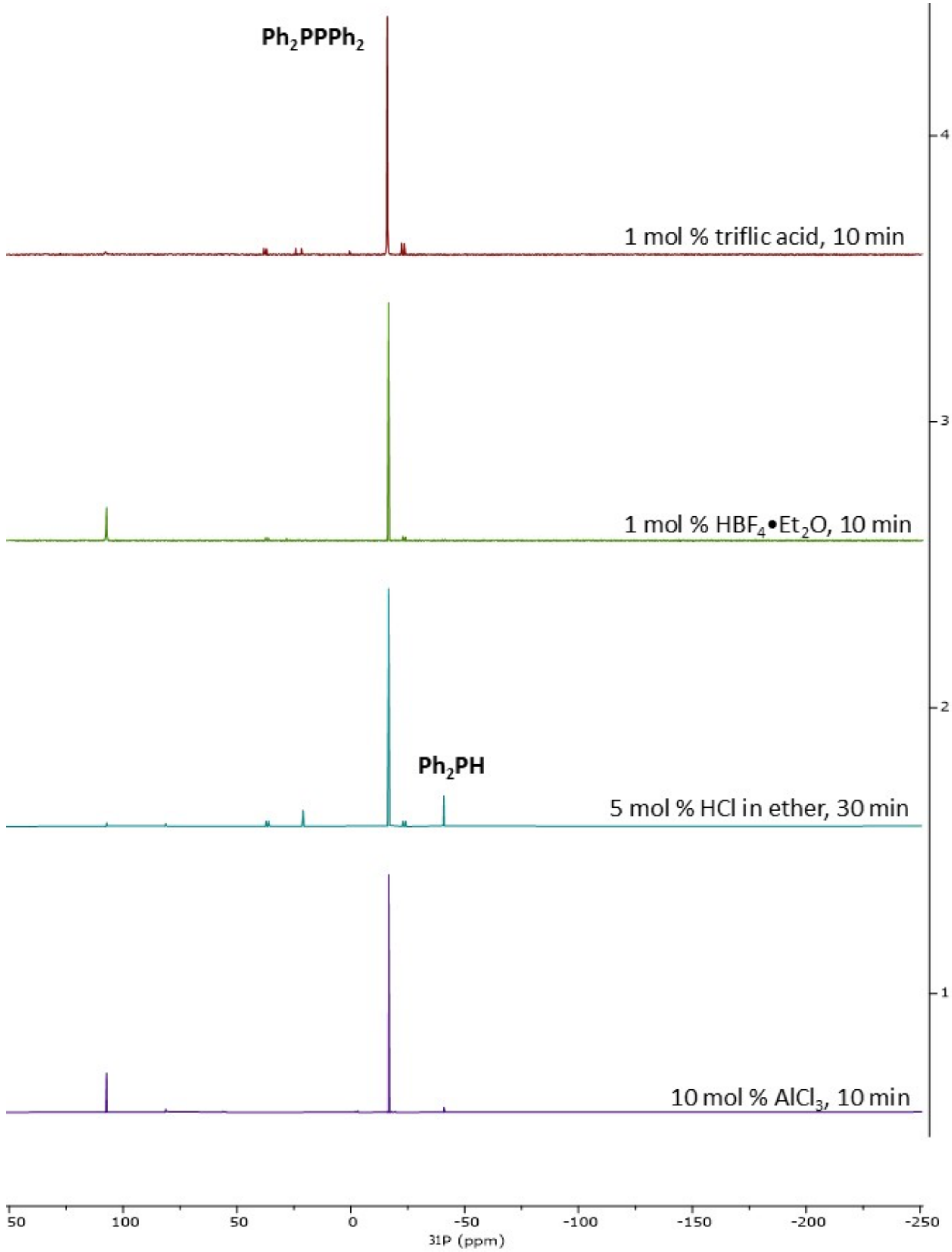

Figure S12: $^{31}P\{^1H\}$ NMR Spectra of the reaction of **Ph_2PH** (2 eq) with **tBuBQ** (3 eq) and acid catalysts (DCM, 202 MHz, 298 K).

b. Attempted Cross-coupling of Diphenyl Phosphine (Ph_2PH) and Dicyclohexyl Phosphine (Cy_2PH)

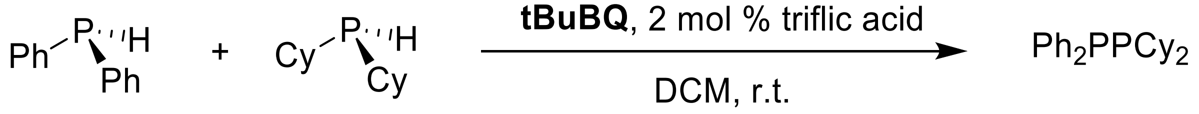

General procedure for cross-coupling of **Ph_2PH** and **Cy_2PH** with triflic acid

In a glovebox, **Ph_2PH** (0.32 mmol), **Cy_2PH** (0.32 mmol), and anhydrous DCM (4 mL) was added to a 15 mL pressure tube with Teflon screw-top cap. A solution of **tBuBQ** (0.48 mmol) in anhydrous DCM was then added. The reaction mixture was transferred to a glovebag where 2 mol % triflic acid was added. The reaction mixture was then monitored over 1.5 weeks via $^{31}P\{^{1}H\}$ NMR spectroscopy.

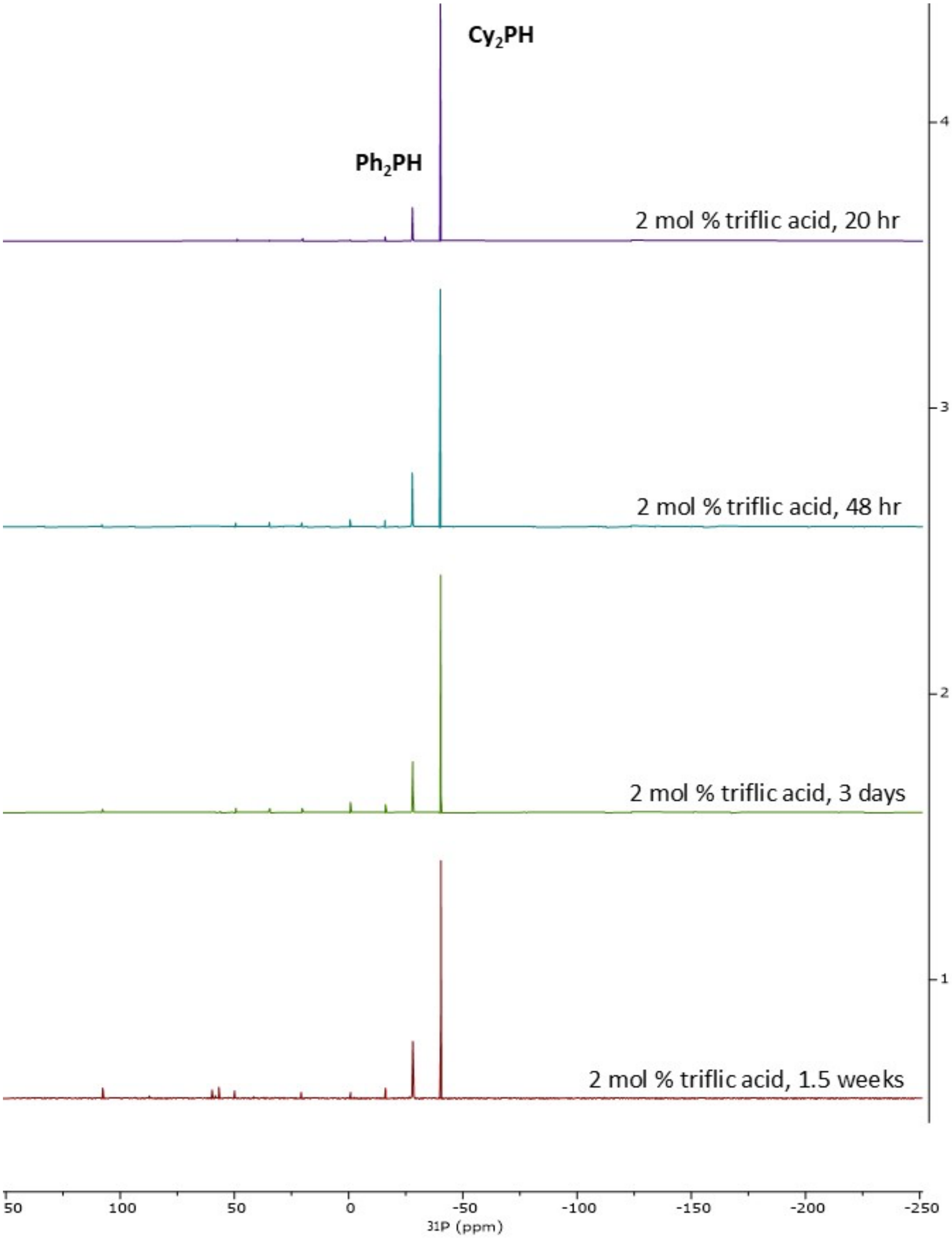

Figure S13: $^{31}P\{^1H\}$ NMR Spectra of the attempted reaction of **Ph_2PH** (1 eq) and **Cy_2PH** (1 eq) with **tBuBQ** (2 eq) and acid catalysts (DCM, 202 MHz, 298 K).

c. Phenyl Phosphine ($PhPH_2$)

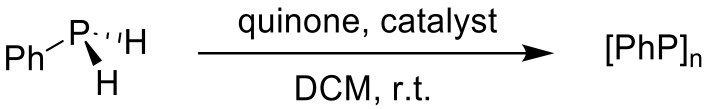

General procedure for Dehydrogenative Coupling of **$PhPH_2$** with Lewis/Brønsted acid catalysts

In a glovebox, **$PhPH_2$** (0.5 mmol) and anhydrous DCM (4 mL) was added to a 15 mL pressure tube with Teflon screw-top cap. A solution of **tBuBQ** (0.5 mmol or 0.75 mmol) in anhydrous DCM (4 mL) was then added. The reaction mixture was transferred to a glovebag where the corresponding acid catalyst (triflic Acid, HBF_4•Et_2O, HCl (in Et_2O), or $AlCl_3$) was added. The reaction mixture was then left to stir for a minimum of 5 minutes (**Tables S6 – S7**) before an aliquot was taken for $^{31}P\{^1H\}$ NMR spectroscopy.

Table S6: Reaction conditions for **$[PhP]_n$** formation (stoichiometric **tBuBQ**)

Entries	$PhPH_2$: tBuBQ	Catalyst	Catalyst Amount	Conditions	Reaction Time	Conversion of $PhPH_2$	Yield of $[PhP]_n$*
1	1:1	Triflic Acid	1 mol %	DCM, rt	10 min	99 %	3% $[PhP]_4$<br>93% $[PhP]_5$<br>2% $[PhP]_6$
2	1:1	HBF_4•Et_2O	3 mol %	DCM, rt	3.5 h	91 %	3% $[PhP]_4$<br>75% $[PhP]_5$<br>1% $[PhP]_6$
3	1:1	HCl (in Et_2O)	2 mol %	DCM, rt	3.5 h	87 %	3% $[PhP]_4$<br>79% $[PhP]_5$<br>1% $[PhP]_6$
4	1:1	$AlCl_3$	10 mol %	DCM, rt	10 min	99 %	4% $[PhP]_4$<br>94% $[PhP]_5$<br>1% $[PhP]_6$

* yield was determined by the integration of $^{31}P\{^1H\}$ spectra.

Table S7: Reaction conditions for **$[PhP]_n$** formation (excess **tBuBQ**)

Entries	$PhPH_2$: tBuBQ	Catalyst	Catalyst Amount	Conditions	Reaction Time	Conversion of $PhPH_2$	Yield of $[PhP]_n$*
1	1:1.5	Triflic Acid	1 mol %	DCM, rt	10 min	100 %	8% $[PhP]_4$<br>88% $[PhP]_5$<br>1% $[PhP]_6$
2	1:1.5	HBF_4•Et_2O	3 mol %	DCM, rt	10 min	100 %	6% $[PhP]_4$<br>88% $[PhP]_5$<br>1% $[PhP]_6$
3	1:1.5	HCl (in Et_2O)	2 mol %	DCM, rt	10 min	100 %	4% $[PhP]_4$<br>94% $[PhP]_5$<br>1% $[PhP]_6$
4	1:1.5	$AlCl_3$	10 mol %	DCM, rt	5 min	99 %	4% $[PhP]_4$<br>93% $[PhP]_5$<br>2% $[PhP]_6$

***** yield was determined by the integration of $^{31}P\{^1H\}$ spectra.

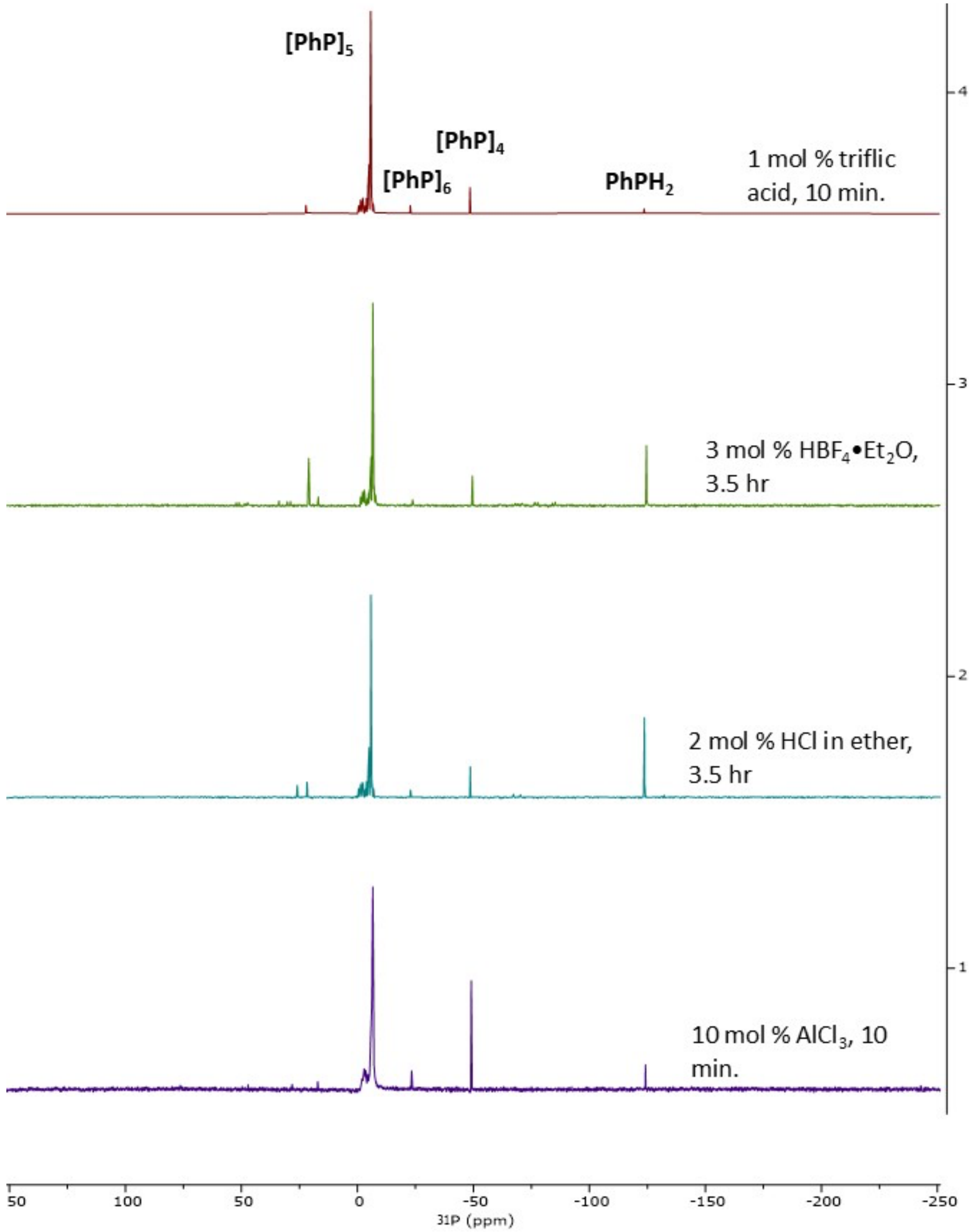

Figure S14: $^{31}P\{^1H\}$ NMR Spectra of the reaction of **$PhPH_2$** (1 eq) with **tBuBQ** (1 eq) and acid catalysts (DCM, 202 MHz, 298 K).

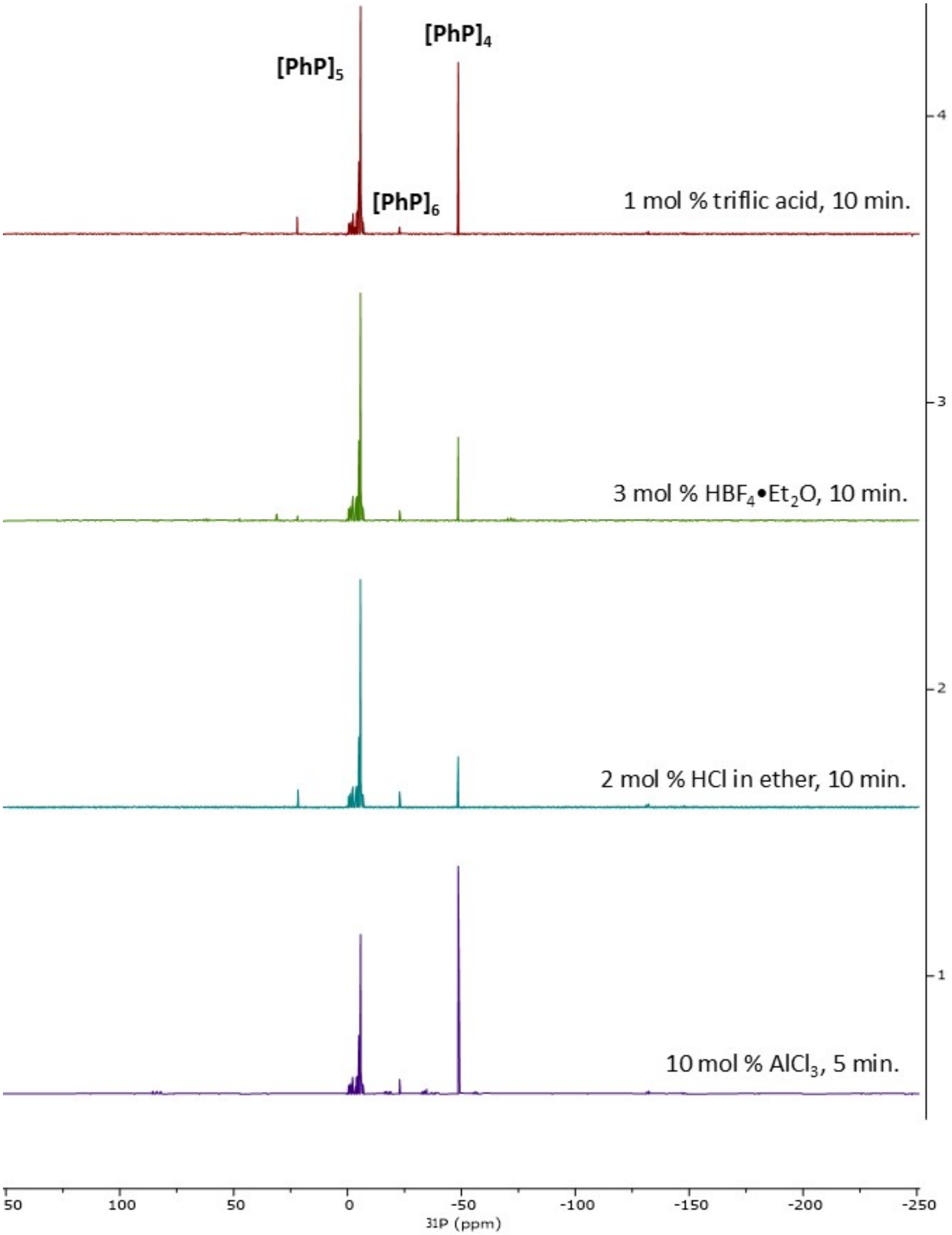

Figure S15: $^{31}P\{^{1}H\}$ NMR Spectra of the reaction of **PhPH₂** (1 eq) with **tBuBQ** (1.5 eq) and acid catalysts (DCM, 202 MHz, 298 K).

d. Cyclohexyl Phosphine ($CyPH_2$)

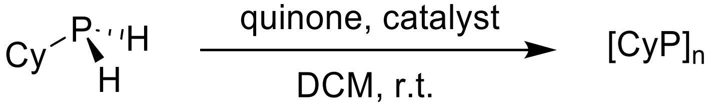

Scheme S9: General reaction for cyclohexylphosphine oligomers (**$[CyP]_n$**) formation from **$CyPH_2$** using an acid catalyst

General procedure for Dehydrogenative Coupling of **$CyPH_2$** with Lewis/Brønsted acid catalysts

In a glovebox, **$CyPH_2$** (0.5 mmol) and anhydrous DCM (4 mL) was added to a 15 mL pressure tube with Teflon screw-top cap. A solution of **tBuBQ** (0.5 mmol or 1.5 mmol) in anhydrous DCM (4 mL) was then added. The reaction mixture was transferred to a glovebag where the corresponding acid catalyst (Triflic Acid, HBF_4•Et_2O, HCl (in Et_2O), or $AlCl_3$) was added. The reaction mixture was then left to stir for a minimum of 10 minutes (**Tables S8 – S9**) before an aliquot was taken for $^{31}P\{^1H\}$ NMR spectroscopy.

Table S8: Reaction conditions for **$[CyP]_n$** formation (stoichiometric **tBuBQ**)

Entries	$CyPH_2$: tBuBQ	Catalyst	Catalyst Amount	Conditions	Reaction Time	Conversion of $CyPH_2$	Yield of $[CyP]_n$*
1	1:1	Triflic Acid	1 mol %	DCM, rt	30 min	41 %	22% $[CyP]_4$
2	1:1	HBF_4•Et_2O	1 mol %	DCM, rt	2 h	24 %	17% $[CyP]_4$
3	1:1	HCl (in Et_2O)	5 mol %	DCM, rt	2 h	22 %	12% $[CyP]_4$
4	1:1	$AlCl_3$	10 mol %	DCM, rt	2 h	25 %	25% $[CyP]_4$

***** yield was determined by the integration of $^{31}P\{^1H\}$ spectra.

Table S9: Reaction conditions for **$[CyP]_n$** formation (excess **tBuBQ**)

Entries	$CyPH_2$: tBuBQ	Catalyst	Catalyst Amount	Conditions	Reaction Time	Conversion of $CyPH_2$	Yield of $[CyP]_n$*
1	1:3	Triflic Acid	1 mol %	DCM, rt	10 min	100 %	29% $[CyP]_4$<br>24% $[CyP]_5$
2	1:3	HBF_4•Et_2O	1 mol %	DCM, rt	10 min	84 %	30% $[CyP]_4$<br>50% $[CyP]_5$
3	1:3	HCl (in Et_2O)	5 mol %	DCM, rt	10 min	100 %	75% $[CyP]_4$
4	1:3	$AlCl_3$	10 mol %	DCM, rt	10 min	100 %	100% $[CyP]_4$

***** yield was determined by the integration of $^{31}P\{^1H\}$ spectra.

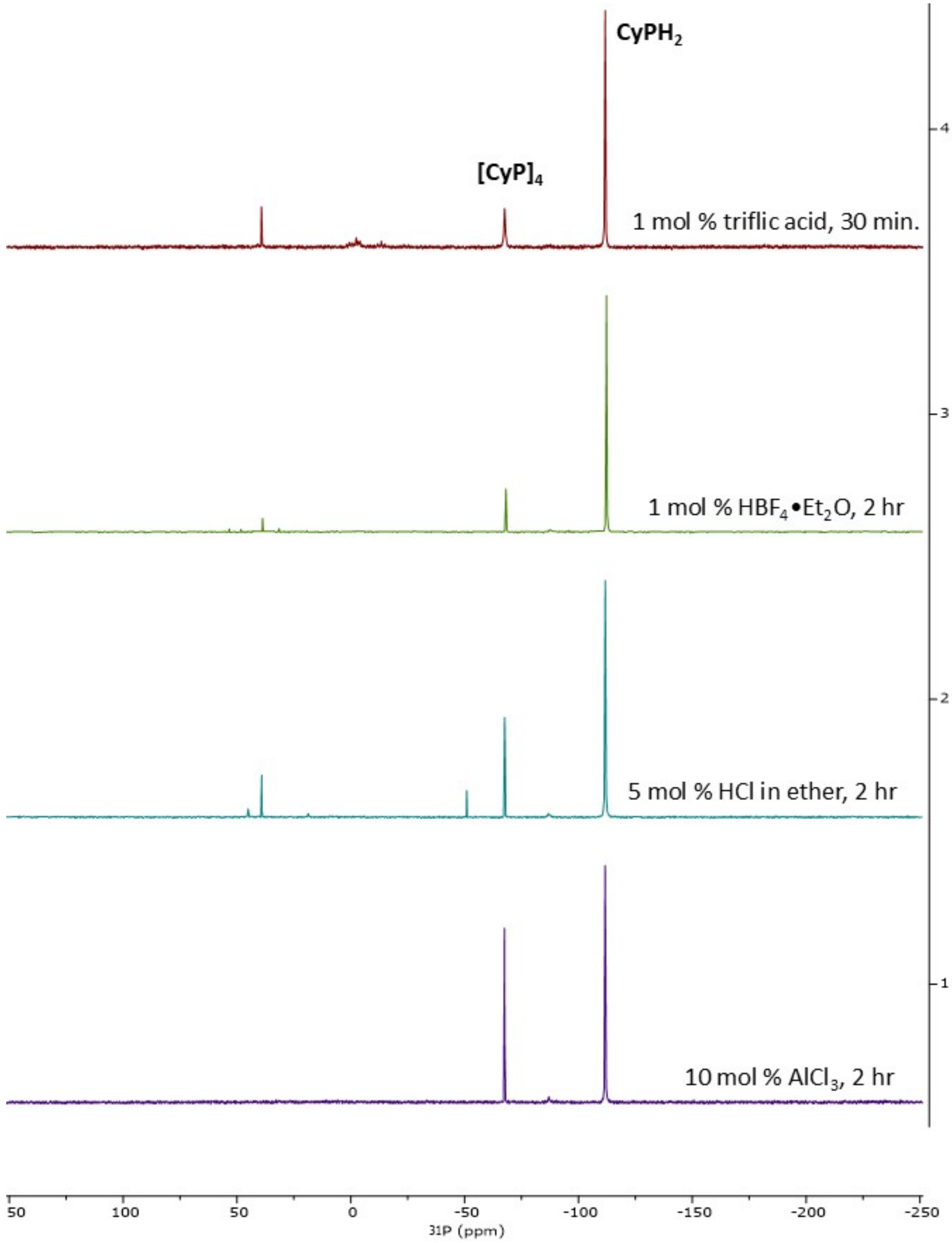

Figure S16: $^{31}P\{^{1}H\}$ NMR Spectra of the reaction of **CyPH₂** (1 eq) with **tBuBQ** (1 eq) and acid catalysts (DCM, 202 MHz, 298 K).

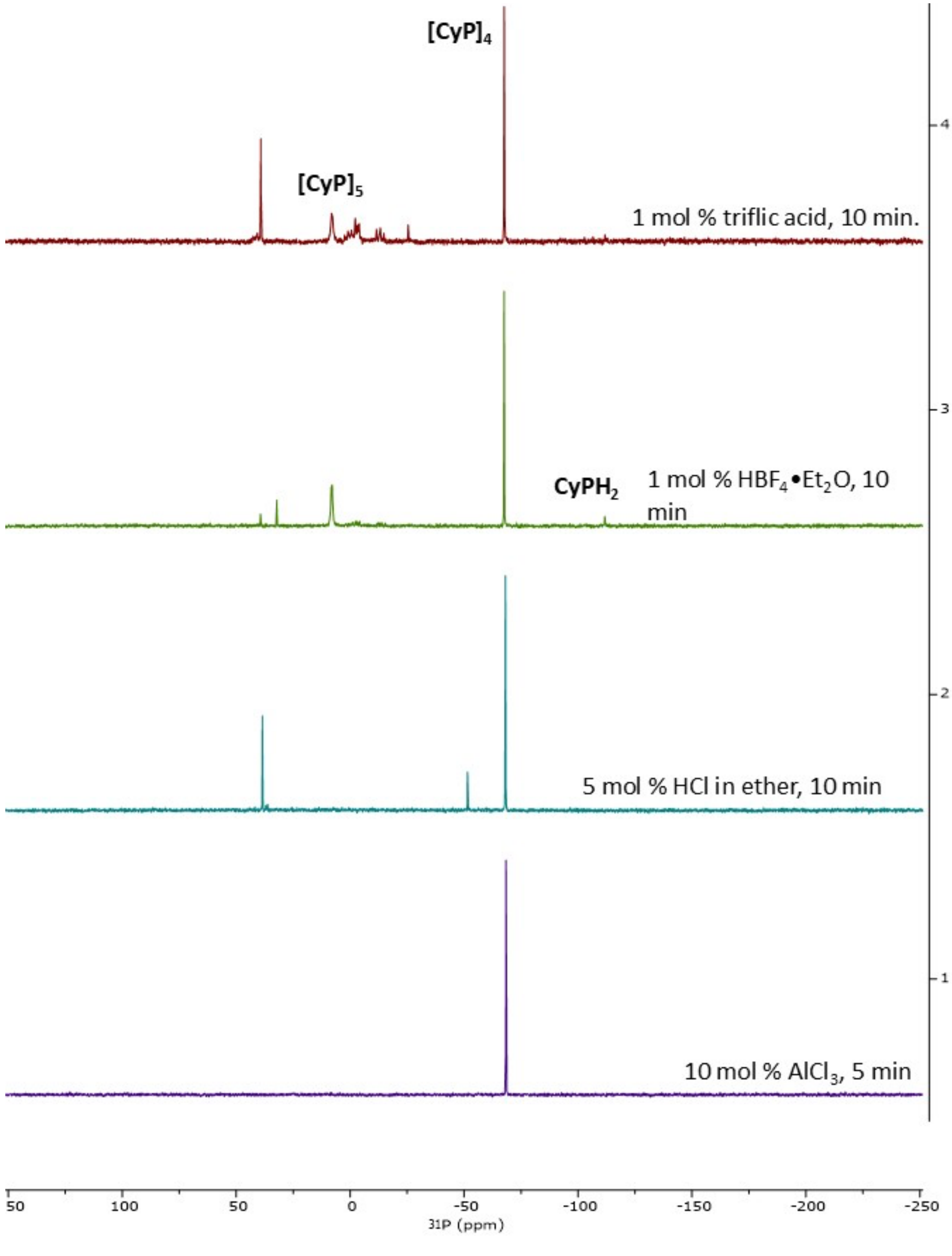

Figure S17: $^{31}P\{^{1}H\}$ NMR Spectra of the reaction of **CyPH₂** (1 eq) with **tBuBQ** (3 eq) and acid catalysts (DCM, 202 MHz, 298 K).

Table S10: Global summary of selected results for dehydrocoupling reactions, yields and conversions estimated by $^{31}P\{^1H\}$ NMR spectroscopy. Data provided for reactions using stoichiometric (left) or excess (right) amounts of **tBuBQ** in CH_2Cl_2 solvent. Reaction conditions in *italics*.

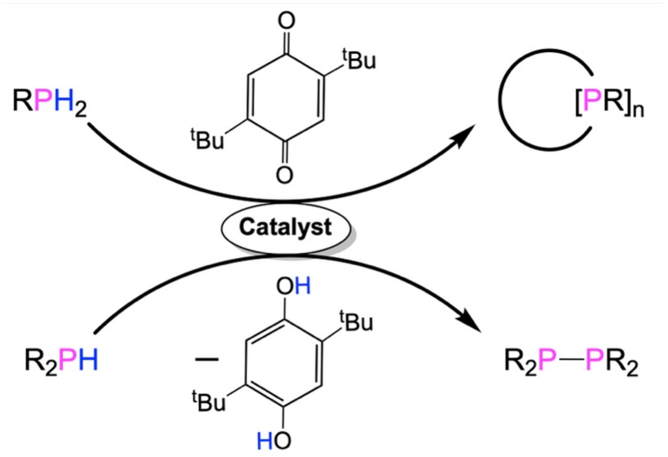

	Ph_2PH:tBuBQ		$PhPH_2$:tBuBQ		$CyPH_2$:tBuBQ	
**Catalyst**	2:1	2:X	1:1	1:X	1:1	1:X
**Triflic Acid**	81% Ph_2PPPh_2<br>*1 %,[a] 10 min,[b] {91%}[c]*	X = 3<br>88% Ph_2PPPh_2<br>*1 %, 10 min, {100%}*	3% $[PhP]_4$<br>93% $[PhP]_5$<br>2% $[PhP]_6$<br>*1 %, 10 min, {99%}*	X = 1.5<br>8% $[PhP]_4$<br>88% $[PhP]_5$<br>1% $[PhP]_6$<br>*1 %, 10 min, {100%}*	22% $[CyP]_4$<br>*1 %, 30 min, {41%}*	X = 3<br>29% $[CyP]_4$<br>24% $[CyP]_5$<br>*1 %, 10 min, {100%}*
**HBF_4•Et_2O**	22% Ph_2PPPh_2<br>*1 %, 45 min, {35%}*	X = 3<br>62% Ph_2PPPh_2<br>*1 %, 10 min, {69%}*	3% $[PhP]_4$<br>75% $[PhP]_5$<br>1% $[PhP]_6$<br>*3 %, 3.5 h, {91%}*	X = 1.5<br>6% $[PhP]_4$<br>88% $[PhP]_5$<br>1% $[PhP]_6$<br>*3 %, 10 min, {100%}*	17% $[CyP]_4$<br>*1 %, 2 h, {24%}*	X = 3<br>30% $[CyP]_4$<br>50% $[CyP]_5$<br>*1 %, 10 min, {84%}*
**HCl•Et_2O**	42% Ph_2PPPh_2<br>*5 %, 30 min, {52%}*	X = 3<br>72% Ph_2PPPh_2<br>*5 %, 30 min, {90%}*	3% $[PhP]_4$<br>79% $[PhP]_5$<br>1% $[PhP]_6$<br>*2 %, 3.5 h, {87%}*	X = 1.5<br>4% $[PhP]_4$<br>94% $[PhP]_5$<br>1% $[PhP]_6$<br>*2 %, 10 min, {100%}*	12% $[CyP]_4$<br>*5 %, 2 h, {22%}*	X = 3<br>75% $[CyP]_4$<br>*5 %, 10 min, {100%}*
**$AlCl_3$**	58% Ph_2PPPh_2<br>*10 %, 2 h, {59%}*	X = 3<br>90% Ph_2PPPh_2<br>*10 %, 10 min, {94%}*	4% $[PhP]_4$<br>94% $[PhP]_5$<br>1% $[PhP]_6$<br>*10 %, 10 min, {99%}*	X = 3<br>4% $[PhP]_4$<br>93% $[PhP]_5$<br>2% $[PhP]_6$<br>*10 %, 5 min, {99%}*	25% $[CyP]_4$<br>*10 %, 2 h, {25%}*	X = 3<br>100% $[CyP]_4$<br>*10 %, 10 min, {100%}*

[a] mol percentage catalyst. [b] reaction time. [c] conversion of primary or secondary phosphine to products

1.6 Alternative hydrogen acceptors

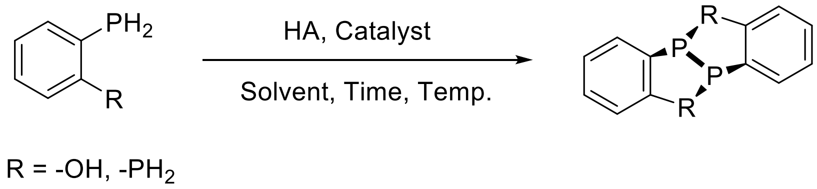

a. Dehydrogenative Coupling of **PP** with azobenzene (**AZB**)

In a glovebox, a dry vial was charged with **PP** (0.40 mmol) and anhydrous DCM (1 mL). The vial was brought out of the glove box and into a glove bag. A separate vial was charged with **AZB** (0.60 mmol) and DCM (0.5 mL) before being added to the reaction flask inside a glove bag. Then triflic acid (0.004 mmol) was added via syringe. The solution was transferred to an NMR tube and the reaction was monitored via $^{31}P\{^{1}H\}$ NMR spectroscopy where after 13 hours all starting material had been reacted.

b. Dehydrogenative Coupling of **PP** with **DQI**

In a glovebox, a dry vial was charged with **PP** (0.40 mmol) and anhydrous DCM (1 mL). The vial was brought out of the glove box and into a glove bag. A separate vial was charged with a **DQI** (0.60 mmol) and DCM (0.5 mL) before being added to the reaction flask. Then triflic acid (0.004 mmol) was added via syringe. The solution was transferred to an NMR tube and the reaction was monitored via $^{31}P\{^{1}H\}$ NMR spectroscopy where after 10 minutes all starting material had been reacted.

c. Dehydrogenative Coupling of **DPB** with **DQI** (**no catalyst**)

In a glovebox, a dry vial was charged with **DPB** (0.35 mmol) and anhydrous DCM (1 mL). The vial was brought out of the glove box and into a glove bag. A separate vial was charged with a **DQI** (0.53 mmol) and DCM (0.5 mL) before being added to the reaction flask. The solution was transferred to an NMR tube and the reaction was monitored via $^{31}P\{^{1}H\}$ NMR spectroscopy over 24 hours.

d. Dehydrogenative Coupling of **DPB** with **DQI** (**with catalyst**)

In a glovebox, a dry vial was charged with **DPB** (0.35 mmol) and anhydrous DCM (1 mL). The vial was brought out of the glove box and into a glove bag. A separate vial was charged with a **DQI** (0.53 mmol) and DCM (0.5 mL) before being added to the reaction flask. Then triflic acid (0.004 mmol) was added via syringe. The solution was transferred to an NMR tube and the reaction was monitored via $^{31}P\{^{1}H\}$ NMR spectroscopy over 24 hours.

Table S11: Reaction conditions for dimer formation with different **HA**

Entries	PP/DPB: HA	HA	Catalyst	Catalyst Amount	Conditions	Reaction Time	Conversion of Starting Material	Yield of Dimer
1	2:3	**AZB**	Triflic Acid	1 mol %	DCM, rt	13 h	100 %	100 %
2	2:3	**DQI**	--	--	DCM, rt	10 min	100 %	96 %
3	2:3	**DQI**	--	--	DCM, rt	24 h	43 %	14 %
4	2:3	**DQI**	Triflic Acid	1 mol %	DCM, rt	24 h	75 %	66 %

***** yield was determined by the integration of $^{31}P\{^{1}H\}$ spectra.

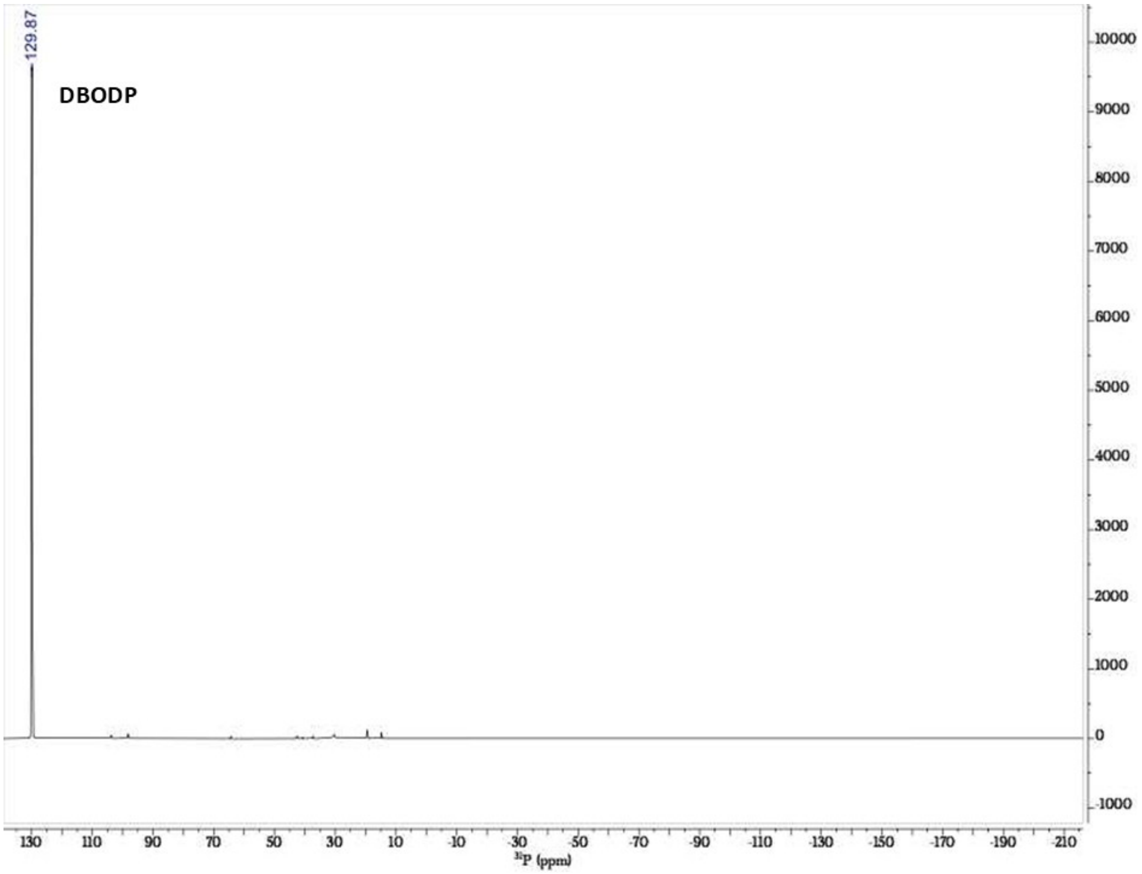

Figure S18: $^{31}P\{^{1}H\}$ NMR Spectrum of the reaction of **PP** (2 eq) with **AZB** (3 eq) and 1 mol % triflic acid (DCM, 202 MHz, 298 K).

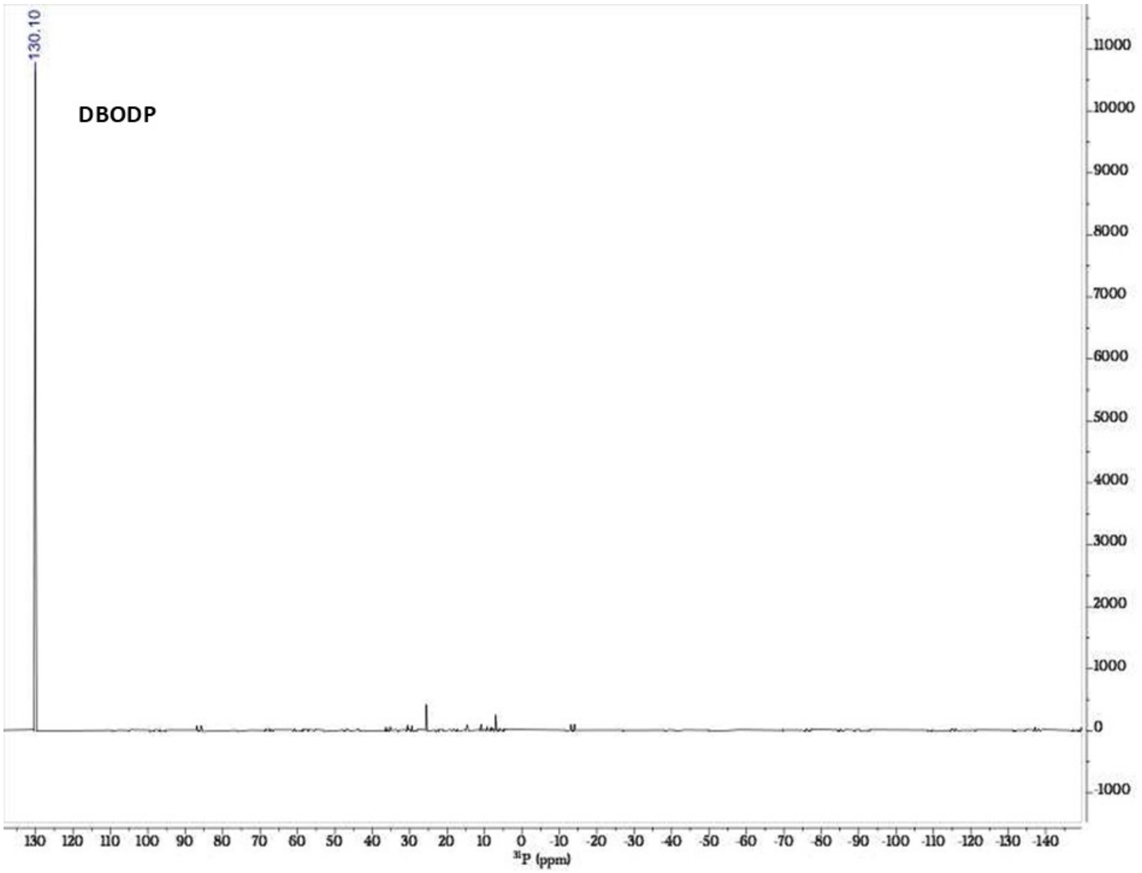

Figure S19: $^{31}P\{^{1}H\}$ NMR Spectrum of the reaction of **PP** (2 eq) with **DQI** (3 eq) and no catalyst (DCM, 202 MHz, 298 K).

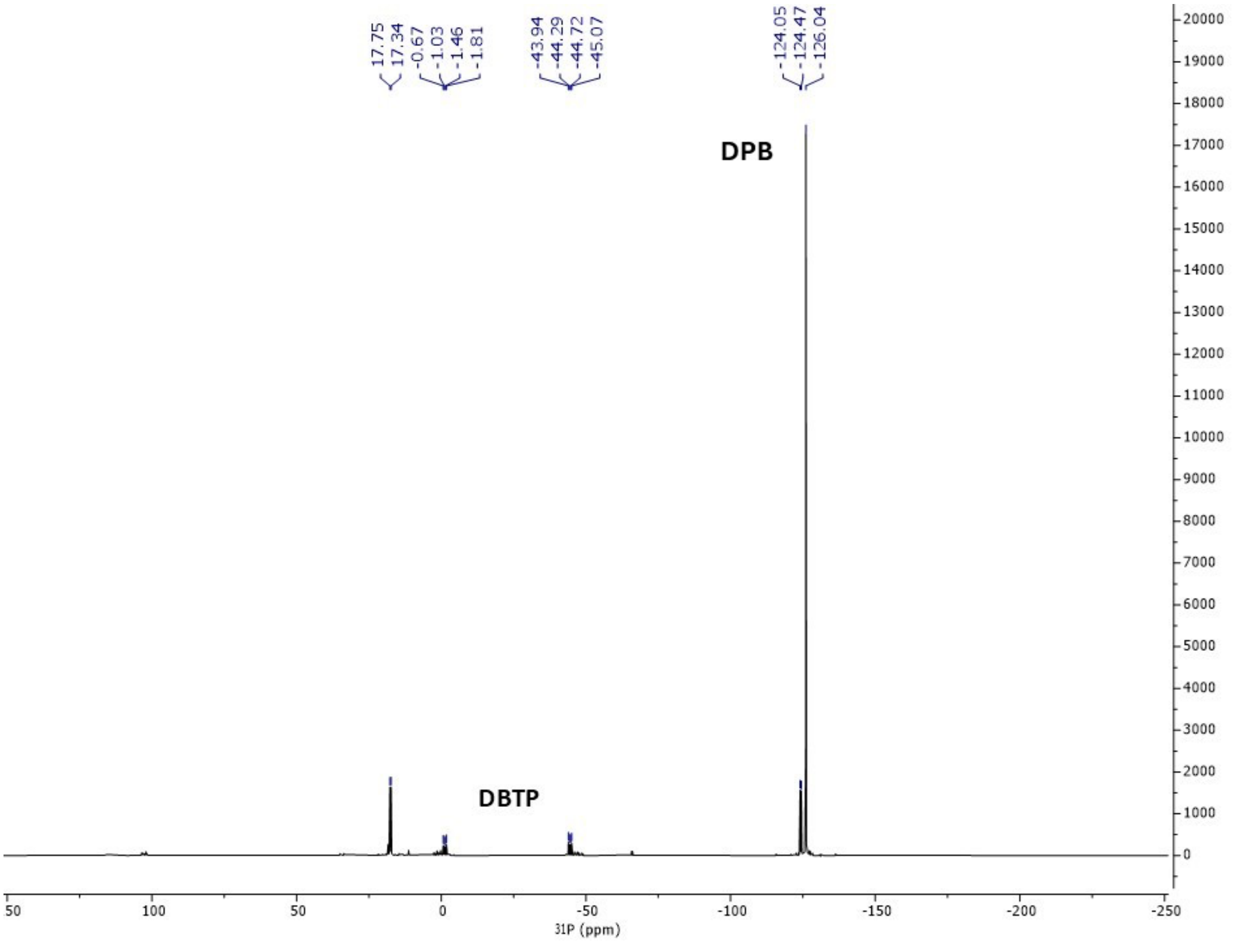

Figure S20: $^{31}P\{^{1}H\}$ NMR Spectrum of the reaction of **DPB** (2 eq) with **DQI** (3 eq) and no catalyst (DCM, 202 MHz, 298 K).

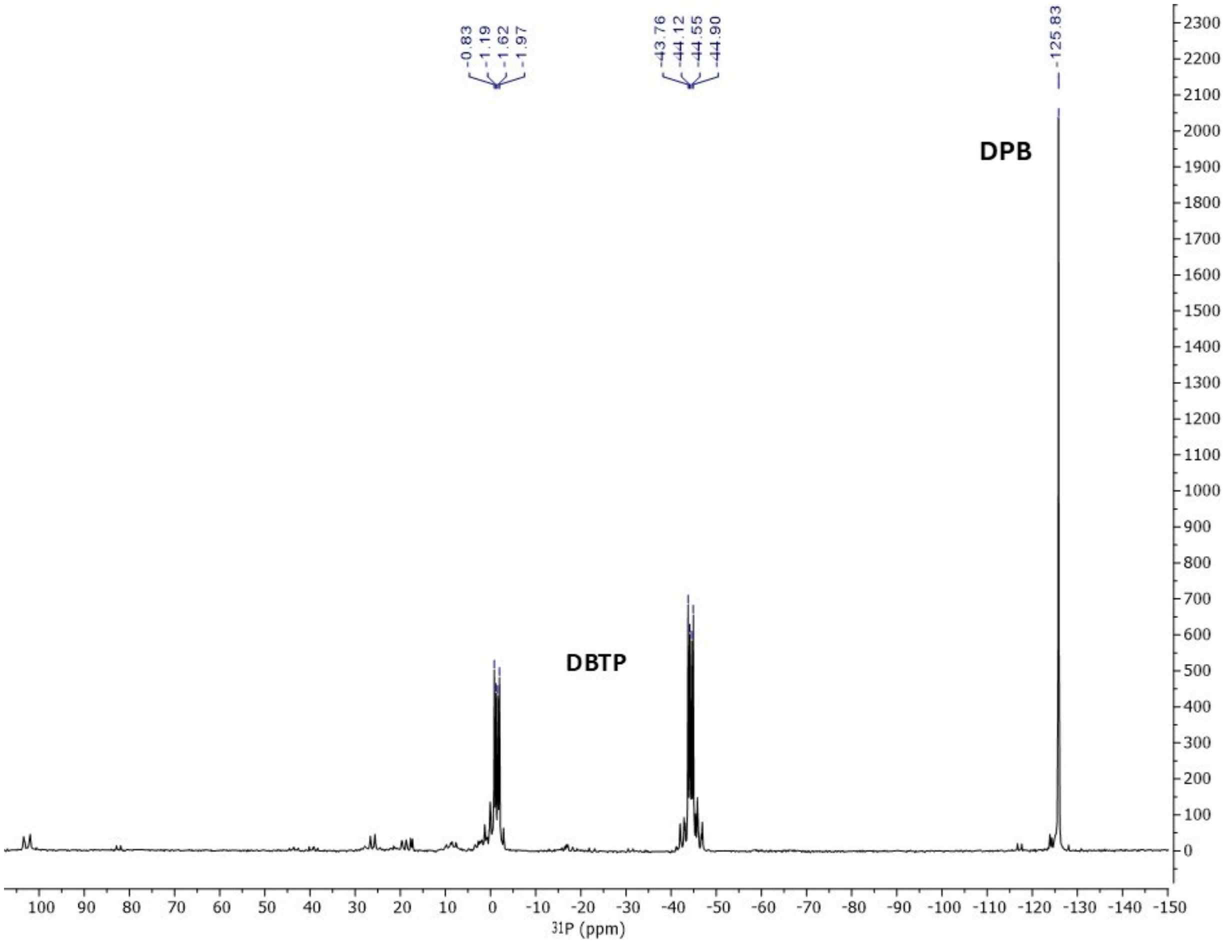

Figure S21: $^{31}P\{^{1}H\}$ NMR Spectrum of the reaction of **DPB** (2 eq) with **DQI** (3 eq) and 1 mol % triflic acid (DCM, 202 MHz, 298 K).

2. X-ray Crystal Structure Analyses

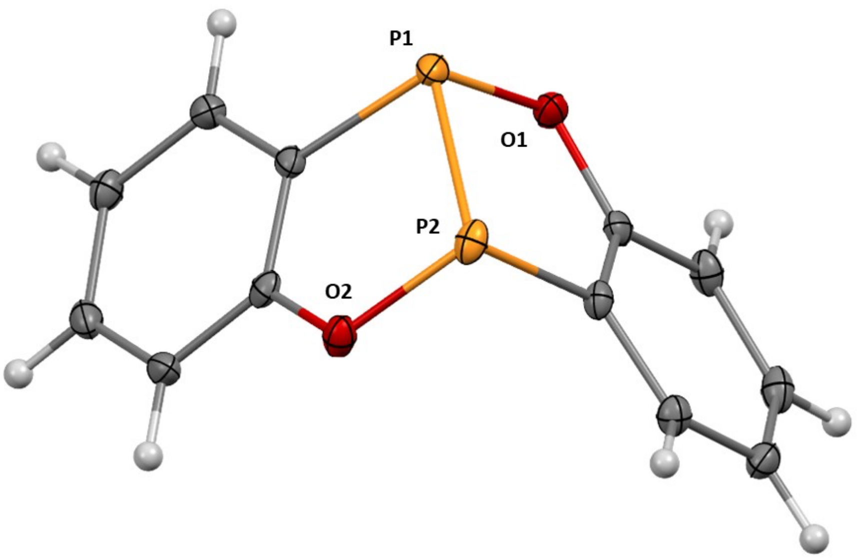

Figure S22: Crystal structure of **DBODP**

Table S12: Crystal structure and data refinement for **DBODP**

Identification code	pro217_a
Empirical formula	$C_{12} H_8 O_2 P_2$
Formula weight	246.12
Temperature	100 K
Wavelength	0.71073 Å
Crystal system	Monoclinic
Space group	$C2/c$ (15)
Unit cell dimensions	a = 8.2939(3) Å
	b = 12.7278(4) Å
	c = 9.7046(3) Å
Volume	1024.42(6) Å^3
Z	4
Density (calculated)	1.596 g/cm^3
Absorption coefficient	0.401 mm^{-1}
F(000)	504
Crystal color, size	colourless, 0.23 x 0.27 x 0.29 mm^3
Theta range for data collection	4.86 to 54.20 Å
Index ranges	-10<=h<=10, -16<=k<=16, -12<=l<=12
Reflections collected	5621
Independent reflections	1136 [R(int) = 0.0417]
Completeness to theta = 25.242°	100.0 %

Absorption correction	0.4477/0.4912
Max. and min. transmission	-
Refinement method	Full-matrix least-squares on F^2
Data / restraints / parameters	1136 / 0 / 73
Goodness-of-fit on F^2	1.092
Final R indices [I>2sigma(I)]	R1 = 0.0303, wR2 = 0.0809
R indices (all data)	R1 = 0.0332, wR2 = 0.0842
Extinction coefficient	-
Largest diff. peak and hole	0.40 and -0.18 e.Å^{-3}

Table S13: Selected Bond Lengths (Å) for **DBODP**

Atom	Atom	Length/Å	Atom	Atom	Length/Å
P1	P1	2.2499(8)	C3	H3	0.9500
P1	O1	1.6916(11)	C3	C4	1.388(2)
P1	C2	1.8195(14)	C6	H6	0.9500
O1	C1	1.3753(17)	C6	C5	1.385(2)
C2	C1	1.392(2)	C4	H4	0.9500
C2	C3	1.400(2)	C4	C5	1.392(2)
C1	C6	1.391(2)	C5	H5	0.9500

Table S14: Selected Bond Angles (°) for **DBODP**

Atom	Atom	Atom	Angle/˚	Atom	Atom	Atom	Angle/˚
O1	P1	P1	96.48(4)	C4	C3	C2	119.96(13)
O1	P1	C2	101.00(6)	C4	C3	H3	120.0
C2	P1	P1	88.27(5)	C1	C6	H6	120.6
C1	O1	P1	117.02(9)	C5	C6	C1	118.85(14)
C1	C2	P1	118.32(11)	C5	C6	H6	120.6
C1	C2	C3	119.11(13)	C3	C4	H4	120.1
C3	C2	P1	122.55(11)	C3	C4	C5	119.88(13)
O1	C1	C2	119.53(13)	C5	C4	H4	120.1
O1	C1	C6	119.23(13)	C6	C5	C4	120.88(14)
C6	C1	C2	121.24(13)	C6	C5	H5	119.6
C2	C3	H3	120.0	C4	C5	H5	119.6

Table S15: Fractional Atomic Coordinates and Equivalent Isotropic Displacement Parameters (Å^2) for **DBODP**. U_{eq} is defined as 1/3 of the trace of the orthogonalized U_{IJ} tensor.

Atom	*x*	*y*	*z*	U_{eq}
P1	0.57709(4)	0.78317(3)	0.15512(4)	0.01477(16)
O1	0.72018(12)	0.70047(9)	0.21659(11)	0.0159(3)
C2	0.56420(17)	0.69067(11)	0.42232(14)	0.0122(3)
C1	0.69404(17)	0.65667(11)	0.34405(14)	0.0126(3)
C3	0.54469(17)	0.65069(12)	0.55550(15)	0.0143(3)
H3	0.459381	0.675489	0.611594	0.017
C6	0.79971(17)	0.58030(12)	0.39316(15)	0.0155(3)
H6	0.887005	0.556839	0.338319	0.019
C4	0.65010(18)	0.57477(12)	0.60559(15)	0.0157(3)
H4	0.636641	0.547262	0.695774	0.019
C5	0.77548(17)	0.53903(12)	0.52344(15)	0.0166(3)
H5	0.845390	0.485624	0.557163	0.020

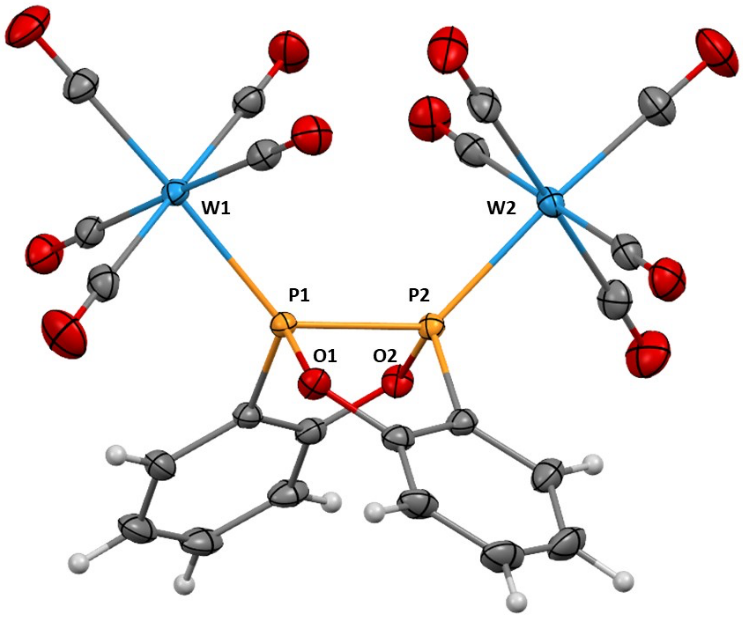

Figure S23: Crystal structure of **[W(CO)$_5$]$_2$DBODP**

Table S16: Crystal structure and data refinement for **[W(CO)$_5$]$_2$DBODP**

Identification code	hh_1_96_1_0m
Empirical formula	$C_{22} H_8 O_{12} P_4 W_2$
Formula weight	893.92
Temperature	150 K
Wavelength	0.71073 Å
Crystal system	Triclinic
Space group	$P\overline{1}$ (2)
Unit cell dimensions	a = 9.2136(4) Å
	b = 10.4905(5) Å
	c = 15.2512(7) Å
Volume	1285.71(14) Å^3
Z	2
Density (calculated)	2.309 g/cm^3
Absorption coefficient	9.126 mm^{-1}
F(000)	828
Crystal color, size	yellow, 0.420 x 0.220 x 0.120 mm^3
Theta range for data collection	4.82 to 66.41 Å
Index ranges	-14<=h<=14, -16<=k<=16, -22<=l<=23
Reflections collected	37562
Independent reflections	9751 [R(int) = 0.0384]
Completeness to theta = 25.242°	99.5 %
Absorption correction	0.3576/0.7465
Max. and min. transmission	0.7455 and 0.4036
Refinement method	Full-matrix least-squares on F^2
Data / restraints / parameters	9751 / 0 / 344
Goodness-of-fit on F^2	1.083
Final R indices [I>2sigma(I)]	R1 = 0.0217, wR2 = 0.0502
R indices (all data)	R1 = 0.0266, wR2 = 0.0516
Extinction coefficient	0.00306(19)
Largest diff. peak and hole	1.54 and -1.79 e.Å^{-3}

Table S17: Selected Bond Lengths (Å) for **[W(CO)$_5$]$_2$DBODP**

Atom	Atom	Length/Å	Atom	Atom	Length/Å
W1	C13	2.028(3)	W2	C21	2.057(3)
W1	C15	2.046(3)	W2	P2	2.4290(6)
W1	C17	2.051(3)	P1	O2	1.6530(18)
W1	C16	2.056(3)	P1	C1	1.807(2)
W1	C14	2.057(3)	P1	P2	2.2410(9)
W1	P1	2.4237(6)	P2	O1	1.6504(17)
W2	C18	2.030(3)	P2	C7	1.808(3)
W2	C19	2.046(3)	O1	C6	1.387(3)
W2	C22	2.048(3)	O2	C12	1.385(3)
W2	C20	2.054(3)	O3	C13	1.141(4)

Atom	Atom	Length/Å
O4	C14	1.131(3)
O5	C15	1.139(4)
O6	C16	1.127(3)
O7	C17	1.135(3)
O8	C18	1.144(4)
O9	C19	1.139(3)
O10	C20	1.135(4)
O11	C21	1.136(3)
O12	C22	1.132(4)
C1	C6	1.390(3)
C1	C2	1.398(3)
C2	C3	1.379(4)
C2	H2	0.9500
C3	C4	1.389(5)
C3	H3	0.9500
C4	C5	1.383(4)
C4	H4	0.9500
C5	C6	1.390(3)
C5	H5	0.9500
C7	C12	1.389(4)
C7	C8	1.401(4)
C8	C9	1.387(4)
C8	H8	0.9500
C9	C10	1.384(5)
C9	H9	0.9500
C10	C11	1.383(5)
C10	H10	0.9500
C11	C12	1.390(3)
C11	H11	0.9500

Table S18: Selected Bond Angles (°) for **W(CO)$_5$]$_2$DBODP**

Atom	Atom	Atom	Angle/˚
C13	W1	C15	90.11(12)
C13	W1	C17	90.41(11)
C15	W1	C17	179.40(11)
C13	W1	C16	91.59(11)
C15	W1	C16	88.81(11)
C17	W1	C16	90.87(10)
C13	W1	C14	90.05(11)
C15	W1	C14	91.55(11)
C17	W1	C14	88.76(11)
C16	W1	C14	178.32(10)
C13	W1	P1	177.14(9)
C15	W1	P1	87.03(8)
C17	W1	P1	92.46(8)
C16	W1	P1	88.44(7)
C14	W1	P1	89.93(7)
C18	W2	C19	89.98(12)
C18	W2	C22	90.22(12)
C19	W2	C22	89.05(11)
C18	W2	C20	90.27(13)
C19	W2	C20	89.83(11)
C22	W2	C20	178.77(11)
C18	W2	C21	90.25(11)
C19	W2	C21	178.05(11)
C22	W2	C21	89.02(11)
C20	W2	C21	92.10(11)
C18	W2	P2	175.36(10)
C19	W2	P2	91.01(8)
C22	W2	P2	94.33(8)
C20	W2	P2	85.19(8)
C21	W2	P2	88.91(7)
O2	P1	C1	105.17(10)
O2	P1	P2	95.40(7)
C1	P1	P2	88.35(9)
O2	P1	W1	112.40(7)
C1	P1	W1	119.67(8)
P2	P1	W1	130.76(3)
O1	P2	C7	104.94(10)
O1	P2	P1	95.28(7)

Atom	Atom	Atom	Angle/˚	Atom	Atom	Atom	Angle/˚
C7	P2	P1	87.94(9)	C8	C7	P2	124.5(2)
O1	P2	W2	112.89(7)	C9	C8	C7	119.9(3)
C7	P2	W2	118.08(8)	C9	C8	H8	120.0
P1	P2	W2	132.48(3)	C7	C8	H8	120.0
C6	O1	P2	116.20(15)	C10	C9	C8	119.8(3)
C12	O2	P1	116.04(15)	C10	C9	H9	120.1
C6	C1	C2	119.0(2)	C8	C9	H9	120.1
C6	C1	P1	116.09(18)	C11	C10	C9	121.5(3)
C2	C1	P1	124.8(2)	C11	C10	H10	119.3
C3	C2	C1	119.9(3)	C9	C10	H10	119.3
C3	C2	H2	120.1	C10	C11	C12	118.1(3)
C1	C2	H2	120.1	C10	C11	H11	121.0
C2	C3	C4	120.0(3)	C12	C11	H11	121.0
C2	C3	H3	120.0	O2	C12	C7	119.3(2)
C4	C3	H3	120.0	O2	C12	C11	118.7(2)
C5	C4	C3	121.4(3)	C7	C12	C11	121.9(3)
C5	C4	H4	119.3	O3	C13	W1	179.4(3)
C3	C4	H4	119.3	O4	C14	W1	178.5(2)
C4	C5	C6	118.0(3)	O5	C15	W1	179.0(3)
C4	C5	H5	121.0	O6	C16	W1	178.8(2)
C6	C5	H5	121.0	O7	C17	W1	179.4(3)
O1	C6	C5	118.6(2)	O8	C18	W2	179.8(4)
O1	C6	C1	119.7(2)	O9	C19	W2	178.2(3)
C5	C6	C1	121.7(2)	O10	C20	W2	178.3(3)
C12	C7	C8	118.7(2)	O11	C21	W2	178.4(2)
C12	C7	P2	116.63(19)	O12	C22	W2	179.2(3)

Table S19: Fractional Atomic Coordinates and Equivalent Isotropic Displacement Parameters (Å^2) for **W(CO)$_5$]$_2$DBODP**. U_{eq} is defined as 1/3 of the trace of the orthogonalized U_{IJ} tensor.

Atom	*x*	*y*	*z*	U_{eq}
W1	0.71325(2)	0.70770(2)	0.88435(2)	0.01602(3)
W2	0.19432(2)	0.94176(2)	0.66164(2)	0.01766(3)
P1	0.64348(7)	0.62603(6)	0.76952(4)	0.01434(10)
P2	0.41996(7)	0.71253(6)	0.68971(4)	0.01468(10)
O1	0.3705(2)	0.57177(19)	0.74056(12)	0.0197(3)
O2	0.7610(2)	0.6314(2)	0.67803(12)	0.0202(3)

Atom	*x*	*y*	*z*	U_{eq}
O3	0.8237(3)	0.8048(3)	1.03275(17)	0.0415(6)
O4	0.6749(3)	0.4397(2)	1.04139(15)	0.0319(4)
O5	1.0722(3)	0.5215(3)	0.8466(2)	0.0440(6)
O6	0.7456(3)	0.9734(2)	0.72298(15)	0.0315(4)
O7	0.3531(3)	0.8944(3)	0.91943(16)	0.0373(5)
O8	-0.1131(3)	1.2309(3)	0.6158(2)	0.0488(7)
O9	0.0623(3)	0.8519(3)	0.86536(16)	0.0359(5)
O10	0.0233(3)	0.7753(3)	0.59688(19)	0.0417(6)
O11	0.3342(3)	1.0382(2)	0.46043(15)	0.0332(5)
O12	0.3653(3)	1.1016(3)	0.73226(18)	0.0427(6)
C1	0.6341(3)	0.4486(2)	0.80130(16)	0.0184(4)
C2	0.7544(3)	0.3197(3)	0.84626(19)	0.0254(5)
H2	0.852345	0.320640	0.858607	0.030
C3	0.7305(4)	0.1914(3)	0.8727(2)	0.0324(6)
H3	0.812831	0.103405	0.902068	0.039
C4	0.5861(4)	0.1907(3)	0.8562(2)	0.0311(6)
H4	0.570186	0.101944	0.875769	0.037
C5	0.4649(4)	0.3164(3)	0.81202(19)	0.0252(5)
H5	0.366255	0.315256	0.801112	0.030
C6	0.4917(3)	0.4445(3)	0.78404(16)	0.0191(4)
C7	0.5411(3)	0.6676(3)	0.59212(17)	0.0188(4)
C8	0.4821(4)	0.6745(3)	0.50987(19)	0.0256(5)
H8	0.372116	0.699197	0.503919	0.031
C9	0.5844(4)	0.6454(3)	0.4371(2)	0.0334(6)
H9	0.545323	0.646183	0.382199	0.040
C10	0.7436(4)	0.6150(3)	0.4448(2)	0.0338(7)
H10	0.812402	0.597567	0.394052	0.041
C11	0.8048(4)	0.6096(3)	0.52466(19)	0.0270(5)
H11	0.913966	0.589977	0.529073	0.032
C12	0.7020(3)	0.6337(3)	0.59823(17)	0.0201(4)
C13	0.7831(4)	0.7699(3)	0.9796(2)	0.0263(5)
C14	0.6906(3)	0.5343(3)	0.98610(18)	0.0225(5)
C15	0.9441(3)	0.5879(3)	0.8609(2)	0.0252(5)
C16	0.7324(3)	0.8800(3)	0.78004(18)	0.0216(5)
C17	0.4813(3)	0.8282(3)	0.90641(18)	0.0234(5)
C18	-0.0022(4)	1.1268(3)	0.6325(2)	0.0311(6)
C19	0.1114(3)	0.8818(3)	0.7927(2)	0.0242(5)
C20	0.0828(3)	0.8365(3)	0.6189(2)	0.0273(5)
C21	0.2823(3)	1.0048(3)	0.5315(2)	0.0247(5)
C22	0.3055(3)	1.0439(3)	0.7070(2)	0.0266(5)

3. COMPUTATIONAL DETAILS

3.1 General Details

DFT calculations related to **PP** dehydro oligomers (**DBODP**, **DBODP'**, TPPa and TPPb), ring strain energy evaluation and energetics for the P-P dehydro coupling catalytic cycle were performed with the ORCA program.[4] All geometry optimizations were run in redundant internal coordinates in the gas phase (unless otherwise stated), with tight convergence criteria. Harmonic frequency calculations verified the nature of ground states or TS having all real (positive) frequencies or only one imaginary frequency, respectively. For the study of **PP** dehydro oligomers, optimizations were performed employing the B3LYP[5,6] functional together with the RIJCOSX algorithm[4] and the Ahlrichs segmented def2-TZVP basis set.[7] The 2010 Grimme's semiempirical atom-pair-wise London dispersion correction (DFT-D4)[8] was employed. From these optimized geometries, all reported energies were corrected for the zero-point vibrational term and obtained by means of single-point (SP) calculations using the more extensive and polarized def2-QZVPP[9] basis set and the recently developed near-linear scaling domain-based local pair natural orbital (DLPNO) method[10] to achieve coupled cluster theory with single, double, and perturbative triple excitations (CCSD(T))[11] Solvent ($CHCl_3$) effects were taken into account with the CPCM solvation model.[12-13] In case of RSE studies and the mechanism of the P-P dehydrocoupling catalytic cycle, optimizations were carried out using Grimme's PBEh-3c[14] composite functional, final energies being evaluated with the double-hybrid-meta-GGA functional PWPB9[15,16] with Grimme's D3 semiempirical atom-pair-wise correction,[17,18] which makes use of the Becke-Johnson rationale damping,[19-21] and the def2-QZVPP basis set.
Overall energetics for dehydrocoupling reactions and conformational analysis for **DPB** and **PAN** were conducted with the Minnesota M062X functional[22] and the def2-TZVP basis set. Proton affinities (PA) were estimated at the B3LYP-D3/6-311++G(2d,p) level, using the expression PA = $-\Delta E_{ZPE} + \frac{5}{2}(RT)$. Computed NMR values were obtained with the gauge-independent atomic orbital (GIAO) method,[23,24] using the PBE1PBE[25] functional, the 6-311G++(2d,2p) basis set and the IEFPCM[26] solvation ($CHCl_3$) model implemented in Gaussian.[27] AIM analysis was performed with Multiwfn[28] software using the B3LYP-D3/6-311++G(2d,p) electron density. The electron density utilized for the AIM analysis was also employed in the computation of non-covalent interactions using the NCIplot[29-31] program.

3.2 Ring Strain Energy Evaluation

Ring strain energy (RSE) in the three dehydrodimers species studied herein was evaluated for only one of the two fused five-membered rings in each molecule. Although RSE reference values are most often obtained at a relatively high computational level (typically the CCSD(T)/def3-QZVPP//B3LYP-D3/def2-TZVP level is used[32-28]) through averaged (zero point-corrected) energy evaluation of appropriate homodesmotic reactions.[39] These reactions correspond to all possible endocyclic A-B bond cleavage using HA-BH reagents. Given the focus on an overall ring strain tendency in the comparison of dehydrodimers (**DBTP**, **DBODP** and **DBADP**, for E = PH, O and NH, respectively), the lower yet reliable PWPB95-D3/def2-QZVPP//PBEh-3c level was employed, with the homodesmotic P-E bond cleavage alone being evaluated. It is important to note that P-Ar and E-Ar bond cleavage homodesmotic reactions would result in products featuring long-range interactions. These interactions would introduce uncompensated factors on both sides of the homodesmotic reaction, which would distort the obtained strain energy value. It is reasonable to assume the strain less character of the second five-membered ring remaining in the resulting homodesmotic P-E bond cleavage product (**HD$_{P\text{-}Ecleav}$**).

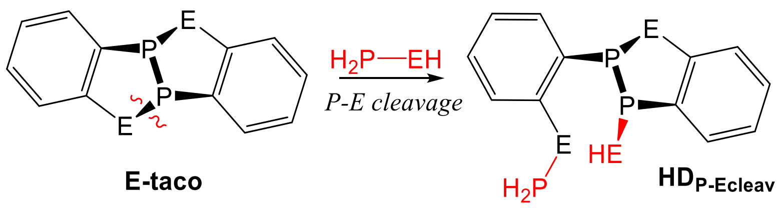

Figure S24: Homodesmotic P-E bond cleavage reactions used for RSE evaluation in dehydrodimers.

COMPUTED STRUCTURES

Computed Cartesian coordinates (Å) as well as electronic and zero-point energy correction (hartrees) are quoted at the PWPB9-D3/def2-QxVPP/PBEh-3c level unless otherwise stated. Enthalpy and Gibbs free energy corrections are also quoted when needed.

DBODP E = -1293.34445567 au [CPCM$_{CHCl3}$/DLPNO-CCSD(T)/def2-QZVPP]
ZPE = 0.16878792 au [CPCM$_{CHCl3}$/B3LYP-D4/def2-TZVP]
G_{corr} = 0.16878792 au [CPCM$_{CHCl3}$/B3LYP-D4/def2-TZVP]

C	1.666074	-0.902646	0.431962
C	3.680372	0.744385	-0.538802
C	1.716543	0.435841	0.832703
C	2.604147	-1.424118	-0.452161
C	3.604010	-0.591135	-0.934786
C	2.742602	1.251351	0.348453
H	2.543748	-2.463263	-0.746910
H	4.337780	-0.990837	-1.623432
H	2.799548	2.284685	0.667971
H	4.470341	1.379805	-0.916652
P	0.445932	1.038028	1.989892
O	-0.702032	1.720599	0.943685
P	-0.446656	-1.040759	1.988584
O	0.704050	-1.722840	0.944892
C	-1.715787	-0.437560	0.830477
C	-3.603220	0.592014	-0.935303
C	-2.744497	-1.250984	0.348316
C	-1.663777	0.900922	0.429670
C	-2.601285	1.423426	-0.454288
C	-3.682873	-0.742210	-0.537402
H	-2.803414	-2.283964	0.668651
H	-2.539462	2.462443	-0.749190
H	-4.475315	-1.375974	-0.912843
H	-4.336828	0.992794	-1.623489

3.3 Analysis of Crystal Packing Forces in PP

a.

b.

c.

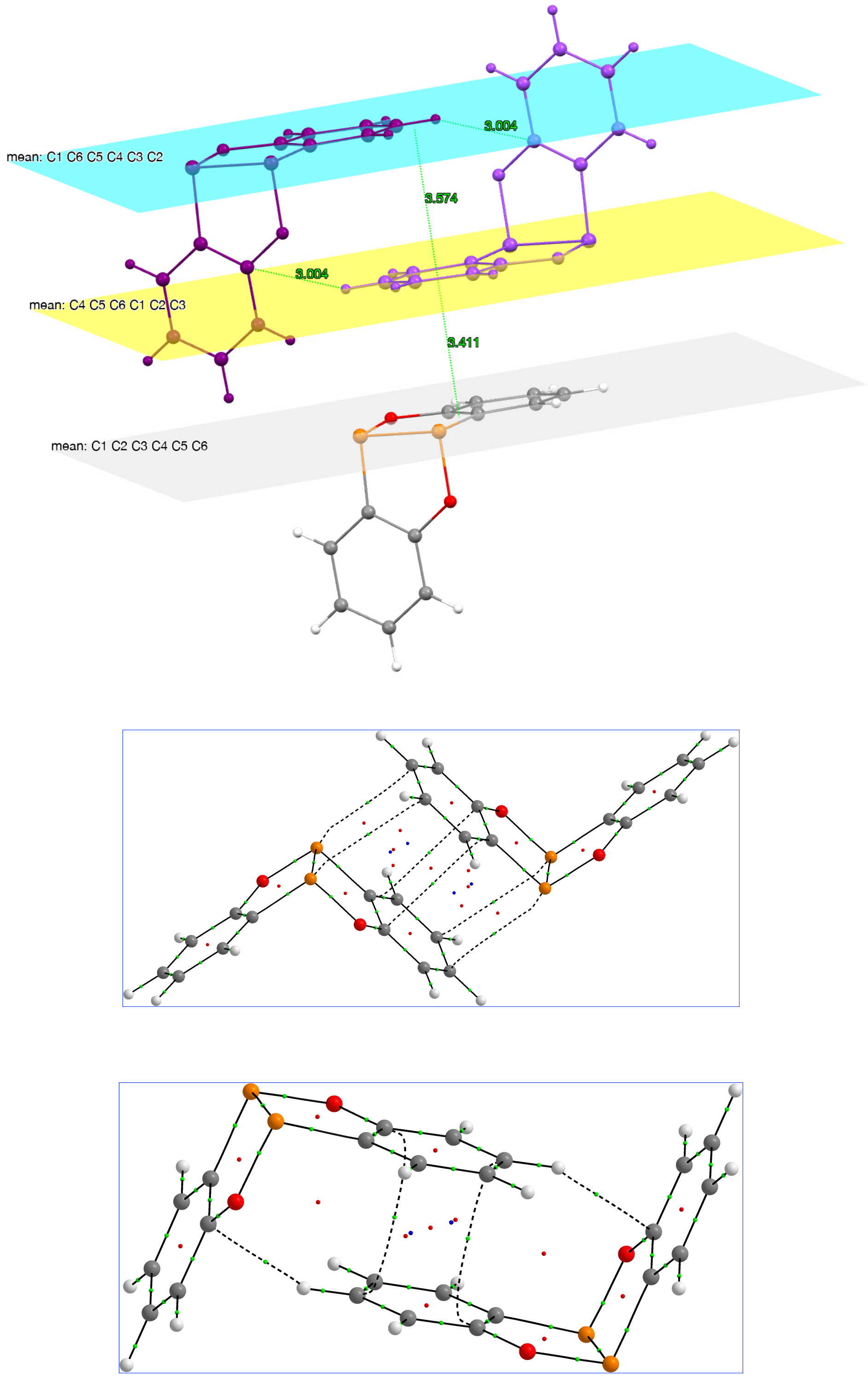

Figure S25: Computational analysis of non-covalent interactions of within two types of hypothetical dimers (**b**, **c**) from the X-ray structure (**a**) of **DBODP**.

DBODP π- and T-stacked dimer

E = -2586.70498805 au [$CPCM_{CHCl3}$/DLPNO-CCSD(T)/def2-QZVPP]

ZPE = 0.34242584 au [$CPCM_{CHCl3}$/B3LYP-D4/def2-TZVP]

G_{corr} = 0.29024431 au [$CPCM_{CHCl3}$/B3LYP-D4/def2-TZVP]

P	0.018516	0.001299	0.050864
C	0.069621	0.122142	1.865704
C	1.315522	0.083127	2.497026
C	1.421380	0.090788	3.882020
C	0.262134	0.153105	4.637523
C	-0.992003	0.209867	4.031624
C	-1.086474	0.180798	2.649850
O	2.450095	-0.006876	1.742290
H	2.396893	0.058820	4.347289
H	0.334789	0.154339	5.713734
H	-1.885204	0.264705	4.639281
H	-2.057168	0.200459	2.169707
P	2.280541	-0.043108	0.059368
C	2.260324	1.723601	-0.389586
C	1.022685	2.341905	-0.589802
C	0.937185	3.683634	-0.944568
C	2.108688	4.414563	-1.084570
C	3.354299	3.818592	-0.886697
C	3.426789	2.474085	-0.553421
O	-0.122815	1.616280	-0.448876
H	-0.033179	4.138060	-1.093413
H	2.049474	5.462061	-1.351822
H	4.259606	4.399879	-1.000406
H	4.390706	2.001751	-0.409409
C	0.615847	3.498633	3.486900
P	2.031162	3.585913	7.313480
C	1.973961	3.465759	5.498833
C	0.726137	3.507627	4.871500
C	1.772506	3.432289	2.727842
C	3.028416	3.373012	3.329706
C	3.127406	3.403391	4.711110
O	-0.405762	3.601675	5.629652
H	-0.360949	3.532626	3.024453
H	1.695986	3.427847	1.651980
H	3.919472	3.314860	2.719241
H	4.099564	3.381485	5.188180
P	-0.230798	3.636218	7.311943
C	-0.213497	1.868821	7.758534
C	1.023151	1.247126	7.954147
C	1.106310	-0.095208	8.307145
C	-0.066594	-0.823296	8.450113
C	-1.311296	-0.223954	8.256707
C	-1.381347	1.121080	7.925031
O	2.170053	1.970147	7.811275
H	2.075959	-0.552246	8.452640
H	-0.009222	-1.871200	8.716169
H	-2.217721	-0.803043	8.372686
H	-2.344490	1.596007	7.784353

DBODP π-stacked (outer) dimer

E = -2586.70423745 au [$CPCM_{CHCl3}$/DLPNO-CCSD(T)/def2-QZVPP]

ZPE = 0.34197869 au [$CPCM_{CHCl3}$/B3LYP-D4/def2-TZVP]

G_{corr} = 0.28987130 au [$CPCM_{CHCl3}$/B3LYP-D4/def2-TZVP]

P	0.692725	-0.078958	0.153794
C	0.237338	0.063340	1.905977
C	1.263453	0.220539	2.843467
C	0.990217	0.271766	4.204606
C	-0.325529	0.166192	4.626437
C	-1.365146	0.013935	3.709951
C	-1.081442	-0.045321	2.354983
O	2.558305	0.273091	2.421780
H	1.801664	0.390874	4.907437
H	-0.546393	0.201849	5.683393
H	-2.386904	-0.065984	4.056692
H	-1.880648	-0.179841	1.636505
P	2.858073	0.002831	0.772938
C	2.939781	1.687234	0.091628
C	1.766053	2.249125	-0.420316
C	1.759285	3.533117	-0.954862
C	2.940852	4.260421	-0.965131
C	4.123871	3.717613	-0.462366
C	4.121751	2.430363	0.054139
O	0.613440	1.525253	-0.411845
H	0.839544	3.946349	-1.346747
H	2.940392	5.262986	-1.374016
H	5.038782	4.294644	-0.481871
H	5.037060	1.997404	0.438462
C	-0.564659	3.469628	3.512896
P	1.844447	3.727185	6.793089
C	1.288907	3.678101	5.064839
C	-0.075807	3.518279	4.811969

C	0.328690	3.582232	2.459255
C	1.693068	3.757148	2.687909
C	2.168433	3.809608	3.987155
O	-0.948759	3.432698	5.857670
H	-1.623492	3.333791	3.342332
H	-0.045447	3.532476	1.445892
H	2.376765	3.852543	1.858173
H	3.226406	3.947042	4.173600
P	-0.313674	3.538582	7.426754
C	0.010777	1.794559	7.836369
C	1.289318	1.284472	7.590657
C	1.594187	-0.046409	7.854805
C	0.603314	-0.871181	8.367730
C	-0.675898	-0.380170	8.630555
C	-0.965136	0.951837	8.373900
O	2.258456	2.105082	7.098658
H	2.589566	-0.418954	7.653099
H	0.832766	-1.909857	8.569604
H	-1.436400	-1.033269	9.037394
H	-1.954010	1.342783	8.579362

3.4 Inversion at P in DBODP

The structure of **DBODP** is 54.6 kcal/mol more stable than an isomer having an inverted geometry at one of the phosphorus centers, and that there is a corresponding barrier of 67.8 kcal/mol to inversion at phosphorus that interconverts the two isomers

(**DBODP** P-inversion)‡

E = -1293.23569757 au [CPCM$_{CHCl3}$/DLPNO-CCSD(T)/def2-QZVPP]
ZPE = 0.16800373 au [CPCM$_{CHCl3}$/B3LYP-D4/def2-TZVP]
G$_{corr}$ = 0.13001628 au [CPCM$_{CHCl3}$/B3LYP-D4/def2-TZVP]
υ = 132 cm^{-1} [CPCM$_{CHCl3}$/B3LYP-D4/def2-TZVP]

P	-0.265099	0.808376	0.862485	P	1.317724	-0.358490	-0.053633
C	-0.985399	-0.704727	1.555836	C	1.372553	0.978071	-1.251360
C	0.122913	-1.303072	2.223266	C	0.087681	1.570697	-1.475905
C	-0.087070	-2.480359	2.947352	C	-0.126672	2.417493	-2.556222
C	-1.373250	-2.998842	3.049661	C	0.930708	2.696795	-3.404922
C	-2.453455	-2.390564	2.409574	C	2.200765	2.136335	-3.204815
C	-2.259461	-1.233499	1.655192	C	2.414242	1.278372	-2.147453
O	1.309546	-0.691803	2.116169	O	-0.941870	1.274394	-0.633813
H	0.754194	-2.959200	3.433487	H	-1.105138	2.853231	-2.706557
H	-1.537170	-3.896463	3.633456	H	0.771241	3.366168	-4.240620
H	-3.445411	-2.813151	2.503556	H	3.007107	2.374591	-3.885433
H	-3.096122	-0.755140	1.161312	H	3.387441	0.830788	-1.990612

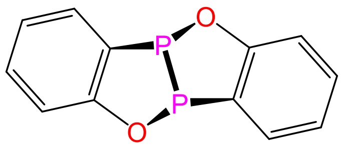

DBODP

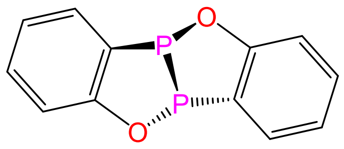

DBODP'

DBODP’

E = -1293.25754352 au [CPCM$_{CHCl3}$/DLPNO-CCSD(T)/def2-QZVPP]
ZPE = 0.16885702 au [CPCM$_{CHCl3}$/B3LYP-D4/def2-TZVP]
G$_{corr}$ = 0.1306718 au [CPCM$_{CHCl3}$/B3LYP-D4/def2-TZVP]

P	-0.002484	0.002358	-0.000136	P	1.562827	-1.506473	0.275341
C	0.006950	0.011806	1.830130	C	1.553005	-1.515788	-1.554956
C	1.383383	0.012140	2.200096	C	0.176579	-1.515948	-1.924936
C	1.769625	0.330454	3.491147	C	-0.209708	-1.834224	-3.216000
C	0.795437	0.658437	4.427837	C	0.764419	-2.162341	-4.152679
C	-0.554285	0.663293	4.083679	C	2.114152	-2.167377	-3.808512
C	-0.943652	0.361411	2.783908	C	2.503557	-1.865540	-2.508755
O	2.357078	-0.323760	1.256399	O	-0.797096	-1.179921	-0.981282
H	2.819158	0.303336	3.753467	H	-1.259257	-1.806951	-3.478241
H	1.095171	0.903354	5.438762	H	0.464673	-2.407242	-5.163603
H	-1.301101	0.913153	4.826034	H	2.860935	-2.417368	-4.550854
H	-1.989995	0.393405	2.508375	H	3.549902	-1.897689	-2.233246

3.5 Consideration of other possible dehydrocoupling products of PP

As it was reported that **DPB** could give rise to two different product types (dehydrodimer or dehydrooctomer) and that a derivative of **DPB** bearing methyl substituents at the 4 and 5 ring positions could provide a dehydropentamer (J. D. Masuda, A. J. Hoskin, T. W. Graham, C. Beddie, M. C. Fermin, N. Etkin, D. W. Stephan, "Catalytic P-H activation by Ti and Zr catalysts." *Chem. Eur. J.* **2006**, *12* (34), 8696-8707),the possibility of alternative, higher order dehydrooligomers of PP was explored briefly by theory. The two most obvious potential candidates are dehydrotetramers **TTPa** and **TTPb** (below). Both of these structures were found to be significantly higher in energy compared to **DBODP** (23.6 and 16.9 kcal/mol per DBODP unit, for **TTPa** and **TTPb**, respectively), which concurs with the preferential selectivity for the dehydrodimer **DBODP**.

a.

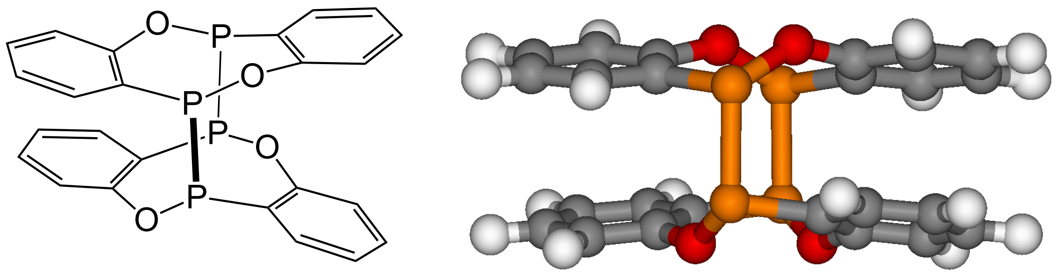

b.

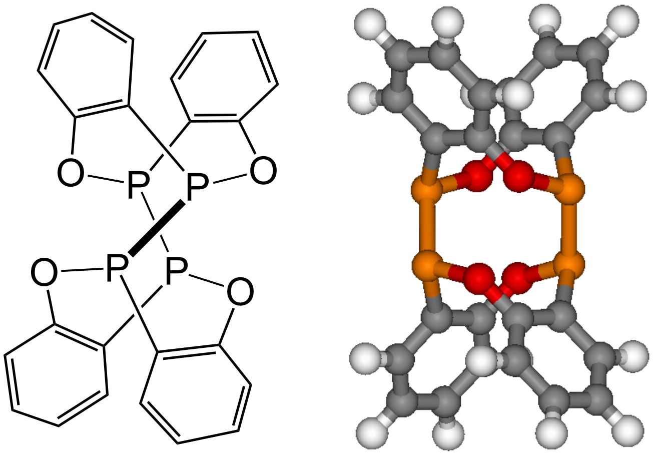

Figure S26. "Tetramers" **TTPa and TTPb** were refined but were determined to be much higher in energy compared to "dimer" **DBODP**

TTPa E = -2586.61871999 au [CPCM$_{CHCl3}$/DLPNO-CCSD(T)/def2-QZVPP]
ZPE = 0.34123544 au [CPCM$_{CHCl3}$/B3LYP-D4/def2-TZVP]
G_{corr} = 0.28996955 au [CPCM$_{CHCl3}$/B3LYP-D4/def2-TZVP]

P	0.037298	0.006602	0.081653	O	-0.019328	0.222146	1.717229

C	0.755479	0.019839	2.829965
C	0.543649	-1.144478	3.558824
C	1.215891	-1.351845	4.753164
C	2.090550	-0.385150	5.237395
C	2.283408	0.782576	4.517399
C	1.634036	0.999379	3.297117
P	2.022458	2.568803	2.461665
H	-0.150499	-1.878952	3.173284
H	1.051101	-2.266890	5.307044
H	2.610290	-0.535336	6.174578
H	2.946717	1.547982	4.904619
O	0.725280	2.803111	1.468169
C	0.422297	2.850053	0.132099
C	0.383439	4.098776	-0.476859
C	-0.027465	4.219192	-1.795225
C	-0.421878	3.091990	-2.507941
C	-0.399870	1.851971	-1.890597
C	0.036043	1.706187	-0.569083
H	-0.729468	0.975115	-2.436747
H	-0.754705	3.180025	-3.533839
H	-0.044408	5.195203	-2.262516
H	0.680968	4.965761	0.097369
P	3.975320	1.898888	1.554796
C	3.687236	2.599849	-0.099819
C	3.248101	1.893953	-1.221657
C	3.059790	2.543483	-2.435870
C	3.293599	3.905625	-2.541853
C	3.737052	4.624500	-1.437028
C	3.941930	3.969114	-0.233796
O	3.113282	0.529948	-1.203742
H	2.727473	1.965460	-3.287557
H	3.134180	4.402851	-3.489790
H	3.933094	5.685835	-1.515558
H	4.310711	4.522784	0.622509
P	1.985926	-0.626061	-0.861050
C	2.666772	-1.457816	0.607470
C	3.598270	-0.916330	1.495434
C	4.042062	-1.651073	2.588552
C	3.551866	-2.927445	2.816512
C	2.628032	-3.485946	1.939775
C	2.203310	-2.757241	0.840754
O	4.200503	0.291363	1.254957
H	1.501397	-3.199731	0.142683
H	2.249897	-4.486320	2.104998
H	3.897145	-3.486564	3.676422
H	4.770997	-1.205421	3.251849

TTPb

E = -2586.63969162 au [$CPCM_{CHCl3}$/DLPNO-CCSD(T)/def2-QZVPP]
ZPE = 0.34208187 au [$CPCM_{CHCl3}$/B3LYP-D4/def2-TZVP]
G_{corr} = 0.29013875 au [$CPCM_{CHCl3}$/B3LYP-D4/def2-TZVP]

P	0.217451	-0.211382	-0.264562
C	-0.190346	-0.215013	1.510226
C	0.713624	0.037928	2.548069
C	0.302527	-0.015752	3.875366
C	-1.015270	-0.322866	4.183063
C	-1.929377	-0.583379	3.167701
C	-1.511940	-0.530841	1.845824
O	2.010588	0.408556	2.316168
H	1.026110	0.190726	4.652847
H	-1.325832	-0.361608	5.219534
H	-2.957289	-0.825544	3.403118
H	-2.218075	-0.733621	1.050280
P	3.282018	-0.525039	1.758331
C	2.507983	-2.155721	1.521754
C	1.561752	-2.464865	0.538496
C	1.007086	-3.737711	0.463195
C	1.390703	-4.717308	1.368239
C	2.325989	-4.430518	2.356892
C	2.872961	-3.157653	2.428286
O	1.186892	-1.566321	-0.423529
H	0.281700	-3.944381	-0.312526
H	0.953959	-5.705736	1.301352
H	2.625939	-5.190742	3.065974
H	3.600513	-2.926809	3.196399
P	1.061725	1.818061	-0.523732
C	1.879235	1.680553	-2.144481
C	2.856089	0.735913	-2.477863
C	3.443073	0.737475	-3.738391
C	3.062139	1.682135	-4.680950
C	2.096987	2.633805	-4.369143
C	1.517430	2.628232	-3.108782
O	3.232412	-0.260935	-1.618705
H	4.191751	-0.009974	-3.965169
H	3.524670	1.675263	-5.659839
H	1.799290	3.373881	-5.100133
H	0.766330	3.367051	-2.858494
P	4.166365	-0.147212	-0.234584
C	4.531354	1.634398	-0.142172
C	3.597120	2.637694	0.138652
C	3.977637	3.975009	0.159169
C	5.294481	4.327270	-0.100622
C	6.238369	3.346947	-0.387741

C	5.851456	2.014981	-0.409402	H	5.581022	5.371178	-0.081828
O	2.297774	2.358505	0.466029	H	7.265629	3.617152	-0.593656
H	3.231270	4.724938	0.385450	H	6.580776	1.246533	-0.633489

3.6 Conformational Analysis and Dihydrogen Bonding in DPB and PAN

A detailed conformational analysis was carried out to evaluate the possible role of non-covalent interactions, including dihydrogen bonding, in substrates relevant to the present dehydrocoupling chemistry. Previous combined experimental and computational work on *ortho*-phosphinophenol (**PP**) demonstrated the existence of four conformational isomers, the lowest-energy form of which features short PH···HO contacts ($d_{H\cdots H}$ = 2.172 Å) consistent with dihydrogen bonding.[40] Given that such interactions may indicate an increased propensity toward hydrogen release or activation in main-group compounds, analogous analyses were extended to *ortho*-diphosphinobenzene (**DPB**) and *ortho*-phosphinoaniline (**PAN**).

Four conformational isomers of **DPB** and three of **PAN** were located at the M06-2X/def2-TZVP level of theory. None of the identified conformers showed evidence for conventional P···HE (E = N or P) hydrogen bonding. In **PAN**, the lowest-energy conformer exhibits PH···HN contact distances of 2.216 and 2.352 Å; however, atoms-in-molecules (AIM) analysis revealed no bond critical points between the interacting hydrogen atoms, indicating the absence of well-defined dihydrogen bonds. Reduced density gradient (RDG) and non-covalent interaction (NCI) analyses are nevertheless consistent with weak attractive interactions of either dihydrogen or hydrogen-bonding character.

For **DPB**, the lowest-energy conformer shows a PH···HP separation of 2.624 Å, suggesting minimal attractive interaction. In contrast, a higher-energy conformer (DPB2) displays two PH···HP contacts of 2.319 Å, accompanied by bond critical points (ρ = 0.0091 au), consistent with weak dihydrogen bonding interactions. These values fall within the range previously reported for PH···HP interactions, which are dominated by dispersive forces and typically exhibit interaction energies between 0.30 and 1.1 kcal·mol^{-1} with H···H distances of 2.046–2.367 Å.[41]

Overall, although weak PH···HP or PH···HN interactions can be identified in selected conformers of **DPB** and **PAN**, they are significantly less pronounced than those observed for **PP**. Consequently, clear correlations between the presence of dihydrogen bonding and the experimentally observed propensity for hydrogen transfer or dehydrocoupling across **PP**, **DPB**, and **PAN** are not straightforward. These results nonetheless highlight the role of conformational flexibility and subtle non-covalent effects in modulating hydrogen activation processes in organophosphorus compounds.

DPB E = -916.09452 au [M06-2X/def2-TZVP]
ZPE = 0.116944 au [M06-2X/def2-TZVP]
H_{corr} = -915.96797 au [M06-2X/def2-TZVP]
G_{corr} = -916.01221 au [M06-2X/def2-TZVP]

C	-0.0138990	-0.7019570	0.0090070
C	-2.4348510	0.6934290	0.0027280
C	-0.0139000	0.7019570	0.0090050
C	-1.2314830	-1.3811000	0.0129460
C	-2.4348510	-0.6934310	0.0027310
C	-1.2314840	1.3811000	0.0129420
H	-1.2381190	-2.4642180	0.0238050
H	-3.3686260	-1.2404550	0.0008960
H	-1.2381210	2.4642170	0.0237960
H	-3.3686270	1.2404520	0.0008900
P	1.5708300	-1.6201040	-0.1091410
H	2.0682520	-1.3122280	1.1791580
H	1.0574690	-2.8736570	0.2851670
P	1.5708280	1.6201060	-0.1091390
H	1.0574670	2.8736550	0.2851820
H	2.0682510	1.3122180	1.1791570

Table S20: **Summary of Energies for DPB conformational isomers**

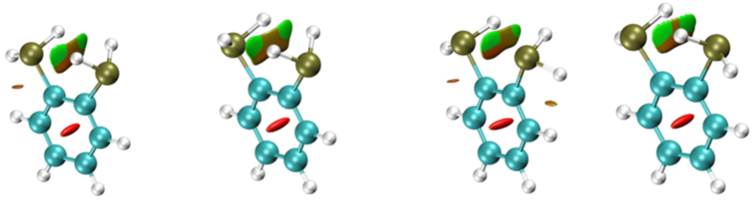

	DPB1	DPB2	DPB3	DPB4
**H ENTHALPIES au**	-915.968513	-915.966392	-915.967971	-915.968160
**relative ΔH kcal/mol**	**0**	1.33	0.34	0.22
**G FREE ENERGIES au**	-916.010998	-916.009978	-916.012211	-916.011281
**relative ΔG kcal/mol**	0.76	1.40	**0**	0.58

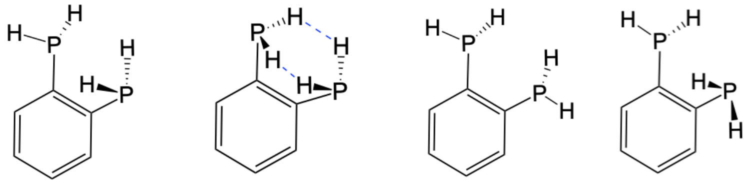

DPB1 **DPB2** **DPB3** **DPB4**

DPB1 E = -916.0953 au [M06-2X/def2-TZVP]

C	0.0087060	0.6992940	0.0120880	H	1.2337350	-2.4645650	-0.0038240
C	2.4389250	-0.6939130	0.0022320	H	3.3740530	-1.2382250	0.0003320
C	0.0138300	-0.7062880	0.0000440	P	-1.5608510	1.6406100	-0.1090400
C	1.2261960	1.3781770	0.0088610	H	-2.1199480	1.2604750	1.1311960
C	2.4317110	0.6913000	0.0026450	H	-1.0456800	2.8614900	0.3768320
C	1.2336250	-1.3809480	0.0011460	P	-1.4902620	-1.7641510	-0.0147150
H	1.2351340	2.4612720	0.0172050	H	-2.1427990	-1.1937820	1.1035970
H	3.3632700	1.2425960	0.0031670	H	-2.2490310	-1.0018860	-0.9342560

$d_{PH\cdots HP}$ = 2.454 Å dPH•••P = 2.853Å

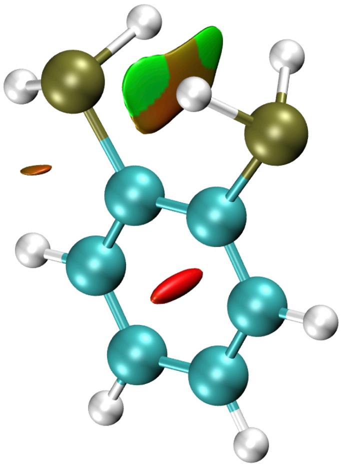

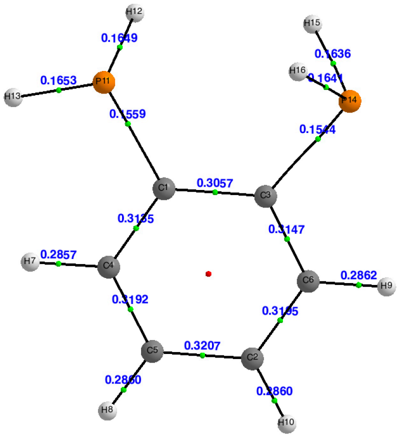

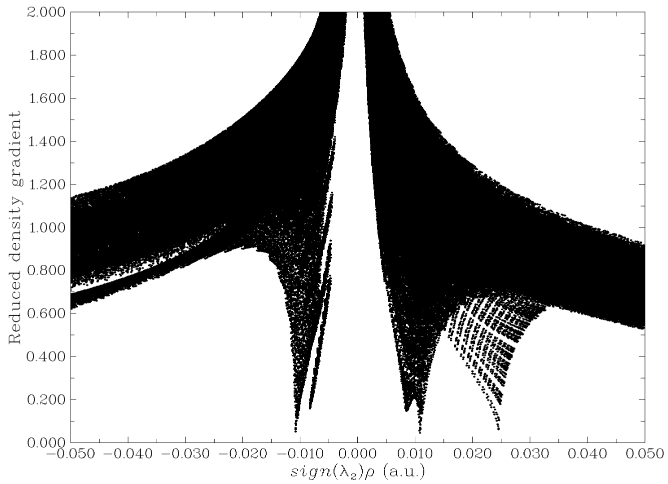

DPB2 E = -916.09324 au [M06-2X/def2-TZVP]

C	0.0026930	-0.7047360	-0.0005260	H	1.2265010	2.4597450	0.0031000
C	2.4327320	0.6911550	0.0003410	H	3.3649500	1.2409680	0.0006100
C	0.0026930	0.7047360	0.0005260	P	-1.4755330	-1.8019380	-0.0029780
C	1.2237560	-1.3762810	-0.0012240	H	-2.1909760	-1.1828750	1.0467780
C	2.4327320	-0.6911550	-0.0003410	H	-2.2225610	-1.1356030	-0.9999730
C	1.2237560	1.3762810	0.0012240	P	-1.4755330	1.8019380	0.0029780
H	1.2265010	-2.4597450	-0.0031000	H	-2.1909760	1.1828750	-1.0467780
H	3.3649500	-1.2409680	-0.0006090	H	-2.2225610	1.1356030	0.9999730

$d_{PH\bullet\bullet\bullet HP}$ = 2.319Å

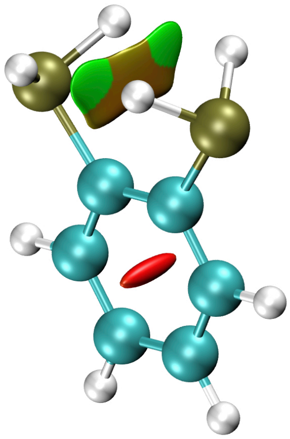

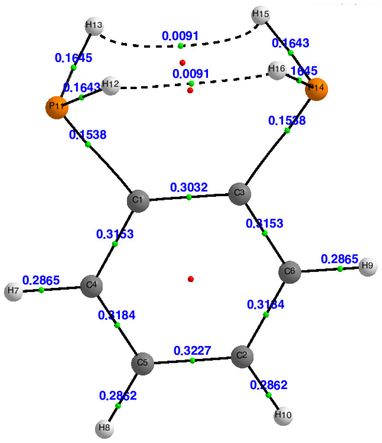

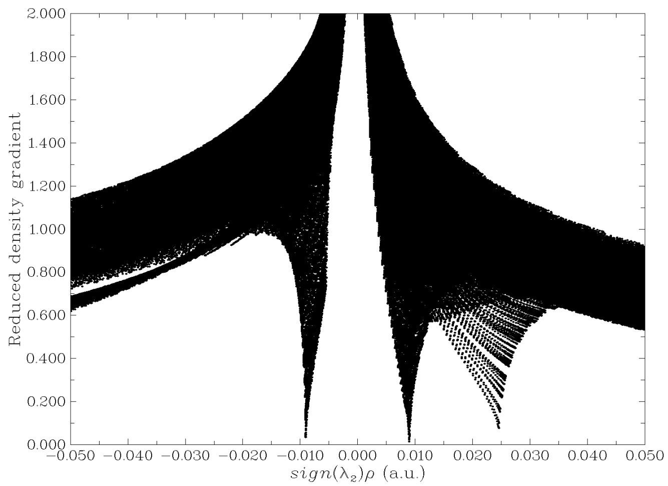

DPB3 E = -916.09452 au [M06-2X/def2-TZVP]

C	-0.0138990	-0.7019570	0.0090070
C	-2.4348510	0.6934290	0.0027280
C	-0.0139000	0.7019570	0.0090050
C	-1.2314830	-1.3811000	0.0129460
C	-2.4348510	-0.6934310	0.0027310
C	-1.2314840	1.3811000	0.0129420
H	-1.2381190	-2.4642180	0.0238050
H	-3.3686260	-1.2404550	0.0008960
H	-1.2381210	2.4642170	0.0237960
H	-3.3686270	1.2404520	0.0008900
P	1.5708300	-1.6201040	-0.1091410
H	2.0682520	-1.3122280	1.1791580
H	1.0574690	-2.8736570	0.2851670
P	1.5708280	1.6201060	-0.1091390
H	1.0574670	2.8736550	0.2851820
H	2.0682510	1.3122180	1.1791570

$d_{PH\bullet\bullet\bullet HP}$ = 2.624Å

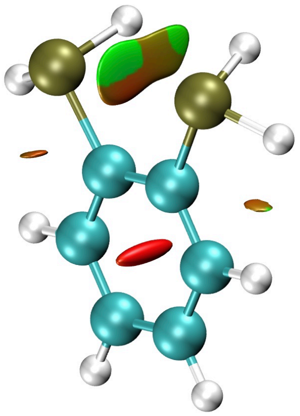

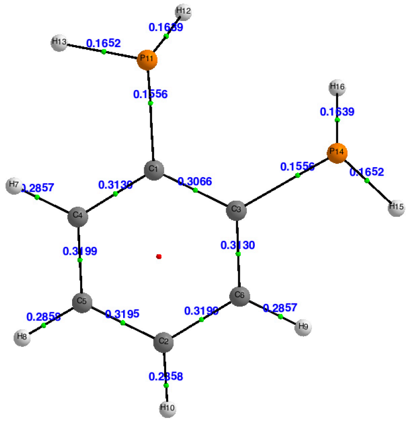

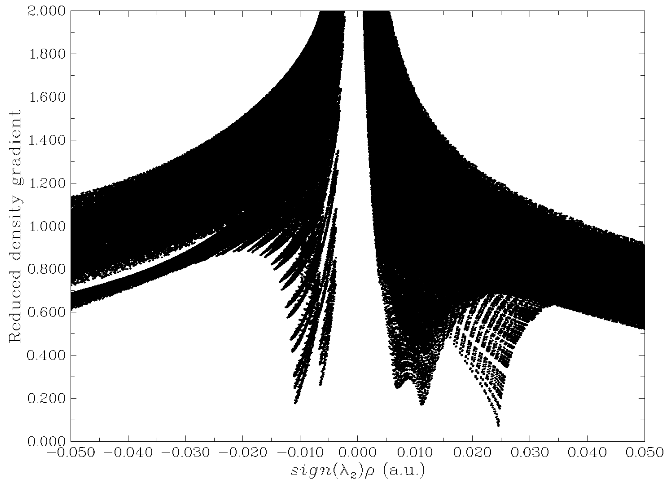

DPB4 E = -916.0947 au [M06-2X/def2-TZVP]

C	0.0102050	-0.7008100	-0.0086910
C	2.4316420	0.6939560	-0.0010350
C	0.0102060	0.7008110	0.0086910
C	1.2287160	-1.3801060	-0.0013780
C	2.4316420	-0.6939580	0.0010350
C	1.2287180	1.3801050	0.0013780
H	1.2348030	-2.4639210	-0.0032120
H	3.3652640	-1.2411160	0.0005010
H	1.2348050	2.4639210	0.0032120
H	3.3652660	1.2411130	-0.0005020
P	-1.5508320	-1.6560250	0.1149460
H	-2.2361400	-1.0399580	-0.9584050
H	-1.1248400	-2.7586240	-0.6552760
P	-1.5508310	1.6560260	-0.1149460
H	-2.2361420	1.0399530	0.9583990
H	-1.1248420	2.7586220	0.6552830

$d_{PH\bullet\bullet\bullet P}$ = 2.907Å

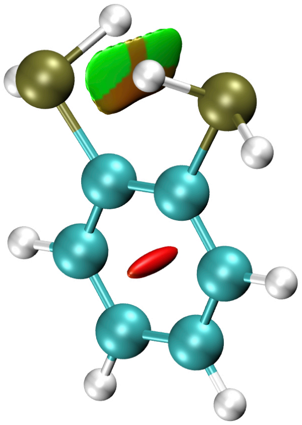

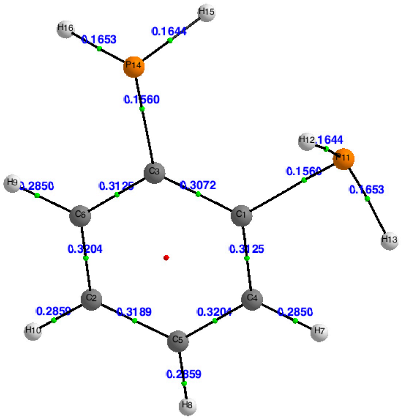

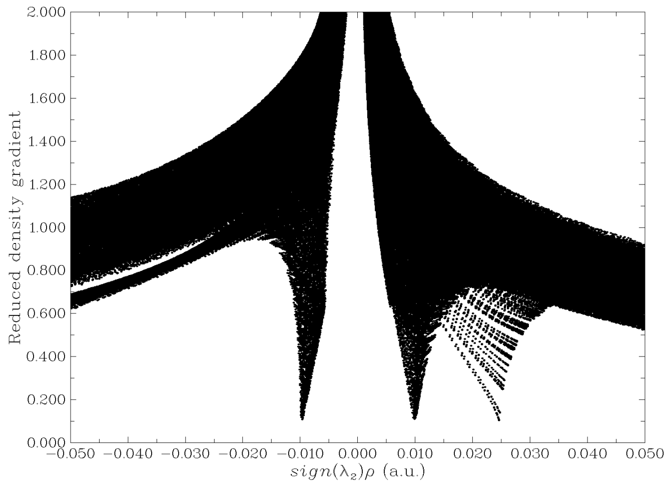

Table S21: **Summary of Energies for PAN conformational isomers**

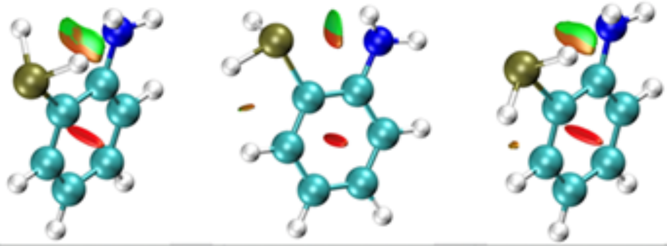

	PAN1	PAN2	PAN3
H ENTHALPIES au	-629.388519	-629.386918	-629.385679
relative ΔH kcal/mol	0	1.00	1.78
G FREE ENERGIES au	-629.428703	-629.427974	-629.426653
relative ΔG kcal/mol	0	0.46	1.29

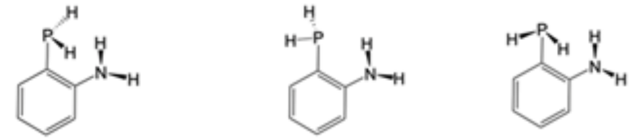

PAN1 PAN2 PAN3

PAN1 E = -629.52348 au [M06-2X/def2-TZVP]

C	-0.3930140	-0.3517660	0.0034890
C	2.3815270	-0.0527110	-0.0019980
C	0.1743030	0.9359910	-0.0032960
C	0.4533130	-1.4602290	0.0097810
C	1.8321410	-1.3284270	0.0078750
C	1.5670260	1.0639620	-0.0073580
H	0.0087710	-2.4486880	0.0139390
H	2.4668050	-2.2035940	0.0130290
H	2.0043240	2.0556180	-0.0169500
H	3.4565900	0.0758590	-0.0046000
P	-2.1897380	-0.6774440	-0.0126010
H	-2.5734560	0.1694620	1.0591870
H	-2.5626780	0.2755410	-0.9911470
N	-0.6167440	2.0692470	-0.0601190
H	-1.5594630	1.9802210	0.2817160
H	-0.1693980	2.9315980	0.2037180

$d_{NH\bullet\bullet\bullet H2P}$ = 2.216 and 2.352Å

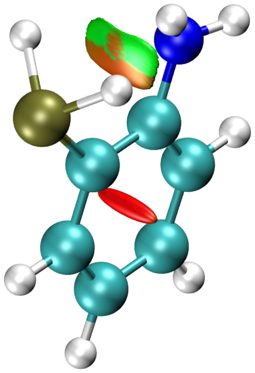

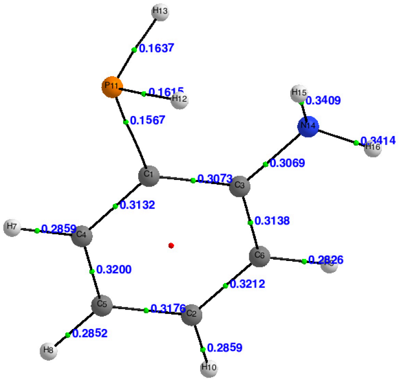

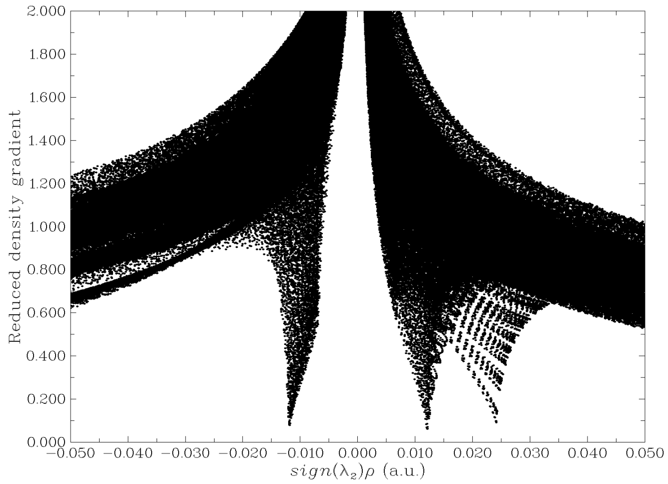

PAN2 E = -629.52173 au [M06-2X/def2-TZVP]

C	0.3946660	-0.3462730	-0.0009930
C	-2.3833240	-0.0728310	0.0119250
C	-0.1861150	0.9331880	0.0000960
C	-0.4378270	-1.4630490	-0.0113200
C	-1.8182330	-1.3411340	-0.0068300
C	-1.5776780	1.0509650	0.0145060
H	0.0084660	-2.4500810	-0.0169350
H	-2.4434000	-2.2233990	-0.0127730
H	-2.0217840	2.0396170	0.0253910
H	-3.4594930	0.0437040	0.0203450
P	2.2180010	-0.4808360	-0.0742770
H	2.4936300	-0.3215640	1.3035170
H	2.2867570	-1.8875960	-0.0445030
N	0.6091780	2.0746770	0.0545990
H	1.5149000	1.9956740	-0.3868500
H	0.1377180	2.9282560	-0.2005370

$d_{NH\bullet\bullet\bullet PH2}$ = 2.593Å

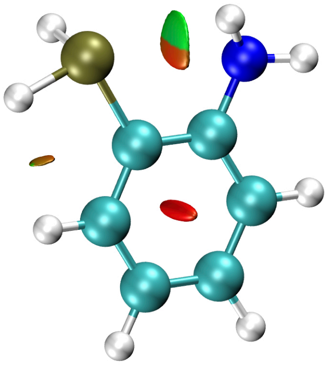

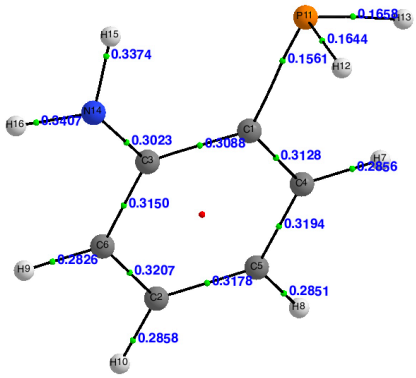

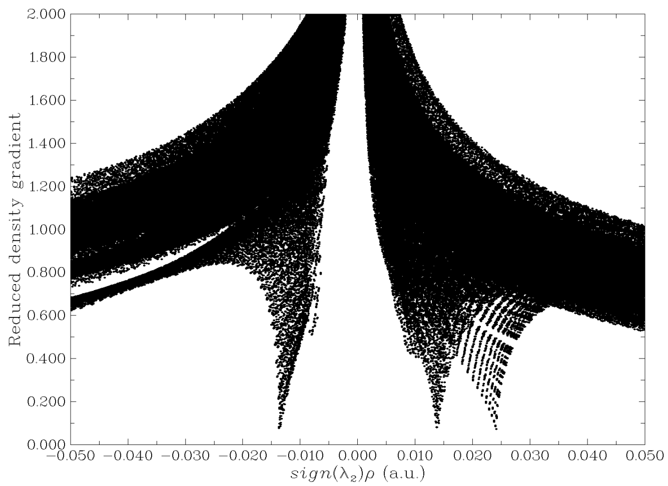

PAN3 E = -629.52039 au [M06-2X/def2-TZVP]

C	-0.3933400	-0.3475720	0.0257620	H	2.0125240	2.0470230	-0.0280580
C	2.3836210	-0.0644080	-0.0157500	H	3.4591530	0.0558550	-0.0330370
C	0.1827160	0.9320260	0.0034250	P	-2.2068930	-0.5240050	-0.1354310
C	0.4423820	-1.4613850	0.0390280	H	-2.2888370	-1.8091180	0.4412480
C	1.8224130	-1.3337500	0.0150730	H	-2.5819560	0.1268190	1.0684080
C	1.5726510	1.0564070	-0.0145900	N	-0.6245610	2.0661840	-0.0549680
H	0.0005710	-2.4506070	0.0625780	H	-1.4956730	2.0171500	0.4506010
H	2.4505900	-2.2139020	0.0234680	H	-0.1437090	2.9356740	0.1133460

$d_{NH\bullet\bullet\bullet HP}$ = 2.266Å

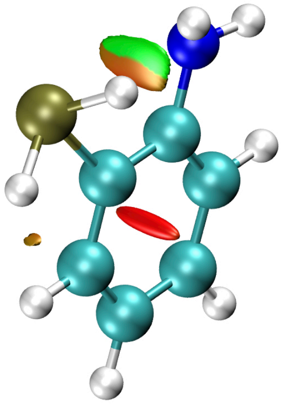

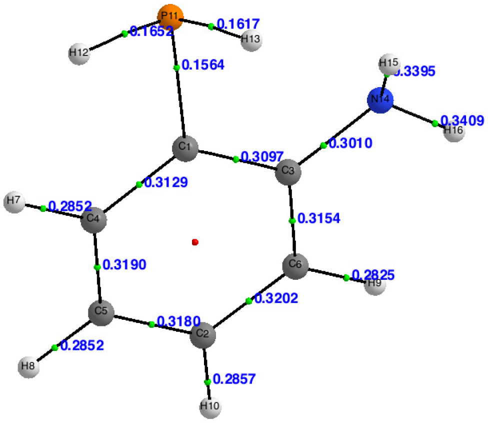

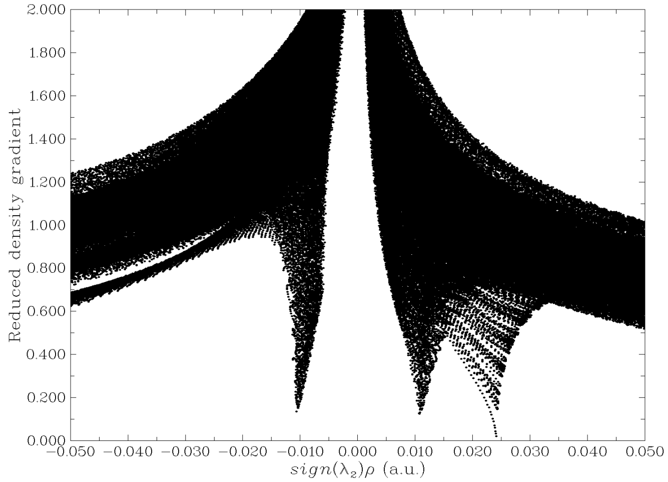

3.7 Structures and Energies for dehydrocoupling reactions and RSEs

DBTP E = -1828.6854 au [M06-2X/def2-TZVP]
ZPE = 0.185451 au [M06-2X/def2-TZVP]
H_{corr} = -1828.4851 au [M06-2X/def2-TZVP]
G_{corr} = -1828.541 au [M06-2X/def2-TZVP]

P	-0.6340710	0.9044320	-1.6923090
P	0.6340590	-0.9043110	-1.6923780
C	1.7698650	0.8482190	0.1881450
C	2.3866060	-1.4701310	0.4366540
H	2.3086020	-2.5031290	0.1175760
C	-1.6568800	0.4835800	-0.2232900
C	1.6568780	-0.4835640	-0.2233350
C	-2.5890160	-1.1681990	1.2681830
H	-2.6602050	-2.1970560	1.6002350
C	-2.3866030	1.4701000	0.4367760
H	-2.3085990	2.5031220	0.1177710
C	2.5890220	1.1681090	1.2682510
H	2.6602130	2.1969410	1.6003760
C	-1.7698650	-0.8482330	0.1880960
C	-3.3055880	-0.1789560	1.9264230
H	-3.9365010	-0.4369320	2.7668280
C	3.3056000	0.1788180	1.9264150
H	3.9365170	0.4367340	2.7668350
C	3.2024660	-1.1407300	1.5090560
H	3.7538220	-1.9176840	2.0226870
C	-3.2024560	1.1406230	1.5091580
H	-3.7538070	1.9175400	2.0228490
P	-0.8332850	-2.1819190	-0.6632420
P	0.8332810	2.1819660	-0.6630940
H	1.6617660	2.2968870	-1.8064340
H	-1.6617760	-2.2967650	-1.8065850

d_{PP} = 2.209/2.201 Å

DBTP E = -1828.504010073 au [PWPB95-D3/def2-QZVPP]
ZPE = 0.19033336 au [PBEh-3c]

P	-0.631685	0.907912	-1.680839
P	0.631685	-0.907795	-1.680906
C	1.777217	0.843915	0.193352
C	2.400336	-1.471829	0.427350
H	2.323661	-2.504326	0.108403
C	-1.662591	0.485166	-0.220247
C	1.662590	-0.485159	-0.220284
C	-2.601820	-1.161611	1.268009
H	-2.672500	-2.187892	1.606908
C	-2.400337	1.471787	0.427461
H	-2.323663	2.504308	0.108592
C	2.601821	1.161505	1.268097
H	2.672504	2.187759	1.607074
C	-1.777216	-0.843939	0.193289
C	-3.325023	-0.172251	1.916314
H	-3.961086	-0.428373	2.753056
C	3.325024	0.172095	1.916327
H	3.961087	0.428153	2.753088
C	3.222477	-1.144846	1.494259
H	3.779644	-1.922404	1.999920
C	-3.222478	1.144723	1.494346
H	-3.779646	1.922241	2.000065
P	-0.831846	-2.180410	-0.638639
P	0.831847	2.180447	-0.638475
H	1.662154	2.343971	-1.777272
H	-1.662155	-2.343847	-1.777446

H_2P-PH_2 E = -685.027227063 au [PWPB95-D3/def2-QZVPP]
ZPE = 0.03658084 au [PBEh-3c]

P	-0.021551	-0.014401	-0.020757
H	-0.023196	-0.002359	1.394518
H	1.392624	-0.004443	-0.076955
P	-0.171286	-2.241561	-0.161727
H	-1.585489	-2.251124	-0.105651
H	-0.169450	-2.253166	-1.577026

HD(**DBTP**)$_{PPcleav}$ E = -1293.34445567 au [PWPB95-D3/def2-QZVPP]

ZPE = 0.22833324 au [PBEh-3c]

P	-0.434537	-1.041735	1.483820
P	0.434505	1.041975	1.483681
C	1.651664	-0.880850	-0.086139
C	2.656804	1.300240	-0.240643
H	2.694098	2.339103	0.062561
C	-1.677218	-0.462180	0.288165
C	1.677221	0.462246	0.288147
C	-2.572559	1.381885	-0.998599
H	-2.538955	2.425048	-1.282261
C	-2.656788	-1.300251	-0.240528
H	-2.694090	-2.339070	0.062826
C	2.572596	-1.382007	-0.998326
H	2.539000	-2.425211	-1.281837
C	-1.651651	0.880861	-0.086316
C	-3.523111	0.526475	-1.527026
H	-4.241030	0.912471	-2.238856
C	3.523162	-0.526673	-1.526852
H	4.241099	-0.912772	-2.238607
C	3.573968	0.812831	-1.153918
H	4.327448	1.466380	-1.571059
C	-3.573928	-0.812975	-1.153898
H	-4.327396	-1.466584	-1.570964
O	-0.745958	1.707764	0.470928
O	0.745957	-1.707672	0.471202

PP E = -649.39633 au [M06-2X/def2-TZVP]
ZPE = 0.113793 au [M06-2X/def2-TZVP]
H_{corr} = -649.27413 au [M06-2X/def2-TZVP]
G_{corr} = -649.31446 au [M06-2X/def2-TZVP]

C	-0.3970090	-0.3383940	-0.0000030
C	2.3799110	-0.0481040	-0.0000180
C	0.1878060	0.9339870	0.0000130
C	0.4437390	-1.4529910	-0.0000250
C	1.8219520	-1.3207680	-0.0000320
C	1.5717640	1.0745030	0.0000050
H	-0.0028590	-2.4402600	-0.0000370
H	2.4535810	-2.1982930	-0.0000500
H	1.9885270	2.0727310	0.0000170
H	3.4556680	0.0725160	-0.0000240
O	-0.5401920	2.0754210	0.0000350
H	-1.4802240	1.8618920	0.0000520
P	-2.2001000	-0.6391160	0.0000040
H	-2.5703150	0.2626600	1.0291760
H	-2.5703320	0.2627200	-1.0291090

DBODP E = -1295.267 au [M06-2X/def2-TZVP]
ZPE = 0.172885 au [M06-2X/def2-TZVP]
H_{corr} = -1295.0811 au [M06-2X/def2-TZVP]
G_{corr} = -1295.1331 au [M06-2X/def2-TZVP]

P	-0.4330040	-1.0458370	1.5393800
P	0.4329770	1.0460830	1.5392200
C	1.6338770	-0.8822110	-0.0449360
C	2.6011540	1.3047060	-0.2690140
H	2.6319630	2.3499970	0.0140860
C	-1.6496780	-0.4649500	0.3099400
C	1.6496700	0.4650010	0.3098910
C	-2.5294110	1.3932610	-0.9756320
H	-2.4887140	2.4432530	-1.2305860
C	-2.6011480	-1.3047490	-0.2688510
H	-2.6319610	-2.3499950	0.0144130
C	2.5294280	-1.3934160	-0.9753660
H	2.4887340	-2.4434490	-1.2301530
C	-1.6338800	0.8822050	-0.0451020
C	-3.4516970	0.5366800	-1.5522660
H	-4.1516340	0.9257680	-2.2805760
C	3.4517280	-0.5369300	-1.5521160
H	4.1516810	-0.9261360	-2.2803480
C	3.4939690	0.8108570	-1.2046190
H	4.2235070	1.4669820	-1.6592200
C	-3.4939430	-0.8110500	-1.2045550
H	-4.2234700	-1.4672490	-1.6590670
O	-0.7488100	1.7200470	0.5603730
O	0.7487950	-1.7199550	0.5606550

d_{PP} = 2.264 Å

DBODP E = -1295.110085385 au [PWPB95-D3/def2-QZVPP]
ZPE = 0.17719057 au [PBEh-3c]

P	-0.434537	-1.041735	1.483820
P	0.434505	1.041975	1.483681
C	1.651664	-0.880850	-0.086139
C	2.656804	1.300240	-0.240643
H	2.694098	2.339103	0.062561
C	-1.677218	-0.462180	0.288165
C	1.677221	0.462246	0.288147
C	-2.572559	1.381885	-0.998599
H	-2.538955	2.425048	-1.282261
C	-2.656788	-1.300251	-0.240528
H	-2.694090	-2.339070	0.062826
C	2.572596	-1.382007	-0.998326
H	2.539000	-2.425211	-1.281837
C	-1.651651	0.880861	-0.086316
C	-3.523111	0.526475	-1.527026
H	-4.241030	0.912471	-2.238856
C	3.523162	-0.526673	-1.526852
H	4.241099	-0.912772	-2.238607
C	3.573968	0.812831	-1.153918
H	4.327448	1.466380	-1.571059
C	-3.573928	-0.812975	-1.153898
H	-4.327396	-1.466584	-1.570964
O	-0.745958	1.707764	0.470928
O	0.745957	-1.707672	0.471202

H_2P-OH E = -418.344231016 au [PWPB95-D3/def2-QZVPP]
ZPE = 0.03266545 au [PBEh-3c]

P	-0.239858	0.052387	-0.169405
H	-0.050690	-0.190345	1.215061
H	1.128477	-0.189775	-0.453192
O	-0.881518	-1.413230	-0.623246
H	-1.655184	-1.267101	-1.170432

HD(**DBODP**)$_{POcleav}$ E = -1713.458680095 au [PWPB95-D3/def2-QZVPP]
ZPE = 0.21124652 au [PBEh-3c]

P	1.089926	0.582811	-1.027949
P	0.064741	-0.272317	0.751426
C	2.738358	0.330500	0.970072
C	1.704163	-0.235277	3.063151
H	0.824071	-0.509063	3.631304
C	0.630416	2.304843	-0.588956
C	1.601045	-0.032326	1.690209
C	-1.156775	3.937694	-0.545095
H	-2.196124	4.196495	-0.707262
C	1.517602	3.278798	-0.148498
H	2.560131	3.035246	0.005003
C	3.967311	0.477669	1.600472
H	4.841390	0.752319	1.025148
C	-0.702080	2.653657	-0.794997
C	-0.260898	4.893039	-0.087340
H	-0.609669	5.897733	0.110423
C	4.046645	0.264805	2.965971
H	5.000524	0.381747	3.463552
C	2.921920	-0.087592	3.704012
H	2.998463	-0.245300	4.770983
C	1.072320	4.567712	0.107063
H	1.768869	5.317025	0.457750
O	-1.497349	1.660466	-1.284342
O	2.640465	0.539195	-0.362534
O	0.168410	-1.905711	0.450357
H	-0.708855	-2.295975	0.457259
P	-3.026138	1.398841	-0.635984
H	-3.777452	1.512637	-1.834968
H	-3.005721	-0.015124	-0.715180

DBADP E = -1255.5109 au [M06-2X/def2-TZVP]
ZPE = 0.196561 au [M06-2X/def2-TZVP]
H_{corr} = -1255.3003 au [M06-2X/def2-TZVP]
G_{corr} = -1255.3546 au [M06-2X/def2-TZVP]

P	-0.4664280	-1.0287400	1.5477980
P	0.4664470	1.0289060	1.5476820
C	1.7279410	-0.8794730	-0.0225270
C	2.5501450	1.3583020	-0.3611330
H	2.5141690	2.4158980	-0.1246350
C	-1.6600210	-0.4890000	0.2568710
C	1.6600250	0.4890270	0.2567990
C	-2.6715420	1.3627530	-0.9269950
H	-2.7164460	2.4220970	-1.1482950
C	-2.5501510	-1.3583400	-0.3609560
H	-2.5141720	-2.4159110	-0.1243450
C	2.6715310	-1.3628520	-0.9268820
H	2.7164330	-2.4222200	-1.1480680
C	-1.7279410	0.8794700	-0.0226010
C	-3.5381050	0.4763390	-1.5462860
H	-4.2668100	0.8550490	-2.2517100
C	3.5380850	-0.4765040	-1.5462790
H	4.2667800	-0.8552890	-2.2516720
C	3.4822290	0.8856410	-1.2742910
H	4.1645490	1.5684030	-1.7618270
C	-3.4822460	-0.8857770	-1.2741520
H	-4.1645730	-1.5685900	-1.7616060
N	-0.8396730	1.6973410	0.6642410
H	-0.9969290	2.6909920	0.6648470
N	0.8396840	-1.6972700	0.6644160
H	0.9969310	-2.6909220	0.6651130

d_{PP} = 2.259 Å

DBADP E = 1254.806629 au [IEFPCM($CHCl_3$)/PBE1PBE/6-311G++(2d,2p)]

P	0.4569090	1.0339350	1.4043910
P	-0.4568990	-1.0338740	1.4044100
C	-1.7828300	0.8786190	-0.1077900
C	-2.7864470	-1.3154520	-0.1785020
H	-2.7858340	-2.3556470	0.1274360
C	1.7696130	0.4726270	0.2583560
C	-1.7696160	-0.4726190	0.2583750
C	2.8104300	-1.3666320	-0.9179960
H	2.8205370	-2.4106880	-1.2073290
C	2.7864550	1.3154410	-0.1785410
H	2.7858590	2.3556410	0.1273790
C	-2.8104520	1.3666060	-0.9180140
H	-2.8205870	2.4106580	-1.2073610
C	1.7828220	-0.8786160	-0.1077800
C	3.8053330	-0.5077430	-1.3543910
H	4.5974320	-0.8941800	-1.9843850
C	-3.8053380	0.5076920	-1.3543960
H	-4.5974380	0.8941130	-1.9843990
C	-3.8006860	-0.8367700	-0.9944950
H	-4.5853380	-1.4984960	-1.3373170
C	3.8006990	0.8367220	-0.9945060
H	4.5853700	1.4984300	-1.3373240
N	0.7683920	-1.6868770	0.3762430
H	0.9717320	-2.6677890	0.4712220
N	-0.7684290	1.6868670	0.3762440
H	-0.9715250	2.6678700	0.4707450

H_2 E = -1.1684598 au [M06-2X/def2-TZVP]
ZPE = 0.010199 au [M06-2X/def2-TZVP]
H_{corr} = -1.154956 au [M06-2X/def2-TZVP]
G_{corr} = -1.169741 au [M06-2X/def2-TZVP]

H	0.0000000	0.0000000	0.3700820
H	0.0000000	0.0000000	-0.3700820

DBADP E = -1255.351018238 au [PWPB95-D3/def2-QZVPP]
ZPE = 0.20191268 au [PBEh-3c]

P	-0.460284	-1.029941	1.466228
P	0.460288	1.030091	1.466111
C	1.751834	-0.867622	-0.089895
C	2.652306	1.358652	-0.295322
H	2.634100	2.405858	-0.017431
C	-1.708942	-0.491159	0.238079
C	1.708933	0.491176	0.238009
C	-2.730130	1.337207	-0.965195
H	-2.765020	2.386086	-1.233204
C	-2.652318	-1.358694	-0.295148
H	-2.634108	-2.405870	-0.017143
C	2.730109	-1.337321	-0.965079
H	2.764995	-2.386229	-1.232975
C	-1.751846	0.867603	-0.089971
C	-3.651510	0.452466	-1.499257
H	-4.406049	0.824623	-2.180207
C	3.651483	-0.452638	-1.499245
H	4.406014	-0.824869	-2.180164
C	3.619877	0.897576	-1.174723
H	4.346601	1.578897	-1.595082
C	-3.619899	-0.897713	-1.174588
H	-4.346627	-1.579079	-1.594867
N	-0.818216	1.684964	0.512028
H	-0.999830	2.672984	0.549976
N	0.818210	-1.684917	0.512202
H	0.999829	-2.672931	0.550263

H_2P-NH_2 E = -398.452818268 au [PWPB95-D3/def2-QZVPP]
ZPE = 0.04466564 au [PBEh-3c]

P	-0.002375	0.018447	0.001145
H	0.005034	-0.006563	1.418137
H	1.413336	-0.007950	-0.056431
N	-0.252749	-1.705878	-0.238182
H	-1.219871	-1.938728	-0.052006
H	-0.092554	-1.942059	-1.209135

HD(**DBADP**)$_{PNcleav}$ E = -1653.819170340 au [PWPB95-D3/def2-QZVPP]
ZPE = 0.24896745 au [PBEh-3c]

P	0.896040	0.424158	-0.878768
P	-0.037874	-0.211101	1.030449
C	2.706496	0.199202	1.090137
C	1.709874	-0.086983	3.265273
H	0.828688	-0.216070	3.882291
C	0.507155	2.204380	-0.625132
C	1.569605	0.008803	1.886398
C	-1.141161	3.963945	-0.706060
H	-2.147358	4.298853	-0.923909
C	1.444378	3.128435	-0.180998
H	2.453795	2.800826	0.030857
C	3.968026	0.258510	1.681302
H	4.851434	0.405083	1.071431
C	-0.800285	2.628318	-0.910425
C	-0.202286	4.863037	-0.231290
H	-0.488553	5.895016	-0.075645
C	4.084491	0.141968	3.055920
H	5.066326	0.193998	3.508435
C	2.961625	-0.022838	3.857422
H	3.062920	-0.098667	4.931294
C	1.098236	4.454431	0.022446
H	1.835414	5.158856	0.383200
P	-3.397832	1.742495	-0.991312
H	-3.960982	1.469049	-2.262780
H	-3.575669	0.409404	-0.540293
N	-1.733391	1.711398	-1.392928
H	-1.356650	0.918706	-1.891647
N	2.502413	0.319141	-0.272702
H	3.279812	0.174303	-0.893980
N	-0.148014	-1.904803	0.844437
H	0.691069	-2.463749	0.841267
H	-0.888007	-2.346172	1.363624

3.7 Calculated ^{31}P NMR shifts for dehydrocoupled dimers of $C_6H_4PH_2(EH)$

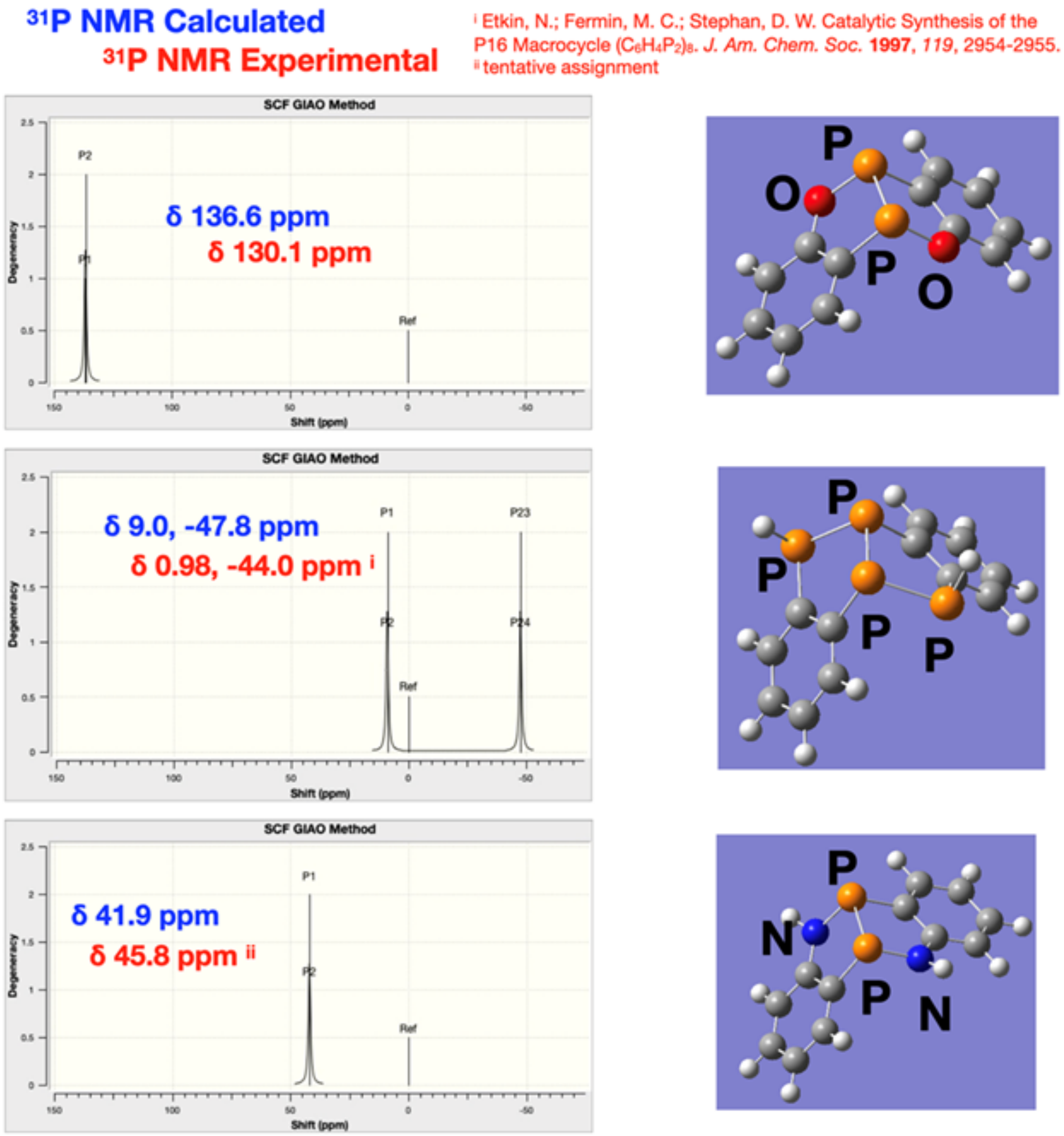

3.9 Structures and energies for proton affinities

Ph-PH_2 E = -574.31643 au [B3LYP-D3/6-311++G(2d,p)]
ZPE = 0.10778 au [B3LYP-D3/6-311++G(2d,p)]

C	-0.4872190	0.0070930	-0.0144880	H	2.1685620	2.1320260	0.0042740
C	2.3132630	-0.0115560	0.0139560	H	2.1369090	-2.1541760	0.0092760
C	0.2163750	-1.2005860	-0.0054950	H	3.3963320	-0.0202820	0.0239870
C	0.2335670	1.2030210	-0.0187040	P	-2.3313580	0.1088970	-0.0406060
C	1.6247380	1.1951360	0.0036800	H	-2.5787640	-1.1802540	-0.5771350
C	1.6054040	-1.2100900	0.0051700	H	-2.5677330	-0.3178010	1.2938220
H	-0.2970070	2.1479310	-0.0445000	H	-0.3246840	-2.1389990	-0.0053470

Ph-PH_3^+ E = -574.66002 au [B3LYP-D3/6-311++G(2d,p)]
ZPE = 0.118752 au [B3LYP-D3/6-311++G(2d,p)]

C	-0.4318800	0.0000400	-0.0207550	H	2.1817310	-2.1464380	0.0049500
C	2.3276710	-0.0000110	0.0144090	H	3.4100720	-0.0002000	0.0243890
C	0.2530840	-1.2211240	-0.0130760	P	-2.2043020	0.0000350	0.0090200
C	0.2531890	1.2210470	-0.0130120	H	-2.7319070	-1.1220790	-0.6371820
C	1.6394120	1.2103580	0.0043130	H	-2.7318950	1.1273680	-0.6279700
C	1.6391220	-1.2104150	0.0044530	H	-2.7705850	-0.0049080	1.2916890
H	-0.2791240	2.1644420	-0.0267730	H	-0.2792820	-2.1644860	-0.0270340
H	2.1819330	2.1464150	0.0046310				

DPB E = -916.29723 au [B3LYP-D3/6-311++G(2d,p)]
ZPE = 0.115886 au [B3LYP-D3/6-311++G(2d,p)]

C	0.0111880	0.7000050	0.0139450	H	1.2412150	-2.4675760	-0.0127730
C	2.4463100	-0.6943830	0.0006170	H	3.3825740	-1.2385590	-0.0029340
C	0.0172180	-0.7082390	-0.0014830	P	-1.5678750	1.6429940	-0.1107170
C	1.2309370	1.3812330	0.0125810	H	-2.1232330	1.2871970	1.1444720
C	2.4386030	0.6939310	0.0051750	H	-1.0502500	2.8787320	0.3512410
C	1.2398270	-1.3837140	-0.0032160	P	-1.4960090	-1.7691800	-0.0175720
H	1.2396840	2.4644660	0.0243800	H	-2.1269500	-1.2383860	1.1391350
H	3.3707160	1.2459760	0.0083140	H	-2.2800030	-0.9720530	-0.8932220

DPB·H⁺ E = -916.64554 au [B3LYP-D3/6-311++G(2d,p)]
ZPE = 0.126987 au [B3LYP-D3/6-311++G(2d,p)]

C	-0.0462290	0.7460560	-0.0000500	H	-3.4051960	1.2368500	-0.0001420
C	-2.4647790	-0.6914020	0.0000910	H	-1.2636380	-2.4661580	0.0002030
C	-0.0658300	-0.6592690	0.0000440	H	-3.3984000	-1.2381300	0.0001620
C	-1.2686070	1.4113630	-0.0001370	P	1.6103420	1.5645330	0.0000450
C	-2.4650550	0.7000140	-0.0000710	H	1.4024290	2.4823520	-1.0523880
C	-1.2624750	-1.3833190	0.0001280	H	1.4026590	2.4814900	1.0532830
H	-1.2891150	2.4936520	-0.0002350	P	1.4737670	-1.5687340	-0.0000840

H	2.2896810	-1.3691120	1.1163140	H	1.1480760	-2.9298590	-0.0002540
H	2.2897130	-1.3687320	-1.1163800				

PAN E = -629.70108 au [B3LYP-D3/6-311++G(2d,p)]
ZPE = 0.124866 au [B3LYP-D3/6-311++G(2d,p)]

C	0.3930960	-0.3498150	0.0023580	H	-2.0186510	2.0518570	-0.0149690
C	-2.3885700	-0.0594080	-0.0011460	H	-3.4648930	0.0650210	-0.0019480
C	-0.1799960	0.9393420	-0.0047140	P	2.2004160	-0.6764730	-0.0067210
C	-0.4508270	-1.4635550	0.0090220	H	2.5796360	0.2427750	-1.0226350
C	-1.8325530	-1.3351780	0.0067590	H	2.5920260	0.2138800	1.0345430
C	-1.5757230	1.0617440	-0.0060460	N	0.6093260	2.0798510	-0.0664430
H	-0.0042130	-2.4512870	0.0154480	H	1.5529870	1.9857090	0.2770770
H	-2.4646170	-2.2135530	0.0115870	H	0.1636410	2.9349540	0.2294090

PAN·H+ (at P) E = -630.05009 au [B3LYP-D3/6-311++G(2d,p)]
ZPE = 0.136116 au [B3LYP-D3/6-311++G(2d,p)]

C	0.2796990	-0.4121260	0.0010070	H	-3.4765440	0.3231230	-0.0018850
C	-2.4137830	0.1173790	-0.0009510	N	0.9090870	1.8857830	0.0006610
C	-0.1518480	0.9170570	0.0004000	H	0.8782030	2.4857000	0.8212410
C	-0.6119470	-1.4847350	0.0013220	H	0.8713450	2.4941200	-0.8133580
C	-1.9711490	-1.2038130	0.0004020	P	2.0489150	-0.5861410	-0.0009370
C	-1.5126780	1.1809120	-0.0008900	H	2.6943750	-0.0614330	-1.1214170
H	-0.2633100	-2.5095280	0.0019380	H	2.3460030	-1.9547170	-0.0016070
H	-2.6868380	-2.0149530	0.0005120	H	2.6981690	-0.0624080	1.1178560
H	-1.8684910	2.2036940	-0.0016000				

Table S22: Summary of energies for protonated PAN conformation isomers

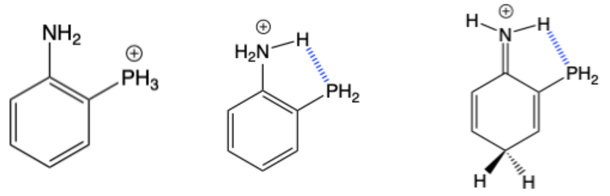

	P-Protonated	*N*-Protonated	*C*-Protonated
**H ENTHALPIES au**	-629.90507	-629.90452	-629.9013
**relative ΔH kcal/mol**	0	0.35	2.37
**G FREE ENERGIES au**	-629.94667	-629.94578	-629.94385
**relative ΔG kcal/mol**	0	0.56	1.77

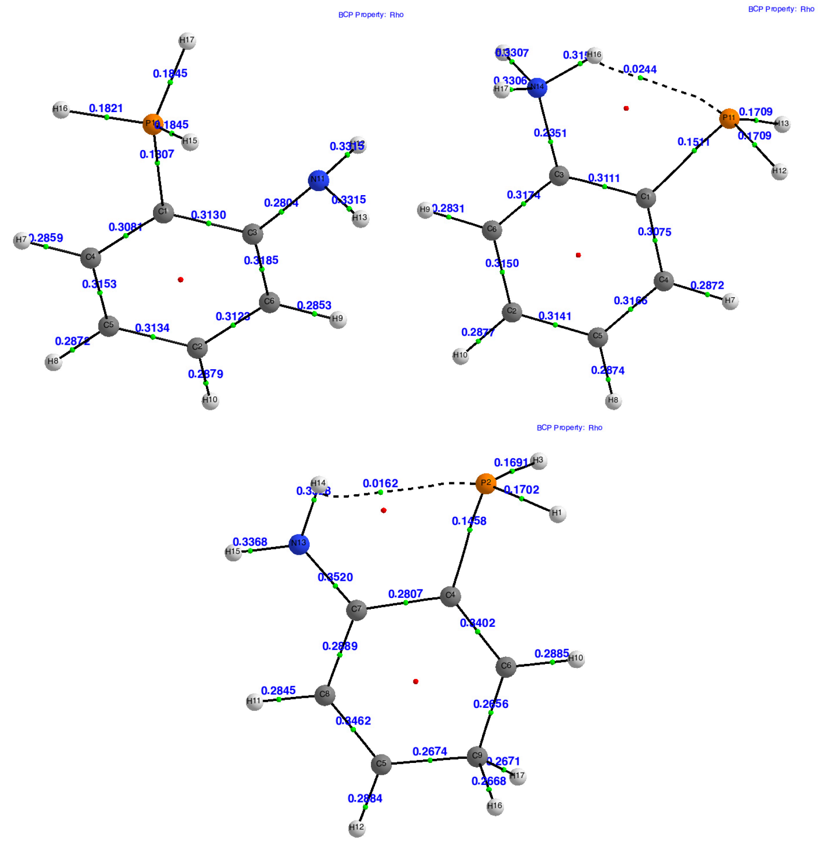

PP E = -649.57179 au [B3LYP-D3/6-311++G(2d,p)]
ZPE = 0.11244 au [B3LYP-D3/6-311++G(2d,p)]

C	0.3942440	-0.3415270	0.0000900
C	-2.3870130	-0.0446700	-0.0000720
C	-0.1893240	0.9350230	0.0000450
C	-0.4492450	-1.4571450	0.0000960
C	-1.8298150	-1.3205060	0.0000220
C	-1.5747360	1.0786690	-0.0000680
H	-0.0057200	-2.4460580	0.0001560
H	-2.4639880	-2.1974410	0.0000830
H	-1.9932140	2.0769220	-0.0001760
H	-3.4633250	0.0778490	-0.0002100
P	2.2075920	-0.6417990	-0.0000780
H	2.5808100	0.2658940	-1.0311510
H	2.5811070	0.2658200	1.0309660
O	0.5473800	2.0791670	0.0000910
H	1.4867550	1.8515970	0.0000930

PP·H$^+$ E = -649.92107 au [B3LYP-D3/6-311++G(2d,p)]
ZPE = 0.123356 au [B3LYP-D3/6-311++G(2d,p)]

C	0.3357070	-0.3617180	-0.0001110
C	-2.3987380	-0.0098710	0.0000110
C	-0.1989860	0.9346750	-0.0000510
C	-0.5020210	-1.4845640	0.0000280
C	-1.8727700	-1.3023380	0.0001510
C	-1.5751140	1.1091140	-0.0001650
H	-0.0846420	-2.4836720	-0.0000630
H	-2.5318590	-2.1592890	0.0002820
H	-1.9984330	2.1065110	-0.0003610
H	-3.4721830	0.1295240	-0.0000170
P	2.0966360	-0.5015460	-0.0000080
H	2.7143690	0.0835520	-1.1109510
H	2.4524040	-1.8520870	-0.0043300
H	2.7142230	0.0758930	1.1150900
O	0.7157120	1.9374990	-0.0000370
H	0.3024190	2.8109810	0.0015910

Cy-PH_2 E = -577.95606 au [B3LYP-D3/6-311++G(2d,p)]
ZPE = 0.177581 au [B3LYP-D3/6-311++G(2d,p)]

H	0.3998430	2.1541150	-0.2572000
C	-0.0857630	1.2631900	0.1487530
C	-2.2766530	0.0058880	0.3161710
C	-0.0933220	-1.2648520	0.1407100
C	-1.5863190	-1.2575800	-0.2033480
C	0.6111700	0.0012250	-0.3709430
C	-1.5795590	1.2687800	-0.1958280
H	-2.2552600	0.0025870	1.4125020
H	0.0231530	-1.3214370	1.2300370
H	-1.7028340	-1.3111060	-1.2921550
H	0.5541810	-0.0017620	-1.4669970
H	-1.6975080	1.3302340	-1.2841560
H	0.0355900	1.3101290	1.2372170
H	-3.3303480	0.0088610	0.0241230
H	0.3861680	-2.1566610	-0.2713320
H	-2.0667140	-2.1502410	0.2067860
H	-2.0544590	2.1616600	0.2204560
P	2.4432660	-0.1026530	0.0095750
H	2.7764290	1.2392400	-0.3161300
H	2.3454560	0.1742720	1.4001290

Cy-PH_3^+ E = -578.30558 au [B3LYP-D3/6-311++G(2d,p)]
ZPE = 0.188505 au [B3LYP-D3/6-311++G(2d,p)]

H	-0.8397390	2.7501790	0.0000000
P	0.4723110	2.2654630	0.0000000
H	1.0934070	2.8281090	1.1209800
H	1.0934070	2.8281090	-1.1209800
C	0.5021600	0.4510380	0.0000000
C	-0.1675200	-1.6157810	1.2641290
C	-0.1675200	-1.6157810	-1.2641290
C	-0.8308690	-2.1647880	0.0000000
C	-0.1675200	-0.0815050	-1.2831470
C	-0.1675200	-0.0815050	1.2831470
H	0.8651290	-1.9739340	1.3278850
H	0.8651290	-1.9739340	-1.3278850
H	-1.8939310	-1.9005200	0.0000000
H	-1.2005800	0.2800480	-1.3314800
H	-1.2005800	0.2800480	1.3314800
H	1.5656920	0.1851720	0.0000000
H	-0.6798710	-1.9766040	2.1573470
H	-0.6798710	-1.9766040	-2.1573470
H	-0.7822080	-3.2546480	0.0000000
H	0.3510380	0.2862810	-2.1718950
H	0.3510380	0.2862810	2.1718950

Ph_2PH E = -805.45938 au [B3LYP-D3/6-311++G(2d,p)]
ZPE = 0.190832 au [B3LYP-D3/6-311++G(2d,p)]

C	-1.4158660	0.6303700	-0.0210200
C	-3.6838140	-0.9975100	0.2111360
C	-1.8341950	0.1633010	1.2288570
C	-2.1563980	0.2762200	-1.1508340
C	-3.2864550	-0.5270830	-1.0350380
C	-2.9525200	-0.6516950	1.3438870
H	-1.2776440	0.4386770	2.1176990
H	-1.8452670	0.6241980	-2.1284370
H	-3.8511120	-0.7916070	-1.9210480
H	-3.2588130	-1.0115480	2.3187160
H	-4.5602090	-1.6275860	0.3009150
P	0.0678670	1.7093750	-0.0983870
H	0.0348910	1.9195930	-1.5002560
C	1.4410870	0.4683590	-0.0726030
C	3.6174220	-1.2900990	0.0032450
C	1.2736950	-0.8804090	-0.3927670
C	2.7091080	0.9205480	0.2993100
C	3.7933530	0.0499390	0.3281880
C	2.3543020	-1.7531900	-0.3530030
H	0.2948710	-1.2516630	-0.6682570
H	2.8470160	1.9605410	0.5732790
H	4.7715790	0.4165620	0.6152030
H	2.2101890	-2.7978560	-0.6020430
H	4.4581760	-1.9724380	0.0338890

$Ph_2PH_2^+$ E = -805.82805 au [B3LYP-D3/6-311++G(2d,p)]
ZPE = 0.201847 au [B3LYP-D3/6-311++G(2d,p)]

C	0.0000000	-1.4958990	0.4514380	H	-1.1020130	-0.0785380	2.2774500
C	0.0430160	-3.8413980	-1.0138200	C	0.0000000	1.4958990	0.4514380
C	0.8434630	-1.5981750	-0.6608270	C	-0.0430160	3.8413980	-1.0138200
C	-0.8240250	-2.5606300	0.8298840	C	0.8240250	2.5606300	0.8298840
C	-0.7973750	-3.7342730	0.0896790	C	-0.8434630	1.5981750	-0.6608270
C	0.8604650	-2.7772900	-1.3890330	C	-0.8604650	2.7772900	-1.3890330
H	1.4707480	-0.7669120	-0.9583170	C	0.7973750	3.7342730	0.0896790
H	-1.4819710	-2.4777010	1.6864410	H	1.4819710	2.4777010	1.6864410
H	-1.4347720	-4.5617420	0.3720500	H	-1.4707480	0.7669120	-0.9583170
H	1.5086650	-2.8670390	-2.2507750	H	-1.5086650	2.8670390	-2.2507750
H	0.0588580	-4.7583170	-1.5890340	H	1.4347720	4.5617420	0.3720500
P	0.0000000	0.0000000	1.4157670	H	-0.0588580	4.7583170	-1.5890340
H	1.1020130	0.0785380	2.2774500				

Cy_2PH E = -812.73334 au [B3LYP-D3/6-311++G(2d,p)]
ZPE = 0.330101 au [B3LYP-D3/6-311++G(2d,p)]

H	-2.0581790	-2.1364380	0.7073700	H	-0.0512230	-2.4056470	-0.6247950
C	-2.4270600	-1.2318870	0.2172590	C	1.6427070	-0.6144390	-0.6286020
C	-3.9719640	0.7613290	0.4839230	C	3.3055850	1.2918900	-0.4500520
C	-1.7682680	1.0120320	-0.7268590	C	3.3839290	-0.6054340	1.2162690
C	-2.8042600	1.6982960	0.1681210	C	3.6215710	0.8900510	0.9927230
C	-1.2530320	-0.2954630	-0.1043480	C	1.9616090	-1.0167680	0.8165410
C	-3.4763500	-0.5487030	1.1005900	C	1.8808660	0.8876370	-0.8415230
H	-4.5149790	0.5406610	-0.4427440	H	4.0165240	0.8028480	-1.1262150
H	-2.2215610	0.7833900	-1.6981390	H	4.1029800	-1.1771880	0.6178220
H	-2.3252290	2.0078990	1.1047910	H	2.9794770	1.4622480	1.6733040
H	-0.7413310	-0.0579860	0.8337770	H	1.2488490	-0.5369660	1.4958430
H	-3.0342100	-0.3393040	2.0819110	H	1.1743720	1.4546300	-0.2255830
H	-2.8974710	-1.5541290	-0.7199730	H	2.3363840	-1.1536710	-1.2870520
H	-4.6834340	1.2505620	1.1550110	H	3.4377410	2.3695220	-0.5823220
H	-0.9383820	1.6925350	-0.9298950	H	3.5687910	-0.8683580	2.2618100
H	-3.1673870	2.6105520	-0.3136840	H	4.6533870	1.1518210	1.2428420
H	-4.3150860	-1.2288460	1.2748090	H	1.8337490	-2.0950950	0.9400580
P	-0.0436610	-1.1402190	-1.2681240	H	1.6791260	1.1589920	-1.8813480

$Cy_2PH_2^+$ E = -813.11327 au [B3LYP-D3/6-311++G(2d,p)]
ZPE = 0.341271 au [B3LYP-D3/6-311++G(2d,p)]

H	1.6238560	-1.0714470	1.9817380	H	4.1629660	-0.2591500	-1.2532840
C	1.8843610	-0.3108920	1.2403010	H	2.2563870	-1.7447740	-0.3321360
C	3.7613700	1.1356950	0.3471690	H	3.9887060	-0.7688300	1.3426640
C	2.0533620	0.1865320	-1.2664160	H	1.2338650	0.5513410	1.4174370
C	3.5149640	0.6037340	-1.0662420	H	4.8147790	1.3928330	0.4696650
C	1.6538300	-0.8390800	-0.1887440	H	1.9127720	-0.2353740	-2.2645890
C	3.3471710	0.1160160	1.4099080	H	3.7753530	1.3573710	-1.8115150
H	3.1958230	2.0630790	0.4916870	H	3.4843850	0.5266100	2.4118160
H	1.4127870	1.0691560	-1.1957880	P	-0.0573810	-1.4325990	-0.3891580

H	-0.1686420	-2.6810900	0.2362150
H	-0.2950270	-1.7011370	-1.7435210
C	-1.4132730	-0.3924190	0.2325680
C	-2.6135450	1.8202790	0.0981750
C	-3.9158880	-0.2952060	0.5461740
C	-3.9401040	1.0878050	-0.1059400
C	-2.7472330	-1.1409900	0.0275810
C	-1.4242780	1.0024540	-0.4194440
H	-2.4675240	2.0263100	1.1639770
H	-3.8285580	-0.1905290	1.6327280
H	-4.1385330	0.9825430	-1.1782510
H	-2.8916880	-1.3381770	-1.0403700
H	-1.4999000	0.8950280	-1.5070990
H	-1.2216510	-0.2893050	1.3066700
H	-2.6251580	2.7871190	-0.4082150
H	-4.8495630	-0.8276610	0.3567330
H	-4.7595390	1.6796930	0.3051470
H	-2.7269900	-2.1083170	0.5359370
H	-0.4926180	1.5301210	-0.2108180

Me_2P-PMe_2 E = -842.51852 au [B3LYP-D3/6-311++G(2d,p)]
ZPE = 0.151281 au [B3LYP-D3/6-311++G(2d,p)]

P	0.4783290	1.0124090	-0.0002510
P	-0.4783290	-1.0124090	-0.0002510
C	0.4783290	-1.7494320	1.4173150
H	0.1236980	-1.3258360	2.3577770
H	1.5528820	-1.5772440	1.3286080
H	0.2893750	-2.8244570	1.4440270
C	0.4792190	-1.7499480	-1.4169310
H	0.2891500	-2.8247530	-1.4443810
H	1.5538270	-1.5788340	-1.3269240
H	0.1261610	-1.3255590	-2.3576440
C	-0.4783290	1.7494320	1.4173150
H	-0.1236980	1.3258360	2.3577770
H	-1.5528820	1.5772440	1.3286080
H	-0.2893750	2.8244570	1.4440270
C	-0.4792190	1.7499480	-1.4169310
H	-0.1261610	1.3255590	-2.3576440
H	-0.2891500	2.8247530	-1.4443810
H	-1.5538270	1.5788340	-1.3269240

$Me_2P-P(H)Me_2^+$ E = -842.89323 au [B3LYP-D3/6-311++G(2d,p)]
ZPE = 0.161975 au [B3LYP-D3/6-311++G(2d,p)]

P	1.1203410	-0.5116300	-0.5161140
P	-0.9494150	0.2642530	-0.3704400
H	-1.1803030	0.9621100	-1.5635710
C	2.0158560	1.1078280	-0.4169740
H	1.8186850	1.7015350	-1.3100350
H	3.0812020	0.8682860	-0.4129160
H	1.7886930	1.6850970	0.4787990
C	1.2858290	-1.1423650	1.2200320
H	1.0915350	-0.3916420	1.9856100
H	2.3190910	-1.4802560	1.3230960
H	0.6452990	-2.0115830	1.3697050
C	-1.3230370	1.4178800	0.9782720
H	-2.3578470	1.7527450	0.8935350
H	-0.6598760	2.2804000	0.9218740
H	-1.1846700	0.9144760	1.9350750
C	-2.1217570	-1.1150080	-0.3374410
H	-3.1413440	-0.7343090	-0.4012090
H	-2.0018030	-1.6681730	0.5944610
H	-1.9239040	-1.7780410	-1.1794460

Ph_2P-PPh_2 E = -1609.748853 au [B3LYP-D3/6-311++G(2d,p)]
ZPE = 0.151281 au [B3LYP-D3/6-311++G(2d,p)]

H	-2.8033280	0.6903410	0.6444450
C	-2.1957980	1.0037880	1.4837790
C	-0.6362930	1.7924520	3.6480220
C	-0.8494490	1.3194450	1.2849110
C	-2.7555450	1.0844280	2.7522950
C	-1.9763370	1.4756920	3.8378420
C	-0.0777610	1.7186880	2.3776060
H	-3.8001980	0.8350740	2.8959630
H	-2.4138240	1.5332590	4.8272290
H	0.9697940	1.9530530	2.2334490
H	-0.0234770	2.0939240	4.4886860
P	0.0032210	1.1309140	-0.3351340
P	-0.0032210	-1.1309140	-0.3351340
C	1.3628230	-1.4939690	-1.5172870
C	3.3130250	-2.2432910	-3.3882730
C	1.9763370	-2.7505980	-1.4483110
C	1.7379710	-0.6243480	-2.5425900
C	2.7039640	-0.9977690	-3.4723900

C	2.9464830	-3.1190820	-2.3694130
H	1.6978410	-3.4408850	-0.6603620
H	1.2909820	0.3589020	-2.6180830
H	2.9843030	-0.3069320	-4.2584880
H	3.4167700	-4.0922200	-2.2938650
H	4.0683720	-2.5312870	-4.1091110
C	0.8494490	-1.3194450	1.2849110
C	1.9763370	-1.4756920	3.8378420
C	0.0777610	-1.7186880	2.3776060
C	2.1957980	-1.0037880	1.4837790
C	2.7555450	-1.0844280	2.7522950
C	0.6362930	-1.7924520	3.6480220
H	-0.9697940	-1.9530530	2.2334490
H	2.8033280	-0.6903410	0.6444450
H	3.8001980	-0.8350740	2.8959630
H	0.0234770	-2.0939240	4.4886860
H	2.4138240	-1.5332590	4.8272290
C	-1.3628230	1.4939690	-1.5172870
C	-3.3130250	2.2432910	-3.3882730
C	-1.7379710	0.6243480	-2.5425900
C	-1.9763370	2.7505980	-1.4483110
C	-2.9464830	3.1190820	-2.3694130
C	-2.7039640	0.9977690	-3.4723900
H	-1.2909820	-0.3589020	-2.6180830
H	-1.6978410	3.4408850	-0.6603620
H	-3.4167700	4.0922200	-2.2938650
H	-2.9843030	0.3069320	-4.2584880
H	-4.0683720	2.5312870	-4.1091110

Ph$_2$P-P(H)Ph$_2^+$ E = -1610.135518 au [B3LYP-D3/6-311++G(2d,p)]
ZPE = 0.376622 au [B3LYP-D3/6-311++G(2d,p)]

H	-0.8961180	-2.3913860	1.3420250
C	-1.7320460	-2.1346230	0.7036710
C	-3.8927720	-1.4981470	-0.9364850
C	-1.5130740	-1.4913750	-0.5196530
C	-3.0225900	-2.4569530	1.0977150
C	-4.1042590	-2.1356150	0.2797630
C	-2.6005320	-1.1780650	-1.3377570
H	-3.1870970	-2.9598730	2.0424730
H	-5.1098260	-2.3903770	0.5899700
H	-2.4370530	-0.6805460	-2.2860300
H	-4.7307750	-1.2545550	-1.5768630
P	0.1328240	-0.9200750	-1.0919610
C	1.7170460	1.7222670	0.1933070
C	3.9424040	3.3373360	-0.1692160
C	2.3181450	2.3329480	1.2977800
C	2.2299890	1.9180610	-1.0922080
C	3.3424320	2.7302630	-1.2678900
C	3.4325280	3.1381720	1.1108880
H	1.9231060	2.1814260	2.2950100
H	1.7730960	1.4336230	-1.9462820
H	3.7439390	2.8832100	-2.2611740
H	3.9042600	3.6086120	1.9637630
H	4.8132040	3.9649250	-0.3097040
C	-1.2412800	1.6929850	0.3529510
C	-3.6096180	3.1136860	0.1158550
C	-2.3511850	1.3222030	1.1172340
C	-1.3150080	2.7729230	-0.5302640
C	-2.5037230	3.4801710	-0.6451710
C	-3.5319510	2.0394420	0.9972730
H	-2.3001580	0.4774770	1.7913980
H	-0.4519180	3.0675410	-1.1134440
H	-2.5647860	4.3204590	-1.3244620
H	-4.3918260	1.7588190	1.5913710
H	-4.5336100	3.6705110	0.0253680
C	1.3273780	-2.1116440	-0.3679740
C	3.2099550	-3.9939140	0.4802160
C	2.6004700	-1.7034560	0.0423630
C	1.0147680	-3.4745690	-0.3715960
C	1.9543420	-4.4073580	0.0496630
C	3.5303040	-2.6409640	0.4740630
H	2.8830960	-0.6590820	0.0197620
H	0.0416760	-3.8128770	-0.7025860
H	1.7014150	-5.4600120	0.0411270
H	4.5093690	-2.3122380	0.7989480
H	3.9377220	-4.7230490	0.8123650
P	0.2561630	0.7098270	0.4274480
H	0.3271370	0.1884660	1.7274980

BQ E = -381.59349 au [B3LYP-D3/6-311++G(2d,p)]
ZPE = 0.084777 au [B3LYP-D3/6-311++G(2d,p)]

O	0.0000000	0.0000000	2.6557990
C	0.0000000	0.0000000	1.4367550
C	0.0000000	-1.2665160	0.6680350
H	0.0000000	-2.1773950	1.2545340
C	0.0000000	-1.2665160	-0.6680350
H	0.0000000	-2.1773950	-1.2545340
C	0.0000000	0.0000000	-1.4367550
C	0.0000000	1.2665160	-0.6680350

H 0.0000000 2.1773950 -1.2545340
C 0.0000000 1.2665160 0.6680350
H 0.0000000 2.1773950 1.2545340
O 0.0000000 0.0000000 -2.6557990

[BQ·H]⁺ E = -381.91151 au [B3LYP-D3/6-311++G(2d,p)]
ZPE = 0.097406 au [B3LYP-D3/6-311++G(2d,p)]

O -2.6957480 -0.0216740 0.0003190
C -1.4864150 -0.0057310 -0.0000710
C -0.7284410 1.2746000 -0.0002000
H -1.3137420 2.1861100 -0.0003090
C 0.6150530 1.2743690 -0.0000570
H 1.2102010 2.1789060 -0.0000260
C 1.3110260 0.0158620 0.0000390
C 0.6363900 -1.2558060 -0.0000850
H 1.2273770 -2.1655080 -0.0000830
C -0.7068640 -1.2770090 -0.0001930
H -1.2787710 -2.1971540 -0.0002700
O 2.6024070 0.0972290 0.0001490
H 3.0571670 -0.7644910 0.0003460

tBuBQ E = -696.24922 au [B3LYP-D3/6-311++G(2d,p)]
ZPE = 0.309597 au [B3LYP-D3/6-311++G(2d,p)]

O -2.6248440 -0.3340690 0.0000000
C -1.4188090 -0.1389820 0.0000000
C -0.5124440 -1.3062280 0.0000000
H -1.0411010 -2.2494410 0.0000000
C 0.8265260 -1.2365490 0.0000000
C 1.4188090 0.1389820 0.0000000
C 0.5124440 1.3062280 0.0000000
H 1.0411010 2.2494410 0.0000000
C -0.8265260 1.2365490 0.0000000
O 2.6248440 0.3340690 0.0000000
C -1.7384820 2.4566130 0.0000000
C -2.6248440 2.4389260 1.2645370
H -3.2664260 1.5613820 1.2888950
H -3.2553110 3.3309660 1.2766620
H -2.0095130 2.4500620 2.1673920
C -0.9283030 3.7600890 0.0000000
H -0.2955590 3.8469690 -0.8859650
H -0.2955590 3.8469690 0.8859650
H -1.6155140 4.6077010 0.0000000
C -2.6248440 2.4389260 -1.2645370
H -3.2664260 1.5613820 -1.2888950
H -2.0095130 2.4500620 -2.1673920
H -3.2553110 3.3309660 -1.2766620
C 1.7384820 -2.4566130 0.0000000
C 0.9283030 -3.7600890 0.0000000
H 0.2955590 -3.8469690 -0.8859650
H 0.2955590 -3.8469690 0.8859650
H 1.6155140 -4.6077010 0.0000000
C 2.6248440 -2.4389260 1.2645370
H 3.2664260 -1.5613820 1.2888950
H 3.2553110 -3.3309660 1.2766620
H 2.0095130 -2.4500620 2.1673920
C 2.6248440 -2.4389260 -1.2645370
H 3.2664260 -1.5613820 -1.2888950
H 2.0095130 -2.4500620 -2.1673920
H 3.2553110 -3.3309660 -1.2766620

[tBuBQ·H]⁺ E = -696.58687 au [B3LYP-D3/6-311++G(2d,p)]
ZPE = 0.321923 au [B3LYP-D3/6-311++G(2d,p)]

O 1.9756830 -1.7188350 0.0000000
C 0.9852530 -0.8728630 0.0000000
C 1.2839790 0.5214310 0.0000000
H 2.3325860 0.7942900 0.0000000
C 0.3226110 1.4770010 0.0000000
C -1.1012380 0.9606800 0.0000000
C -1.3361210 -0.5026180 0.0000000
H -2.3825170 -0.7710900 0.0000000
C -0.3608310 -1.4331330 0.0000000
O -2.0558900 1.7060190 0.0000000
C -0.6219580 -2.9395680 0.0000000
C -0.0186590 -3.5779370 1.2715970
H 1.0657410 -3.4998760 1.3087350
H -0.2736750 -4.6381630 1.2869900
H -0.4348150 -3.1229530 2.1726740
C -2.1326010 -3.2150920 0.0000000
H -2.6244100 -2.8127050 -0.8881060
H -2.6244100 -2.8127050 0.8881060
H -2.2924000 -4.2928630 0.0000000
C -0.0186590 -3.5779370 -1.2715970
H 1.0657410 -3.4998760 -1.3087350
H -0.4348150 -3.1229530 -2.1726740

H	-0.2736750	-4.6381630	-1.2869900
C	0.6087430	2.9522940	0.0000000
C	2.1158900	3.2388610	0.0000000
H	2.6105080	2.8423830	-0.8898870
H	2.6105080	2.8423830	0.8898870
H	2.2701480	4.3173080	0.0000000
C	-0.0186590	3.5879770	1.2709340
H	-1.1006160	3.4895370	1.2806730
H	0.2335620	4.6489890	1.2786740
H	0.3915140	3.1390050	2.1776130
C	-0.0186590	3.5879770	-1.2709340
H	-1.1006160	3.4895370	-1.2806730
H	0.3915140	3.1390050	-2.1776130
H	0.2335620	4.6489890	-1.2786740
H	2.8436910	-1.2799940	0.0000000

DQI E = -961.43594 au [B3LYP-D3/6-311++G(2d,p)]
ZPE = 0.380606 au [B3LYP-D3/6-311++G(2d,p)]

C	-1.1584280	0.8472260	-0.9263970
C	0.0621260	1.4243240	-0.9212170
C	1.2614940	0.6015650	-0.6462600
C	1.1584240	-0.8471600	-0.9264210
C	-0.0621400	-1.4242540	-0.9212800
C	-1.2614940	-0.6015060	-0.6463030
N	2.3016880	1.2262340	-0.2154570
N	-2.3017310	-1.2262150	-0.2156200
C	3.4271970	0.6128630	0.3295910
C	5.7303060	-0.4701710	1.4997720
C	3.3263430	-0.2962010	1.3913010
C	4.6986390	1.0043320	-0.1083570
C	5.8358190	0.4481000	0.4575740
C	4.4705150	-0.8262900	1.9706980
H	2.3451310	-0.5680470	1.7600390
H	4.7729030	1.7339670	-0.9049370
H	6.8125350	0.7421350	0.0923030
H	4.3771310	-1.5232220	2.7951740
H	6.6208330	-0.8902650	1.9499110
C	-3.4272110	-0.6128600	0.3295130
C	-5.7302810	0.4700730	1.4998660
C	-4.6986710	-1.0043520	-0.1083640
C	-3.3263180	0.2961820	1.3912400
C	-4.4704700	0.8262170	1.9707230
C	-5.8358350	-0.4481710	0.4576500
H	-4.7729600	-1.7339750	-0.9049530
H	-2.3450950	0.5680360	1.7599430
H	-4.3770580	1.5231230	2.7952190
H	-6.8125640	-0.7422300	0.0924350
H	-6.6207910	0.8901320	1.9500740
C	0.2521180	2.8976330	-1.1485060
H	1.2276790	3.2090620	-0.7847050
H	-0.5198760	3.4850560	-0.6501140
H	0.1980670	3.1284810	-2.2180800
C	-2.3878690	1.6411960	-1.2907190
H	-2.7580000	2.2457520	-0.4597350
H	-3.2050000	0.9941080	-1.6003300
H	-2.1572190	2.3174220	-2.1153070
C	2.3878510	-1.6411740	-1.2906770
H	2.1572390	-2.3173350	-2.1153320
H	2.7578330	-2.2458240	-0.4596910
H	3.2050730	-0.9941450	-1.6001530
C	-0.2520920	-2.8975650	-1.1485980
H	0.5196580	-3.4849930	-0.6498280
H	-0.1975300	-3.1284550	-2.2181360
H	-1.2278240	-3.2089550	-0.7852270

[DQI·H]⁺ E = -961.83427 au [B3LYP-D3/6-311++G(2d,p)]
ZPE = 0.394959 au [B3LYP-D3/6-311++G(2d,p)]

C	-1.1742680	0.8974350	-0.9252480
C	0.0818270	1.4183890	-0.8983580
C	1.2095820	0.5298520	-0.6738710
C	1.0885690	-0.8855800	-0.9089570
C	-0.1695020	-1.4174850	-0.9509800
C	-1.3324560	-0.5508920	-0.7113640
N	-2.4102460	-1.1639470	-0.3357240
C	3.5096810	0.5268900	0.2925140
C	5.7510240	-0.4931200	1.5585070
C	3.3535050	-0.3491500	1.3633570
C	4.7760900	0.9176990	-0.1334890
C	5.8965360	0.4012830	0.5021880
C	4.4804140	-0.8622530	1.9893960
H	2.3617040	-0.6112220	1.7084450
H	4.8809100	1.6038300	-0.9652350
H	6.8835930	0.6947580	0.1696060
H	4.3657640	-1.5406940	2.8248620
H	6.6268270	-0.8933140	2.0523810
C	-3.4886190	-0.5741660	0.2863690
C	-5.7364790	0.4664350	1.5660120

C	-4.7659220	-1.0963730	0.0127790
C	-3.3487680	0.4336310	1.2595160
C	-4.4670590	0.9370850	1.8986420
C	-5.8808820	-0.5486940	0.6203380
H	-4.8541530	-1.9054520	-0.7002730
H	-2.3580970	0.7696060	1.5375200
H	-4.3546320	1.6914660	2.6670490
H	-6.8656930	-0.9272370	0.3788710
H	-6.6090700	0.8689920	2.0640810
N	2.3663280	1.1051010	-0.3337160
H	2.3846700	2.1154130	-0.3772470
C	-0.3875320	-2.8816330	-1.1747180
H	0.2054220	-3.4830310	-0.4825740
H	-0.0659230	-3.1517050	-2.1852540
H	-1.4343810	-3.1404380	-1.0596850
C	2.2925530	-1.7463410	-1.1982820
H	2.6036980	-2.3378630	-0.3350270
H	3.1497400	-1.1581020	-1.5151760
H	2.0526800	-2.4417680	-2.0016720
C	0.3553510	2.8915470	-1.0549100
H	1.1200140	3.0835480	-1.8135150
H	0.6904670	3.3397350	-0.1131140
H	-0.5358560	3.4332910	-1.3538720
C	-2.3650570	1.7386330	-1.3105910
H	-3.2437410	1.1286020	-1.4958970
H	-2.1375230	2.2864240	-2.2268650
H	-2.6305330	2.4679330	-0.5444420

3.10 Structures and energies for proposed mechanism

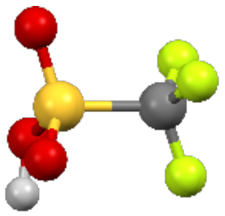

HOTf E = -962.101311418 au [CPCM$_{CH2Cl2}$/PWPB95-D3/def2-QZVPP]
ZPE = 0.03973617 au [CPCM$_{CH2Cl2}$/PBEh-3c]
G_{corr} = 0.00705624 au [CPCM$_{CH2Cl2}$/PBEh-3c]

H	-1.548892	-2.015713	-1.399196	C	-2.489751	-4.617194	-0.029552
S	-1.300142	-4.161250	-1.363366	F	-3.698027	-4.763757	-0.536235
O	-0.023488	-3.953242	-0.732342	F	-2.504769	-3.672157	0.893342
O	-1.932254	-2.792153	-1.843147	F	-2.090072	-5.755213	0.506879
O	-1.496750	-5.102015	-2.429904				

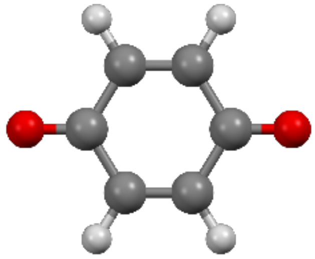

BQ E = -381.399971512 au [CPCM$_{CH2Cl2}$/PWPB95-D3/def2-QZVPP]
ZPE = 0.08801525 au [CPCM$_{CH2Cl2}$/PBEh-3c]
G_{corr} = 0.05770258 au [CPCM$_{CH2Cl2}$/PBEh-3c]

C	-0.074130	0.000072	0.031042	O	1.188352	0.000652	3.345891
C	-0.074179	0.000643	1.364108	O	1.188618	-0.001323	-1.950607
C	1.188426	-0.000042	2.132972	H	-0.996448	0.001633	1.931395
C	2.451100	-0.000989	1.364242	H	-0.996318	0.000599	-0.536361
C	2.451149	-0.001559	0.031176	H	3.373288	-0.001189	1.931645
C	1.188543	-0.001412	-0.737688	H	3.373418	-0.002224	-0.536112

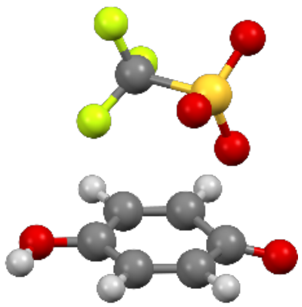

[BQ-H]OTf E = -1343.496757732 au [$CPCM_{CH2Cl2}$/PWPB95-D3/def2-QZVPP]
ZPE = 0.12962932 au [$CPCM_{CH2Cl2}$/PBEh-3c]
G_{corr} = 0.08975218 au [$CPCM_{CH2Cl2}$/PBEh-3c]

C	-0.015064	0.233937	0.222142
C	0.166982	0.213032	1.546334
C	1.504332	0.107418	2.066625
C	2.661024	0.090372	1.222311
C	2.491244	0.119268	-0.106298
C	1.130646	0.057369	-0.703065
O	1.602853	0.043651	3.339461
O	0.965266	-0.222158	-1.863006
H	-0.653076	0.283118	2.247929
H	-1.002828	0.311830	-0.211834
H	3.646213	0.081066	1.671876
H	3.334797	0.114464	-0.783325
H	2.518644	-0.018875	3.660826
S	1.689929	3.880381	-0.175903
O	0.959859	4.930036	-0.878405
O	1.448432	2.525163	-0.698578
O	3.083169	4.170014	0.145868
C	0.879657	3.840729	1.477666
F	1.492623	2.965635	2.284338
F	-0.398033	3.481585	1.381963
F	0.935139	5.034154	2.059555

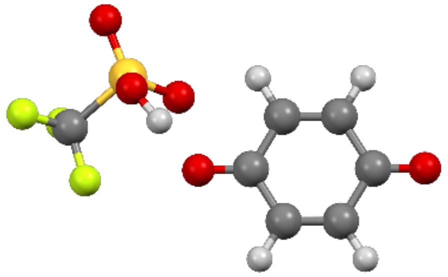

BQ···HOTf E = -1343.514585717 au [$CPCM_{CH2Cl2}$/PWPB95-D3/def2-QZVPP]
ZPE = 0.12881812 au [$CPCM_{CH2Cl2}$/PBEh-3c]
G_{corr} = 0.08688279 au [$CPCM_{CH2Cl2}$/PBEh-3c]

C	0.022699	-0.051724	0.265056
C	0.234099	-0.835882	1.322035
C	1.535382	-0.845636	2.004563
C	2.601460	0.030746	1.498949
C	2.391392	0.813685	0.440840
C	1.086517	0.833674	-0.257527
O	1.705612	-1.582369	2.969599
O	0.898875	1.545587	-1.218230
H	-0.534874	-1.488109	1.715178
H	-0.931195	-0.035316	-0.246177
H	3.557521	0.019120	2.004803
H	3.168455	1.461117	0.055392
H	2.964720	-1.663407	3.790665
S	3.999527	-0.480044	5.314139
O	3.323443	0.685611	4.795060
O	3.800837	-1.714547	4.383113
O	5.380898	-0.436816	5.713312
C	3.045576	-1.009593	6.799067
F	3.560124	-2.118306	7.297736
F	1.782759	-1.220106	6.465816
F	3.107203	-0.048021	7.703859

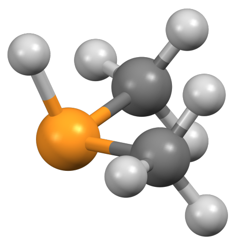

Me_2PH E = -421.714371017 au [$CPCM_{CH2Cl2}$/PWPB95-D3/def2-QZVPP]
ZPE = 0.08632266 au [$CPCM_{CH2Cl2}$/PBEh-3c]

G_{corr} = 0.05917721 au [CPCM$_{CH2Cl2}$/PBEh-3c]

P	-2.026323	-1.047064	6.960652	C	-2.550150	0.115528	8.300091
C	-0.205849	-0.723426	6.926439	H	-2.493885	1.138574	7.928603
H	0.289048	-1.466716	6.302146	H	-3.586497	-0.080028	8.573784
H	-0.024215	0.255114	6.482286	H	-1.929816	0.035242	9.192403
H	0.243077	-0.743081	7.919188	H	-1.995496	-2.230850	7.735795

(*b*)‡

E = -1765.205668472 au [CPCM$_{CH2Cl2}$/PWPB95-D3/def2-QZVPP]
ZPE = 0.21176634 au [CPCM$_{CH2Cl2}$/PBEh-3c]
G_{corr} = 0.16483069 au [CPCM$_{CH2Cl2}$/PBEh-3c]
υ = -1200.58 cm^{-1} [CPCM$_{CH2Cl2}$/PBEh-3c]

C	0.997503	0.270873	-1.217647	O	3.102212	0.049887	6.108234
C	0.495360	0.874570	-0.111941	C	2.288250	2.515123	5.817207
C	1.029709	0.575988	1.175715	F	1.368879	2.360219	6.762951
C	2.094483	-0.362191	1.300523	F	1.828570	3.381950	4.919772
C	2.594218	-0.967159	0.191656	F	3.383378	3.023497	6.371564
C	2.091645	-0.645432	-1.108086	P	1.458013	-3.519730	-2.778410
O	0.506832	1.179287	2.185938	C	0.088568	-3.038089	-1.698345
O	2.661644	-1.125256	-2.155838	H	-0.706588	-2.549898	-2.256797
H	-0.318489	1.583423	-0.183361	H	-0.314410	-3.900542	-1.164190
H	0.594056	0.488255	-2.198358	H	0.444471	-2.342465	-0.915996
H	2.508508	-0.583611	2.273365	C	2.526740	-4.398936	-1.590484
H	3.416957	-1.665880	0.274024	H	2.069256	-5.359914	-1.344906
H	0.895613	0.916913	3.096314	H	3.506327	-4.585630	-2.025147
S	2.673872	0.905482	5.014572	H	2.641471	-3.845380	-0.654770
O	3.662211	1.239754	3.997779	H	2.171894	-2.040532	-2.72474
O	1.349492	0.510935	4.451957				

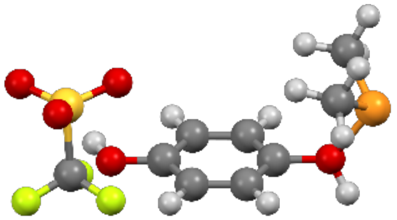

I

E = -1765.273948525 au [CPCM$_{CH2Cl2}$/PWPB95-D3/def2-QZVPP]
ZPE = 0.22038955 au [CPCM$_{CH2Cl2}$/PBEh-3c]
G_{corr} = 0.17586375 au [CPCM$_{CH2Cl2}$/PBEh-3c]

C	0.689135	-0.053789	-0.991165	O	2.498380	-1.411731	-1.855045
C	0.075188	0.560586	0.083262	H	-0.837611	1.121651	-0.067618
C	0.619403	0.461726	1.369780	H	0.261156	0.026623	-1.982053
C	1.804325	-0.258768	1.556216	H	2.250948	-0.351218	2.537108
C	2.426942	-0.868852	0.481269	H	3.349570	-1.414757	0.627876
C	1.857602	-0.758042	-0.770200	H	0.414890	0.949533	3.233401
O	-0.026921	1.068135	2.359242	S	2.327392	0.778359	5.393559

O	3.170716	-0.265520	4.814912
O	0.974192	0.867094	4.805864
O	2.347233	0.888721	6.845460
C	3.117323	2.339054	4.819000
F	2.453367	3.398710	5.271044
F	3.134045	2.395321	3.487840
F	4.371809	2.414016	5.254076
P	2.176361	-3.104369	-2.463040
C	0.358704	-3.086028	-2.420334
H	-0.041143	-2.419219	-3.182509
H	0.050360	-4.101826	-2.681475
H	-0.062998	-2.843884	-1.445490
C	2.550264	-3.977613	-0.913890
H	2.342896	-5.032302	-1.110853
H	3.607631	-3.892375	-0.668780
H	1.938840	-3.665616	-0.067930
H	2.910642	-0.818109	-2.506976

(*b′*)‡

E = -1765.221923663 au [CPCM$_{CH2Cl2}$/PWPB95-D3/def2-QZVPP]
ZPE = 0.21511253 au [CPCM$_{CH2Cl2}$/PBEh-3c]
G_{corr} = 0.16762236 au [CPCM$_{CH2Cl2}$/PBEh-3c]
υ = -287.96 cm^{-1} [CPCM$_{CH2Cl2}$/PBEh-3c]

C	-0.217743	-0.571094	0.219479
C	-0.203038	-0.641294	1.566357
C	1.013573	-0.398565	2.284741
C	2.220740	-0.089262	1.577413
C	2.202767	-0.019546	0.229359
C	0.977265	-0.213395	-0.522267
O	0.960238	-0.479071	3.558169
O	0.951698	-0.071878	-1.758522
H	-1.094120	-0.878212	2.131970
H	-1.133151	-0.743245	-0.332473
H	3.143003	0.067168	2.121123
H	3.107916	0.201810	-0.321602
H	1.834760	-0.291723	4.073410
S	4.183302	0.739006	4.896782
O	4.886041	0.585856	3.627690
O	2.938696	-0.073357	4.997304
O	4.984767	0.697337	6.106452
C	3.522054	2.455642	4.833486
F	2.847819	2.741295	5.940347
F	2.701162	2.593546	3.793296
F	4.515025	3.326633	4.703983
P	1.290356	-2.241413	-2.872873
C	1.957624	-3.124988	-1.424271
H	3.028666	-2.947190	-1.341696
H	1.479104	-2.786618	-0.503529
H	1.780917	-4.197667	-1.505616
C	-0.416335	-2.865082	-3.055890
H	-1.028300	-2.504199	-2.229944
H	-0.845014	-2.491300	-3.983989
H	-0.446370	-3.954487	-3.061358
H	1.967219	-2.879198	-3.934781

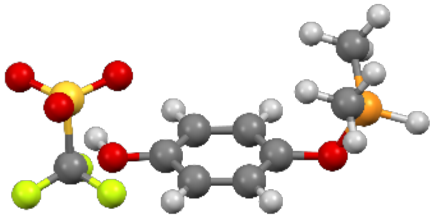

II

E = -1765.321806193 au [CPCM$_{CH2Cl2}$/PWPB95-D3/def2-QZVPP]
ZPE = 0.22004221 au [CPCM$_{CH2Cl2}$/PBEh-3c]
G_{corr} = 0.17350632 au [CPCM$_{CH2Cl2}$/PBEh-3c]

C	-0.201888	-0.257431	-0.156263
C	-0.440320	-0.048760	1.190391
C	0.610730	-0.062063	2.112220
C	1.911879	-0.280690	1.651285
C	2.151264	-0.489691	0.302532
C	1.094374	-0.480824	-0.588843
O	0.312705	0.141244	3.395726
O	1.339836	-0.664955	-1.956494
H	-1.450387	0.129180	1.536125
H	-1.023055	-0.231609	-0.861541
H	2.749644	-0.290949	2.335943
H	3.165836	-0.641897	-0.043925
H	1.108043	0.118253	3.975180
S	3.728212	0.509383	5.154913

O	4.527701	-0.030516	4.057127	H	3.624247	-2.618877	-2.014033
O	2.316127	0.074821	5.155198	H	2.505261	-3.308191	-0.809092
O	4.351237	0.490682	6.471463	H	2.696666	-4.084781	-2.390647
C	3.598645	2.301612	4.752747	C	-0.223051	-2.961420	-2.300643
F	2.885441	2.942332	5.674391	H	-0.393356	-3.137520	-1.239776
F	3.006938	2.478229	3.572179	H	-1.028602	-2.355093	-2.710709
F	4.806791	2.855413	4.699015	H	-0.199908	-3.920942	-2.817685
P	1.345762	-2.150751	-2.552562	H	1.561103	-1.951242	-3.912009
C	2.672771	-3.127438	-1.869663				

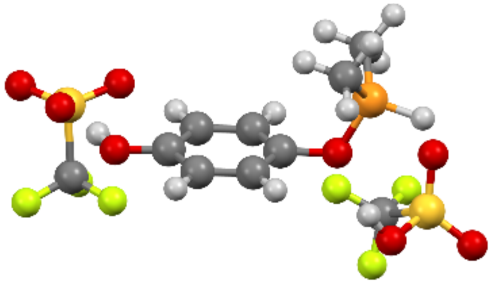

II·HOTf E = -2727.428429570 au [CPCM$_{CH2Cl2}$/PWPB95-D3/def2-QZVPP]
ZPE = 0.26111555 au [CPCM$_{CH2Cl2}$/PBEh-3c]
G_{corr} = 0.20531350 au [CPCM$_{CH2Cl2}$/PBEh-3c]

C	0.019922	-0.063626	0.016051	F	5.470856	3.152861	4.238397
C	0.050936	-0.092636	1.397780	P	1.365900	-1.325441	-2.919314
C	1.268683	-0.061471	2.086122	C	0.266644	-2.581726	-2.297277
C	2.458562	0.008956	1.355761	H	-0.762540	-2.228437	-2.321285
C	2.429173	0.040940	-0.028805	H	0.357304	-3.453964	-2.945552
C	1.212664	-0.000595	-0.682099	H	0.541042	-2.866798	-1.283388
O	1.228113	-0.097850	3.415841	C	3.057277	-1.881611	-2.859330
O	1.172389	0.050995	-2.090949	H	3.154454	-2.727912	-3.540410
H	-0.875293	-0.139253	1.955451	H	3.734709	-1.091983	-3.175619
H	-0.927997	-0.080373	-0.506458	H	3.310196	-2.212363	-1.853227
H	3.417518	0.040088	1.855459	H	0.961339	1.710307	-2.865973
H	3.358916	0.108984	-0.578798	H	1.005164	-0.983635	-4.217198
H	2.124647	-0.064009	3.823601	S	2.375450	2.401410	-4.334669
S	4.847644	0.621456	4.518304	O	1.099492	2.512224	-3.417129
O	5.455454	0.364655	3.214283	O	2.765542	1.019159	-4.499267
O	3.541433	-0.038211	4.724476	O	2.184390	3.279858	-5.452077
O	5.737311	0.539379	5.668445	C	3.647182	3.175655	-3.246294
C	4.390553	2.403368	4.438358	F	4.804572	3.141520	-3.877695
F	3.813818	2.794039	5.571058	F	3.734196	2.489178	-2.119767
F	3.538066	2.626631	3.439047	F	3.314428	4.422076	-2.984657

(*c*)‡ E = -2727.374090662 au [CPCM$_{CH2Cl2}$/PWPB95-D3/def2-QZVPP]
ZPE = 0.25422577 au [CPCM$_{CH2Cl2}$/PBEh-3c]
G_{corr} = 0.19939545 au [CPCM$_{CH2Cl2}$/PBEh-3c]
υ = -822.30 cm^{-1} [CPCM$_{CH2Cl2}$/PBEh-3c]

C	-2.141766	-0.196918	0.628958	F	3.550029	3.083253	4.433152
C	-2.069312	-0.148923	2.008593	P	-1.022532	-1.664582	-2.456830
C	-0.830222	-0.122115	2.658264	C	-2.283445	-2.709988	-1.707368
C	0.340072	-0.138616	1.892475	H	-3.264088	-2.244085	-1.775711
C	0.269703	-0.183050	0.510559	H	-2.307472	-3.648950	-2.261986
C	-0.967473	-0.215420	-0.101745	H	-2.043535	-2.930387	-0.667502
O	-0.831285	-0.080011	3.987937	C	0.551786	-2.453534	-2.074621
O	-1.034844	-0.242624	-1.508072	H	0.589751	-3.381388	-2.648387
H	-2.978593	-0.129706	2.595036	H	1.384489	-1.829793	-2.393952
H	-3.104940	-0.208977	0.135153	H	0.647989	-2.697498	-1.017796
H	1.313741	-0.117270	2.363341	H	-1.212630	0.775044	-2.184101
H	1.180784	-0.180424	-0.073936	H	-1.110677	-0.754997	-4.025967
H	0.077716	-0.042204	4.367068	S	-0.944118	1.496286	-4.388683
S	2.840795	0.609222	4.933358	O	-1.344287	1.634820	-2.940717
O	3.401980	0.225921	3.639526	O	-1.029859	0.044542	-4.826002
O	1.517473	0.019791	5.224923	O	-1.574627	2.421380	-5.274929
O	3.758597	0.584393	6.063862	C	0.872248	1.835133	-4.360859
C	2.448537	2.395824	4.721479	F	1.362934	1.660532	-5.565272
F	1.920644	2.898389	5.833691	F	1.433336	0.992323	-3.511309
F	1.576018	2.572774	3.730254	F	1.057084	3.071832	-3.961883

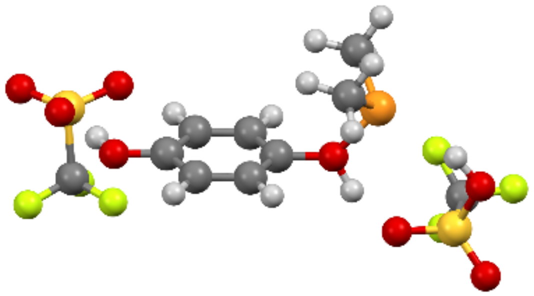

I·HOTf E = -2727.383655189 au [$CPCM_{CH2Cl2}$/PWPB95-D3/def2-QZVPP]
ZPE = 0.26140506 au [$CPCM_{CH2Cl2}$/PBEh-3c]
G_{corr} = 0.20575558 au [$CPCM_{CH2Cl2}$/PBEh-3c]

C	-0.046939	-0.092568	0.056037	O	3.476075	0.064770	4.754014
C	-0.011974	-0.062117	1.436738	O	5.691801	0.617425	5.665385
C	1.210015	-0.042206	2.120318	C	4.419405	2.448061	4.312257
C	2.402634	-0.048784	1.387946	F	3.859943	2.934158	5.416205
C	2.372108	-0.075488	0.004838	F	3.574711	2.638109	3.299491
C	1.149944	-0.097001	-0.634678	F	5.527695	3.140136	4.064642
O	1.171522	-0.016726	3.447589	P	1.095989	-1.558755	-3.114126
O	1.130272	-0.129717	-2.051402	C	-0.181758	-2.551223	-2.305625
H	-0.936861	-0.052501	1.998070	H	-1.153926	-2.069154	-2.390221
H	-0.994970	-0.102306	-0.465755	H	-0.229819	-3.494196	-2.854648
H	3.362263	-0.033179	1.886844	H	0.042957	-2.773599	-1.263243
H	3.297130	-0.067552	-0.556685	C	2.632692	-2.360689	-2.594336
H	2.069306	0.016464	3.855589	H	2.696546	-3.296392	-3.153719
S	4.807239	0.658845	4.509361	H	3.497756	-1.756614	-2.861760
O	5.404985	0.294227	3.226621	H	2.652379	-2.597132	-1.531071

H	0.939347	0.724247	-2.553628	O	0.325211	2.721265	-5.985329
H	0.680521	-0.434783	-5.104620	C	2.754904	1.778055	-5.410490
S	0.926474	1.718723	-5.162217	F	3.030768	1.656216	-6.689176
O	0.729670	1.743889	-3.715877	F	3.302511	0.782511	-4.732148
O	0.579931	0.312425	-5.753309	F	3.197928	2.928963	-4.954171

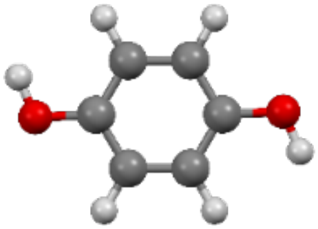

HQ E = -382.639734134 au [$CPCM_{CH2Cl2}$/PWPB95-D3/def2-QZVPP]
ZPE = 0.11160985 au [$CPCM_{CH2Cl2}$/PBEh-3c]
G_{corr} = 0.08098405 au [$CPCM_{CH2Cl2}$/PBEh-3c]

C	-0.011440	-0.020467	0.009524	H	1.211319	0.006988	3.172558
C	0.009067	-0.022644	1.400671	H	3.325919	0.072927	-0.552202
C	1.212330	0.009306	2.089741	H	1.196072	0.016727	-1.763211
C	2.418812	0.044206	1.399830	O	-1.158356	-0.050905	-0.710348
C	2.398299	0.046413	0.008672	H	-1.919237	-0.074331	-0.121048
C	1.195040	0.014439	-0.680393	O	3.565702	0.074628	2.119737
H	-0.918567	-0.049112	1.961525	H	4.326598	0.097893	1.53044

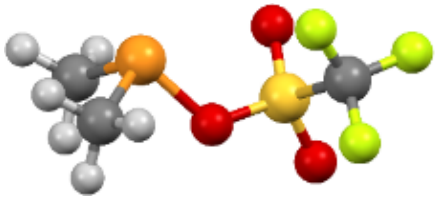

TfO-PMe_2 E = -1382.645001507 au [$CPCM_{CH2Cl2}$/PWPB95-D3/def2-QZVPP]
ZPE = 0.10790118 au [$CPCM_{CH2Cl2}$/PBEh-3c]
G_{corr} = 0.06900973 au [$CPCM_{CH2Cl2}$/PBEh-3c]

S	0.628086	0.048807	0.030265	C	4.260741	2.028668	0.010378
O	0.447399	-0.340887	1.406810	H	3.769656	2.920290	-0.376741
O	2.096677	0.533113	-0.228190	H	5.098698	2.355368	0.628808
O	0.244487	-0.807650	-1.058729	H	4.649162	1.434293	-0.816267
C	-0.280298	1.637057	-0.202640	C	4.082692	-0.444779	1.329825
F	-0.130425	2.059985	-1.443360	H	4.916603	-0.213737	1.995276
F	0.190318	2.547156	0.633224	H	3.468015	-1.198237	1.820442
F	-1.560088	1.428301	0.045526	H	4.479327	-0.844302	0.396714
P	3.126958	1.092431	1.089971				

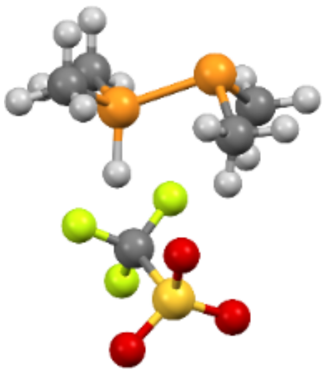

$[Me_2P\text{-}P(H)Me_2]^+$ $[TfO]^-$

E = -1804.401651694 au [CPCM$_{CH2Cl2}$/PWPB95-D3/def2-QZVPP]
ZPE = 0.19610798 au [CPCM$_{CH2Cl2}$/PBEh-3c]
G_{corr} = 0.15026835 au [CPCM$_{CH2Cl2}$/PBEh-3c]

P	-0.302817	-0.055417	-0.097747
C	-0.285519	-0.035127	1.705374
H	0.741678	-0.044605	2.065463
H	-0.783208	0.858941	2.075559
H	-0.807426	-0.915502	2.075998
C	0.606132	-1.497024	-0.687045
H	0.662149	-1.485789	-1.773713
H	1.615057	-1.490090	-0.278624
H	0.090846	-2.399953	-0.364199
P	-2.372034	-0.129691	-0.828357
C	-2.803648	1.595223	-0.322903
H	-3.795174	1.801986	-0.721926
H	-2.865091	1.669696	0.762199
H	-2.112700	2.348468	-0.700105
C	-1.941005	0.107537	-2.608785
H	-1.526036	-0.810604	-3.020476
H	-2.871539	0.310452	-3.134157
H	-1.253148	0.934205	-2.782526
H	0.394594	1.080159	-0.532911
S	-5.331455	-1.569799	-2.178675
O	-4.146300	-2.360934	-2.506600
O	-5.065565	-0.167806	-1.823910
O	-6.480312	-1.763562	-3.060194
C	-5.903312	-2.294637	-0.584986
F	-6.995699	-1.668891	-0.148105
F	-4.968430	-2.195968	0.357612
F	-6.199697	-3.585086	-0.736836

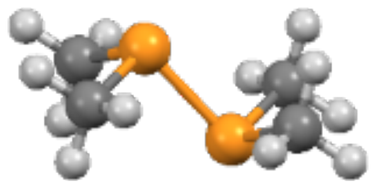

$Me_2P\text{-}PMe_2$ E = -842.258874115 au [CPCM$_{CH2Cl2}$/PWPB95-D3/def2-QZVPP]
ZPE = 0.15648769 au [CPCM$_{CH2Cl2}$/PBEh-3c]
G_{corr} = 0.12216199 au [CPCM$_{CH2Cl2}$/PBEh-3c]

P	0.047777	0.054011	-0.029392
C	0.002514	-0.003165	1.820343
H	1.024487	0.002986	2.200868
H	-0.498015	0.882249	2.211720
H	-0.504891	-0.891192	2.198880
C	0.709387	-1.639528	-0.377345
H	0.679570	-1.842091	-1.447747
H	1.753051	-1.684281	-0.064306
H	0.155248	-2.420386	0.144701
P	-2.094476	-0.348175	-0.418829
C	-2.756090	1.345373	-0.070676
H	-3.799789	1.390361	-0.383530
H	-2.726013	1.547750	0.999758
H	-2.201894	2.126210	-0.592694

C	-2.049246	-0.290921	-2.268601
H	-1.548631	-1.176339	-2.659865
H	-3.071142	-0.297044	-2.649307
H	-1.541755	0.597098	-2.647037

References

1. J. Heinicke, I. Böhle, A. Tzschach, 1,3-Carbanionische Umlagerungen: Reaktionen von Phosphorsäure-o-haloarylestern mit Metallen zu Arylphosphonsäurederivaten *J. Organomet. Chem.* **1986**, 317, 11-21.

2. Ghalib, M.; Jones, P. G.; Heinicke, J. W. Solvent-controlled lithiation of PC–N-heterocycles: Synthesis of mono- and bis(trimethylsilyl)-tert-butyl-dihydrobenzazaphospholes – A new type of highly bulky and basic phosphine ligands. *J. Org. Chem.* **2014**, *763-764*, 44-51. DOI:10.1016/j.jorganchem.2014.04.014.

3. (a) Boone, H. W.; Bryce, J.; Lindgren, T.; Padias, A. B.; Hall, H. K., Jr Stereoregular Poly(benzoquinone imines) from Methyl-Substituted Benzoquinones. *Macromolecules* **1997**, *30* (9), 2797-2799. DOI: 10.1021/ma961236j. (b) Dibattista, J.; Schmidt, B. M.; Buyle Padias, A.; Hall, H. K. Substituent effects on the polycondensation of quinones with aromatic amines to form poly(quinone imine)s. *J. Polym. Sci. Part A: Polym. Chem.* **2002**, *40*(1), 43-54. DOI: 10.1002/pola.10089.

4. F. Neese, "The ORCA program system." *Wiley Interdiscip. Rev. Comput. Mol. Sci.*, **2012**, *2*, 73–78. DOI: 10.1002/wcms.81

5. C. Lee, W. Yang and R. G. Parr, "Development of the Colle-Salvetti correlation-energy formula into functional of the electron density." *Phys. Rev. B*, **1988**, *37*, 785–789. DOI: 10.1103/PhysRevB.37.785.

6. A. D. Becke, "Density-functional thermochemistry. III. The role of exact exchange." *J. Chem. Phys.*, **1993**, *98*, 5648–5652. DOI: 10.1063/1.464913.

7. F. Neese, F. Wennmohs, A. Hansen and U. Becker, "Efficient, approximate and parallel Hartree-Fock and hybrid DFT calculations. A 'chain-of-spheres' algorithm for the Hartfree-Fock exchange." *Chem. Phys.*, **2009**, *356*, 98–109. DOI: 10.1016/j.chemphys.2008.10.036.

8. F. Weigend and R. Ahlrichs, "Balanced basis sets of split valence, triple zeta valence and quadruple zeta valence quality for H to Rn: Design and assessment of accuracy." *Phys. Chem. Chem. Phys.*, **2005**, *7*, 3297–3305. DOI: 10.1039/B508541A.

9. E. Caldeweyher, J. M. Mewes, S. Ehlert and S. Grimme, "Extension and evaluation of the D4 London-dispersion model for periodic system." *Phys. Chem. Chem. Phys.*, **2020**, *22*, 8499–8512. DOI: 10.1039/D0CP00502A.

10. A. Schäfer, C. Huber and R. Ahlrichs, "Fully optimized contracted Gaussian basis sets of triple zeta valence quality for atoms Li to Kr." *J. Chem. Phys.*, **1994**, *100*, 5829–5835. DOI: 10.1063/1.467146.

11. C. Riplinger, B. Sandhoefer, A. Hansen and F. Neese, "Natural triple excitations in local coupled cluster calculations with pair natural orbitals." *J. Chem. Phys.*, **2013**, *139*, 134101−134113. DOI: 10.1063/1.4821834.

12. J. A. Pople, M. Head-Gordon and K. Raghavachari, "Quadratic configuration interaction. A general technique for determining electron correlation energies." *J. Chem. Phys.*, **1987**, *87*, 5968–5975. DOI: 10.1063/1.453520.

13. V. Barone , M. Cossi, "Quantum calculation of molecular energies and energy gradients in solution by a conductor solvent model."*J. Phys. Chem. A*, **1998**, *102*, 1995-2001. DOI: 10.1021/jp9716997.

14. M. Cossi, N. Rega, G. Scalmani, V. Barone, "Energies, structures, and electronic properties of molecules in solution with the C-PCM solvation model," *J. Comp. Chem.*, **2003**, *24*, 669-681. DOI: 10.1002/jcc.10189.

15. S. Grimme, J. G. Brandenburg, C. Bannwarth, A. Hansen, "Consistent structures and interactions by density functional theory with small atomic orbital basis sets" *J. Chem. Phys.*, **2015**, *143*, 054107. DOI: 10.1063/1.4927476.

16. L. Goerigk and S. Grimme, "Efficient and accurate double-hybrid-meta-GGA density functionals-evaluations with the extended GMTKN30 database for general main group thermochemistry, kinetics, and noncovalent interactions." *J. Chem. Theory Comput.*, **2011**, *7*, 291–309. DOI: 10.1021/ct100466k.

17. L. Goerigk and S. Grimme, "A thorough benchmark of density functional methods for general main group thermochemistry, kinetics, and noncovalent interactions." *Phys. Chem. Chem. Phys.*, **2011**, *13*, 6670–6688. DOI: 10.1039/C0CP02984J.

18. S. Grimme, J. Antony, S. Ehrlich and H. Krieg, “A consistent and accurate ab initio parametrization of density functional dispersion correction (DFT-D) for the 94 elements H-Pu.” *J. Chem. Phys.*, **2010**, *132*, 154104–154119. DOI: 10.1063/1.3382344.

19. S. Grimme, S. Ehrlich and L. Goerigk, “Effect of the damping function in dispersion corrected density functional theory.” *J. Comput. Chem.*, **2011**, *32*, 1456–1465. DOI: 10.1002/jcc.21759

20. A. D. Becke, E. R- Johnson, “A density-functional model of the dispersion interaction.” *J. Chem. Phys.*, **2005**, *122*, 154101, DOI: 10.1063/1.2065267.

21. E. R. Johnson, A. D. Becke, “A post-Hartree–Fock model of intermolecular interactions.” *J. Chem. Phys.*, **2005**, *123*, 024101, DOI: 10.1063/1.1949201.

22. E. R. Johnson, A. D. Becke, “A post-Hartree-Fock model of intermolecular interactions: Inclusion of higher-order corrections.” *J. Chem. Phys.*, **2006**, *124*, 174104, DOI: 10.1063/1.2190220.

23. Y. Zhao, D. G. Truhlar, "The M06 suite of density functionals for main group thermochemistry, thermochemical kinetics, noncovalent interactions, excited states, and transition elements: Two new functionals and systematic testing of four M06-class functionals and 12 other functionals." *Theor. Chem. Acc.*, **2008**, *120*, 215–241, DOI:10.1007/s00214-007-0310-x.

24. F. London, “The quantic theory of inter-atomic currents in aromatic combinations.” *J. Phys. Radium*, **1937**, *8*, 397-409. DOI: 10.1051/jphysrad:01937008010039700.

25. J. R. Cheeseman, G. W. Trucks, T. A. Keith, and M. J. Frisch, “A Comparison of Models for Calculating Nuclear Magnetic Resonance Shielding Tensors” *J. Chem. Phys.*, **1996**, *104*, 5497-509. DOI: 10.1063/1.471789.

26. C. Adamo and V. Barone, “Toward reliable density functional methods without adjustable parameters: The PBE0 model,” *J. Chem. Phys.*, **1999**, *110*, 6158-6169. DOI: 10.1063/1.478522.

27. J. Tomasi, B. Mennucci, and R. Cammi, “Quantum mechanical continuum solvation models.” *Chem. Rev.*, **2005**, *105*, 2999-3093, DOI: 10.1021/cr9904009.

28. Gaussian 16, Revision C.01, M. J. Frisch, G. W. Trucks, H. B. Schlegel, G. E. Scuseria, M. A. Robb, J. R. Cheeseman, G. Scalmani, V. Barone, G. A. Petersson, H. Nakatsuji, X. Li, M. Caricato, A. V. Marenich, J. Bloino, B. G. Janesko, R. Gomperts, B. Mennucci, H. P. Hratchian, J. V. Ortiz, A. F. Izmaylov, J. L. Sonnenberg, D. Williams-Young, F. Ding, F. Lipparini, F. Egidi, J. Goings, B. Peng, A. Petrone, T. Henderson, D. Ranasinghe, V. G. Zakrzewski, J. Gao, N. Rega, G. Zheng, W. Liang, M. Hada, M. Ehara, K. Toyota, R. Fukuda, J. Hasegawa, M. Ishida, T. Nakajima, Y. Honda, O. Kitao, H. Nakai, T. Vreven, K. Throssell, J. A. Montgomery, Jr., J. E. Peralta, F. Ogliaro, M. J. Bearpark, J. J. Heyd, E. N. Brothers, K. N. Kudin, V. N. Staroverov, T. A. Keith, R. Kobayashi, J. Normand, K. Raghavachari, A. P. Rendell, J. C. Burant, S. S. Iyengar, J. Tomasi, M. Cossi, J. M. Millam, M. Klene, C. Adamo, R. Cammi, J. W. Ochterski, R. L. Martin, K. Morokuma, O. Farkas, J. B. Foresman, and D. J. Fox, Gaussian, Inc.: Wallingford CT, 2016.

29. T. Lu, F. Chen, Multiwfn: A Multifunctional Wavefunction Analyzer, *J. Comput. Chem*. **2012**, *33*, 580-592, DOI: 10.1002/jcc.22885.

30. NCIplot, version 1.1.x, Department of Chemistry, Duke University (USA), http://www.chem.duke.edu/~yang/Software/softwareNCI.html. **2011** (accessed: 2025).

31. E. R. Johnson, S. Keinan, P. Mori-Sánchez, J. Contreras García, A. J. Cohen, W. Yang, “Revealing noncovalent interactions.” *J. Am Chem. Soc.*, 2010, *132*, 6498-6506. DOI: 10/1021/ja100936w.

32. J. Contreras-García, E. R. Johnson, S. Keinan, R. Chaudret, J.-P. Piquemal, D. N. Beratan, W. Yang, “NCIPLOT: a program for plotting non-covalent interaction regions.” *J. Chem. Theory Comput.*, **2011**, *7*, 625-632. DOI: 10.1021/ct100641a.

33. A. Rey, A. Espinosa Ferao, R. Streubel, “Quantum Chemical calculations on CHOP derivatives – spanning the chemical space of phosphinidenes, phosphaketenes, oxaphosphirenes and COP^- isomers.” *Molecules*, **2018**, *23(12)*, 3341. DOI: 10.3390/molecules23123341.

34. A. Rey Planells, A. Espinosa Ferao, “Accurate ring strain energy calculations on saturated three-membered heterocycles with one group 13-16 element.” *Inorg. Chem*, **2020**, *59*, 11503-11513, DOI: 10.1021/acs.inorgchem.0c01316.

35. A. Espinosa Ferao, A. Rey Planells, R. Streubel, “Between oxirane and phosphirane: the spring loaded oxaphosphirane ring.” *Eur. J. Inorg. Chem.*, **2021**, *22(4)*, 348-353. DOI: 10.1002/ejic.202000881.

36. A. Rey Planells, A. Espinosa Ferao, “CHNO isomers and derivatives - A computational overview.” *New J. Chem.*, **2022**, *46*, 5771-5778, DOI: 10.1039/D1NJ05752A.

37. A. Rey Planells, A. Espinosa Ferao, “Accurate Ring Strain Energies of Unsaturated Three-Membered Heterocycles with One Group 13−16 Element.” *Inorg. Chem.*, **2022**, *61(17)*, 6459-6468. DOI: 10.1021/acs.inorgchem.2c00067.

38. A. Rey Planells, A. Espinosa Ferao, “Ring Strain Energies of Three-membered Homoatomic Inorganic rings El_3 and Diheterotetreliranes El_2Tt (Tt = C, Si, Ge). Accurate versus Additive Approaches.” *Inorg. Chem.*, **2022**, *61(35)*, 13846-13857. DOI: 10.1021/acs.inorgchem.2c01777.

39. S. E. Wheeler, K. N. Houk, P. v. R. Schleyer, W. D. Allen, “A Hierarchy of Homodesmotic Reactions for Thermochemistry.” *J. Am. Chem. Soc.*, **2009**, *131(7)*, 2547-2560. DOI: 10.1021/ja805843n.

40. A. M. Stone, A. R. Golden, S. M. Daniel, A. L. Rheingold, J. D. Protasiewicz, “Hydrogen Bonding vs Dihydrogen Bonding in the Air Stable Primary Phosphine *ortho*-Phosphinophenol.” *Eur. J. Inorg. Chem.* **2024**, *25*, e202400260.

41. S. Yourdkhani, M. Jablonski, J. Echeverria, “Attractive PHHP interactions revealed by state-of-the-art ab initio calculations.” *Phys. Chem. Chem. Phys.* **2017**, *19* (41), 28044-28055. DOI: 10.1039/c7cp04412g.
